# Causes of medication errors in community pharmacies: A meta-ethnography and systematic review

**DOI:** 10.1371/journal.pone.0349120

**Published:** 2026-06-10

**Authors:** Maguy Saffouh El Hajj, Anna Robinson-Barella, Andy Husband, Adam Todd

**Affiliations:** 1 Clinical Pharmacy and Practice Department, College of Pharmacy, QU Health, Qatar University, Doha, Qatar; 2 School of Pharmacy, Newcastle University, Newcastle upon Tyne, United Kingdom; 3 Newcastle NIHR Patient Safety Research Collaborative (PSRC), School of Pharmacy, Newcastle University, Newcastle upon Tyne, United Kingdom; Universiti Monash Malaysia: Monash University Malaysia, MALAYSIA

## Abstract

**Introduction:**

Medication errors are a leading cause of patient harm and account for thousands of deaths and injuries annually. While medication safety efforts have primarily targeted hospital settings, community pharmacies are also prone to such errors due to their high patient access and utilisation. This review utilised a meta-ethnographic qualitative approach to synthesise findings from available primary qualitative research about the causes of medication safety issues and errors in community pharmacies as well as the contributing factors.

**Materials and methods:**

This meta-ethnographic systematic review was conducted in line with Preferred Reporting Items for Systematic reviews and Meta-Analyses (PRISMA) and the meta-ethnography reporting guidance (eMERGe) and framework and registered on PROSPERO. Eligible studies were identified using a comprehensive search strategy across multiple databases and grey literature sources, without language or date restrictions. Qualitative data were synthesised using Noblit and Hare’s seven-phase meta-ethnographic approach, generating third-order constructs that captured underlying causes of medication errors in community pharmacies. Confidence in the findings was assessed using the GRADE-CERQual framework.

**Results:**

Thirteen studies were included in the review with the majority from the United States and England. Most studies (n = 10) used qualitative research designs while two studies utilised a mixed-methods approach. Through meta-ethnographic synthesis, five third-order constructs (themes) were developed to represent the factors contributing to medication errors in community pharmacies. These themes included: 1) pharmacist-related factors, 2) the environment within the pharmacy 3) management and financial related factors 4) organisational and social environment within the pharmacy and 5) challenges with digital technologies.

**Conclusions:**

This meta-ethnographic systematic review identified five themes contributing to medication errors in community pharmacies. The review findings offer valuable insight for guiding the design of future tailored safety initiatives. Understanding the complex interplay between these contributing factors is essential for enhancing medication safety and informing future research and practice in real-life community pharmacy settings.

## Introduction

According to the World Health Organization (WHO) 2023 data, approximately 10% of patients experience harm while receiving health care with unsafe practice causing over 3 million deaths occurring each year worldwide. Medications account for more than 50% of this harm [1]. Medication-related harm impacts about one in every 30 patients receiving health care and over a quarter of these incidents are considered severe or potentially life-threatening [2]. A medication error is defined as “any preventable event that may cause or lead to inappropriate medication use or patient harm while the medication is in the control of the health-care professional, patient, or consumer [3]. These events could be related to professional practice, products, procedures, and systems and can occur at any stage of the medication-use process, including prescribing, dispensing, administration, education, monitoring, and use [3]. Medication errors can cause adverse drug events, leading to improper medication use, prolonged hospitalisation and adverse consequences for patients. They also cost globally an estimated 42 billion USD annually [4]. Medication safety refers to the procedures used in different healthcare settings to decrease the risk for medication errors [5]. Numerous organisations have started initiatives and published policies to improve medication safety. For example, the WHO launched the third Global Patient Safety Challenge on Medication Safety ‘medication without harm’ with the aim of having a global commitment and action to decrease medication-related harm. The aim of this initiative was to reduce errors by targeting the harm resulting from risky medical practices [4].

Although hospital settings have received a lot of attention when it comes to medication errors and medication safety, non-hospital settings specifically community pharmacies, are also a concern [6]. Due to the extended opening times, geographical accessibility, and convenience, community pharmacies are one of the most frequently used healthcare facilities [7]. For instance, in England, it is estimated that 1.6 million visits are made to community pharmacies every day, with approximately 43 million visits annually for health-related concerns [8].

In addition to dispensing prescribed and over the counter medications and delivering medication therapy services, community pharmacies play an important role in delivering clinical and health promotion interventions [9–14]. These include the management of chronic diseases such as diabetes and hypertension, treatment of minor illness, smoking cessation programs, immunisations, and contraception services [9–14]. Medication errors in community pharmacies involve both prescription and OTC medications and include but not limited to prescription commission errors (e.g., incorrect medication, dose, dosage form, or route of administration), prescription omission errors, and dispensing errors. Dispensing errors may involve content errors (such as dispensing the wrong strength or dosage form) and labelling errors [15].

While there is no estimate of the global incidence of medication errors in community pharmacies, a recent systematic review of 73 studies about the prevalence, nature and severity of medication errors in community pharmacies indicated high variations in error rates and types in different geographical regions [15]. For instance, within the Europe and Central Asia region, the prescribing error rates ranged from 0.062% in a study in the United Kingdom [16] to 32.84% in a study in Poland [17]. While in North America, dispensing error rates varied from 0.075% in a study in New Jersey in the United States [18] to 24% in another American study in New York, New Jersey and Florida [19]. In South Asia, a study from India reported a prescribing error rate of 6.09% [20]. In the Europe and Central Asia region, dispensing error rates ranged from 7.1 undocumented dispensing errors per 100,000 dispensed prescriptions in Finland [21] to 3.3% in the United Kingdom (UK) [22]. In the Middle East and North Africa region, dispensing error rates varied from 0.8% in two studies conducted in Yemen [23,24] to 36.7% in a study in Iran [25].To promote medication safety and prevent medication errors, it is crucial to understand, recognise and target the factors that contribute to errors in community pharmacies. Diverse descriptive studies and reports highlighted several important contributing factors for medication errors across the medication use process in community pharmacies [4,26–29]. For instance, these factors could include work environment factors (e.g., staffing levels and workload), organizational and management factors (e.g.,: financial resources and organizational structure), team factors (e.g., supervision, and team structure), task and technology factors (e.g., decision support tools), individual factors (e.g., knowledge and skills) and patient-related factors (e.g., medical condition and language) [30].

Additionally, some qualitative studies explored how these factors interact in community pharmacy settings. For instance, Phipps *et al.,* in 2009, examined how interactions between people, tasks, equipment and organisational factors can cause medication errors in community pharmacies in the North West of England [31]. Using focus groups with community pharmacists, various themes were generated in relation to the impact of social and organisational factors on medication safety: relationships involving the pharmacist, demands on the pharmacist and management and governance of pharmacists [31]. Furthermore, Harvey *et al.,* investigated risks in prescription dispensing in community pharmacies in England by considering the links between the important elements of the medication dispensing process [32]. Using observations, shadowing and interviews, key categories included: people and their approach to work, management structures, physical infrastructure, engagement with technologies, attitudes towards safety and prescriber influences [32]. Similarly, Al Juffali *et al.,* examined the medication safety problems linked to medication supply in community pharmacies in the Kingdom of Saudi Arabia using focus groups and individual interviews. Several themes were identified, based on the Human Factor Framework (HFF), including commercialism and commercial pressure, patient factors, Illegal supply of medications, lack of enforcement of regulations, the healthcare system, patient medication taking behaviour and other themes [33].

While individual qualitative studies have provided essential information about the factors affecting medication safety in community pharmacy, this evidence remains fragmented. There still remains a need to gain further and deeper insight of how and why these errors occur to better inform improvement efforts. A qualitative evidence synthesis approach, such as meta-ethnography, can assist in extending beyond original studies and systematic reviews to develop higher order interpretations and identify target areas for improving medication safety in community pharmacy settings.

### Objectives

This review utilised a meta-ethnographic approach to synthesise findings from available primary qualitative research about the causes of medication safety issues and errors in community pharmacies, as well as the contributing factors. The review findings can potentially give a perspective into designing approaches to mitigate potential medication errors from occurring in community pharmacies.

## Materials and methods

This meta-ethnographic systematic review has been conducted and reported according to the PRISMA (Preferred Reporting Items for Systematic Reviews and Meta-Analyses) guidelines [34] and the meta-ethnography reporting guidance (eMERGe) and framework [35] (S1 and S2 File). The review was registered on the International Prospective Register of Systematic Reviews (PROSPERO) at the Centre for Reviews and Dissemination, University of York, United Kingdom (ref: CRD42024562734).

### Eligibility criteria

The review inclusion criteria were formulated using the SPIDER (Sample, Phenomenon of Interest, Design, Evaluation, Research type) framework, as follows:

- S: adult or paediatric patients who are receiving care in community pharmacies;
- PI: medication errors or medication safety problems;
- D: published literature and grey literature of qualitative design reporting primary qualitative data or mixed methods design where sufficient qualitative data is available;
- E: causes of medication errors or factors that contribute to medication errors;
- R: peer reviewed journal articles and grey literature including reports and briefings, conference proceedings and theses.

Reviews, letters, editorials, commentaries, clinical trials and quantitative studies were excluded [36]. Articles were not excluded from this work based on study quality. Language and date restrictions were not applied. A community pharmacy setting was defined as ‘a healthcare facility that provides pharmaceutical and cognitive services to the community’ [37].

### Data sources and search strategy

The following databases and search engines were systematically searched from inception until 31 January 2025: MEDLINE (Ovid), Embase (Ovid), Cochrane Central Register of Controlled Trials, ISI Web of Science, Scopus, Database of Abstracts of Reviews of Effects (DARE), Health System Evidence, Global Health Database, Joanna Briggs Institute Evidence-Based Practice Database, Academic Search Ultimate, ProQuest Dissertations, PROSPERO, Cumulative Index to Nursing and Allied Health Literature (CINAHL) (EBSCO), ScienceDirect (Elsevier), Health Management Information Consortium (HMIC), and Google Scholar. Different search terms were used according to the searched database. Manual backward citation searching of the references of all included articles and other relevant review articles was conducted. Grey literature was also searched through abstracts of conference proceedings and dissertation abstracts. Data sources included theses.com and ProQuest. S3 File outlines the data sources and the search strategy in details.

### Study selection

All obtained references were exported to EndNote^©^20 reference software manager for duplicates removal and screening. MH (Maguy El Hajj) evaluated study titles and abstracts in accordance with the review’s inclusion criteria. Eligible studies were exported to Rayyan^©^ software where full-text screening was undertaken by a single researcher (MH) and independently checked by senior authors AT (Adam Todd) or AH (Andy Husband). Disagreements were resolved through dialogue and achievement of mutual agreement.

### Data extraction and quality appraisal

A data extraction tool was designed for extracting data from included studies including: author(s), publication year, design, setting, country, aims, population, data collection, results including themes, subthemes and authors’ quotes in addition to other relevant information. These data were extracted by one researcher (MH) and checked independently by another researcher (AR-B). Disagreements were addressed in consultation with the senior authors (AH and AT) until consensus was reached.

The quality of each included study was evaluated by MH using Critical Appraisal Skills Programme (CASP) tool for qualitative research [38].

### Analysis and interpretative synthesis

Meta-ethnography was initially developed by Noblit and Hare [39] and is frequently utilised in health and medical research [40–45]. In order to obtain a thorough knowledge, and to guide the creation of more comprehensive concepts, meta-ethnography is an interpretive and inductive methodology that stimulates researchers to comprehend and apply concepts, themes, and metaphors from other studies [35,39]. It was selected as the approach for qualitative evidence synthesis in this study as it allows for interpretative synthesis rather than just aggregating the results of qualitive studies, assists in forming advanced conceptual insights and helps unveil cross study explanations in relation to medication safety in community pharmacy settings [40–45].

The seven phases of the meta-ethnography approach are depicted in Table 1.

**Table 1:** Noblit and Hare’s seven‐step process for meta‐ethnography [39].

Noblit and Hare Step	How It was Applied in this Systematic Review
1-Getting Started	We defined the systematic review aim and objectives focusing on causes of medication errors in community pharmacies as well as the contributing factors
2- Deciding what is relevant	We defined the inclusion and exclusion criteria for selecting the studies, conducted the searches in both databases and other sources, screened titles, abstracts and full texts and identified articles for inclusion
3- Reading the studies	We read the included studies several times and extracted the studies’ relevant information along with first-order and second-order constructs
4-Determining how the studies are relevant	We compared concepts across studies and categorized related concepts and highlighted areas of contention or rejection
5- Translating studies into one another	We translated the studies into one another, i.e.,: we conducted reciprocal translation and considered refutational translations when results were not in agreement
6- Synthesizing the translations	Using an iterative analysis, we moved back and forth with the data to compare and contrast the different findings and translate them into one another. Finally, we constructed third-order constructs by synthesising the translations. These interpretations outlined how contributory factors for medication errors are related
7- Expressing the synthesis	We reported the final themes

*Determining how studies are related*. An excel sheet outlining the results extracted from the included studies was created, including quotations (named ‘first-order constructs’) and the original authors’ interpretations (named ‘second-order constructs’). This sheet helped in comparing the results. Two reviewers (MH and AR-B) then discussed how these constructs are related (Step 4).

*Translating the studies into one another*. Reciprocal translation (*i.e*., chronological comparison of first- and second-order constructs across individual papers) was used to develop new themes and subthemes (named ‘third-order constructs’) [30]. This method entails comparing the themes identified in study 1 with those from study 2, followed by a comparison of the combined themes with the findings from study 3, and subsequent studies. This iterative approach continued until all studies were synthesised. (Step 5)

*Synthesising translations and expressing the synthesis*. Third-order constructs were generated by synthesising first-order constructs (*i.e*., participant direct quotations) and the second-order constructs (*i.e*., original study authors’ interpretations) (Steps 6 and 7). The third-order constructs aim to extend beyond the initial author interpretations of studies to have an overall understanding of the causes of medication errors in community pharmacies.

To align with the reporting guidance of meta-ethnographies, the term ‘theme’ was used to refer to third-order constructs, and ‘sub-themes’ to denote third-order construct sub-themes [46].

### Confidence in the synthesised findings

The GRADE-CERQual (Confidence in the Evidence from Reviews of Qualitative Research) approach was used to assess the confidence in our results [47]. This approach includes four components methodological limitations of included studies, the coherence of the qualitative evidence synthesis findings, the adequacy of data used to support the review finding, and the relevance of the included studies to the review question. According to the results of CERQual, the overall confidence is categorised into three levels: high, moderate, and low [47].

## Results

**Fig 1:**
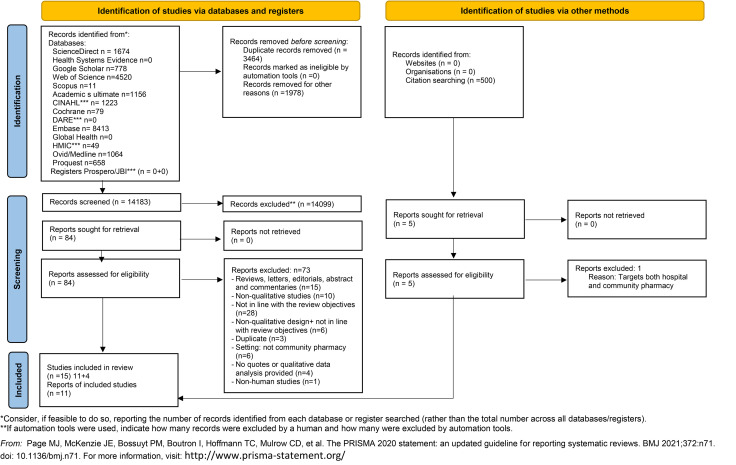
PRISMA 2020 flow diagram for new systematic reviews which included searches of databases, registers and other sources.

Overall, 19,625 eligible studies were identified. After exclusion of duplicates and irrelevant articles, 14,183 studies were screened of which 11 studies met the review inclusion criteria. Four more studies were found through citation searching, resulting in a total of 15 individual studies included in the systematic review (Fig 1). Two articles were excluded from the meta-ethnography synthesis as they were unpublished theses [48,49]. Thirteen articles were included in the meta-ethnography synthesis.

### Studies characteristics

A summary of the included studies’ characteristics is provided in Table 2. The studies were conducted across different countries, including five in the United States [50–55] and five in England [31,32,48,49,56]. Individual studies were carried out in New Zealand [57] and Kingdom of Saudi Arabia [33]. Moreover, one study originated from both the United States and Puerto Rico [58] while another study covered England and Wales [59]. Most studies (n = 10) used qualitative research designs [31–33,49,52,53,55–57,59], while two studies utilised a mixed-methods approach [48,51]. Duration of data collection varied from 2 months [52] to 19 years [57]. Various methods of data collection were employed with nine studies using a combination of approaches [32,33,48–50,52–54,56]. Interviews were the most frequently used method (n = 8 studies) [32,33,48,49,53–55,59], followed by observations or diary fields (n = 4 studies), [32,52–54] focus groups (n = 3 studies) [31,33,56] and think-aloud protocols (n = 2 studies) [52,53].

**Table 2:** Characteristics of Included Studies.

						Data collection				Participant/Population			
Authors/ Year/ Citation	Study aims	Study Setting	Inclusion/ Exclusion criteria	Country/ Number of pharmacies	Study design	Recruitment	Method	Duration and time of data collection	Sample size	Nature	Sex/ Gender	Age (yrs) Mean ± SD or Median ±IQR	Data analysis/ Framework for qualitative collection or analysis
Phipps *et al.,* 2009 [31]	To identify sociotechnical factors that community pharmacy staff encounter in practice, and suggest how these factors might impact on medication safety	Community Pharmacy	NM	England/ NM	Qualitative	Recruited on a purposive basis, having taken part in a previous questionnaire study by authors and consented to be contacted about further research	10 FG (2 hrs each) using semi-structured topic guide	Between December 2003 and April 2004	67 participants	Pharmacists: 10 owner 11 employed 31 locum 10 preregistration pharmacists 5 support staff	NM	NM	Template Analysis/ Realist episte--mological framework
Hincapie *et al.,* 2019 [50]	To examine quantitatively and qualitatively frequency type and contributing factors of e-prescribing quality-related problems reported to two national incident-reporting databases	Community pharmacy	NM	USA/NM	Retrospective analysis of voluntarily reports of e-prescribing quality-related incidents	NA	Convenience sample of all deidentified e-prescribing incidents voluntarily reported by pharmacy personnel to PQC and PEER Portal + descriptive incident data (i.e., open-ended data) to understand reasons for e-prescribing incidents	Between January 2010 and January 2015	NA	NA	NA	NA	Quantitative analysis: descriptive statistics Qualitative analysis: 10% random sample of comments received in the open text fields in each data set/ Odukoya et al’s conceptual framework,
Phipps *et al.,* 2018 [56]	To examine the process and conditions associated with safety improvement in community pharmacy setting	Community pharmacy	NM	England /10	Longitudinal qualitative design	-Recruited on a purposive basis to represent typical range of sizes and organisational structures, i.e., pharmacies with a small number of staff versus large number of staff, independent versus chain pharmacies -Pharmacies came into the sample through: advertisement circulated by county’s local pharmaceutical committees and local professional networks; through a national “research-ready pharmacies” scheme; or by being nominated by their respective pharmacy head offices	-Study began with a MaPSaF workshop at each pharmacy -During the workshop participants read descriptors and, firstly individually and then in group discussion, appraised their pharmacy with respect to depicted developmental levels. Once done, facilitators summarised all responses before reporting them back to group. -Participants agreed on actions that they might carry out to improve quality and safety in their pharmacy -Subsequent MaPSaF workshops were held after 6 months and12 months In addition: -Each pharmacy was visited twice after 1st workshop to obtain informal data about subsequent learning and implementation of improvement activities and organisational context in which it was occurring -FG was held with participants at each pharmacy, 3 months after 1^st^ and 2^nd^ workshops to discuss participants’ experiences of workshop and identify immediate learning or actions that resulted from it.	12 months: between June 2014 and May 2016.	50 members of pharmacy staff	−14 pharmacists −36 members of support staff)	NM	NM	Template analysis/ Manchester Patient Safety Framework (MaPSaF)
Odukoya *et al.,* 2014 [54]	To explore types of e-prescribing errors in community pharmacies and their potential consequences, and the factors that contribute to e-prescribing errors.	Community pharmacy	NM	Wisconsin USA/5	NM (quantitative +qualitative)	-A convenience sample of 5 community retail pharmacies located at separate sites from prescriber offices (3 independent pharmacies 2 chain pharmacies) (2 pharmacies with PDX, 2 pharmacies with Pharmaserv, and 1 pharmacy with Rx30 dispensing system)	−45 total hours of direct observations in 5 pharmacies (9 hours of observation per pharmacy) -Follow-up interviews with 20 study participants	NM	-Direct observation: 26 -Interviewsn = 20	-Direct observation: 11 pharmacists + 15 technicians -Interviews: 11 pharmacists+ 9 technicians who participated in direct observation	NM	NM	Content analysis/ NM
Al juffali *et al.,* 2019 [33]	-To explore and compare different stakeholder perspectives regarding safety problems associated with medication supply from community pharmacies in KSA using the HFF Stakeholders include service users, community pharmacists, pharmacy owners, representatives from legal and regulatory authorities.	Community pharmacy	Pharmacy users aged 18 and older were eligible to participate.	Riyad, KSA/NM	Qualitative study	-Professional group recruited purposively, identified through professional and personal networks. -Community pharmacist group recruited using purposive, convenience and snowballing methods -Personal visits to pharmacies and telephone calls were made to invite participants. Telephone interviews were offered only to community pharmacists who could not attend FGs due to job commitments. -A non-profit childcare association providing free weekly parenting courses in community centres was identified, to recruit female attendees who were of various ages and educational backgrounds. -For Male pharmacy user group, an announcement about a support group for caregivers of Alzheimer patients was sent via Twitter from Alzheimer Society account.	-FGs conducted with stakeholders: representatives from legal and regulatory authorities; community pharmacists and pharmacy users. -Individual interviews conducted with community pharmacists only	-Between February and May 2013	−35 individuals -Professionals (n = 8) were Saudis comprised representatives from organisations responsible for regulating pharmacists and pharmacy practice, pharmacy academics +pharmacy owners. -Community pharmacy participants were Egyptian (n = 3) + Yemeni (n = 1).	Professionals FG -Total invited: 15 -Nb of participant (male): 8 (7) -Age years (SD): 40.6 (7.1) -Duration of interaction: 104 minutes Community Pharmacists FG Total invited: 75 Nb of participant (male): 4 (4) Age years (SD): 34.7 (10.6) Duration of interaction: 86 minutes Community Pharmacists Interviews Total invited:5 Nb of participant (male): 4 (4) Age years (SD): 37.7 (13.8) Duration of interaction Mean (SD): 19.2 [3] Female Pharmacy users FGs Total invited:15 Nb of participant (male): 11 (0) Age years (SD): 33.5 (5.8) Duration of interaction: 62 minutes Male Pharmacy users FGs Total invited: 9 Nb of participant (male): 8 (8) Age years (SD): 34 (5.9) Duration of interaction: 89 minutes			Comparative analysis/ HFF
Odukoya and Chui, 2013 [53]	-To understand pharmacy concerns with e-prescribing safety -To identify social aspects of pharmacy staff interaction with e-prescribing that create potential for medication errors that can lead to patient harm.	Community pharmacy	Community pharmacies that processed ≥10 e-prescriptions daily	Wisconsin, USA/7	Systematic qualitative examination of e-prescription processing in community pharmacies	−3 pharmacies recruited through Pharmacy Society of Wisconsin listserv + 4 pharmacies identified by snowball sampling techniques (3 chain pharmacies + 4 independent pharmacies) −14 pharmacists + 16 technicians from 7 community pharmacies were recruited out of 40 pharmacy staff (17 pharmacists + 23 technicians).	-Combination of data collection methods (direct observations, think aloud protocols, and pharmacy team interviews) -Think aloud protocol combined with direct observation (2–5 hours) used to elucidate stepwise processing of an e-prescription, and to identify cognitive/ informational needs of pharmacists and technicians	-Between January and March, 2011	−16 pharmacy staff (7 pharmacists and 9 technicians) took part in Direct Observations and Think Aloud Protocols -Group interviews (1 hour each) were conducted with 2 pharmacists and 2 technicians from each pharmacy/Six interviews were conducted within pharmacies and one interview at a restaurant	Pharmacists Men 6 Women 8 Years of experience: 2–43 Age in Years:25–67 years Pharmacy technicians Men 1 Women 15 Years of experience: 1–30 Age in Years:21–54 years			Human factors engineering (HFE) model called the sociotechnical system (STS) framework
Harvey *et al.,* 2015 [32]	-To examine the activities that take place in community pharmacy dispensing, and to identify the ways in which organisational components either contribute to or reduce safety.	Independent and chain community pharmacy	NM	England/ Pre-EPS2 implementation data: 15 pharmacies (2009–11), and post-EPS2 implementation data: 8 pharmacies (2011)	Qualitative study	-Community pharmacies purposively sampled according to size, geographic location and ownership	-Combination: Ethnographically-informed methods, non-participant observation shadowing, and in-depth interviews	-From December 2009 to September 2011 –	−28 interviews conducted in pre-implementation study and 10 interviews in the post-implementation study and 360 hours of observation	NM	NM	NM	Qualitative approach of socio-technical or ‘socio-material’ paradigms
Lester *et al.,*2019 [58]	-To identify topics within the components of a proposed medication safety framework from the free-text narratives of QRE reports.	Community pharmacy	NM	USA and Puerto Rico/1660	Retrospective, observational study submitted to a single AHRQ-listed PSO	-Pharmacies represented a cohort of 1660 pharmacies in the United States and Puerto Rico −54 pharmacies removed from dataset as QRE reports were in Spanish.	-A structural topic model extracted themes from the free-text narrative component of the report	-From January 1, 2011 to December 31, 2014.	−531,555 reports included in the analysis from 1660 pharmacies.	NA	NA	NA	Models of healthcare quality and safety, including the Systems Engineering Initiative for Patient Safety (SEIPS) Model and the Threat and Error Model
Clabaugh *et al.,* 2021 [51]	-To examine community pharmacists’ perceptions of working conditions at the store and company level and their perceived ability to address patient safety is sues without fear of being disciplined.	Community pharmacy	Respondents had to be at least 18 years of age, currently licensed as a pharmacist in the United States, and either currently work in the community pharmacy setting or have worked in community pharmacy within the past 6 months.	USA/NM	A mixed-methods study used a cross-sectional survey to investigate community pharmacists’ perceptions of company climate, workflow issues, and career satisfaction. A free-response question captured per- -ceptions of safety concerns.	-Facebook pages and through several state pharmacy association e-newsletters. Facebook page coordinators were contacted about posting a recruitment message outlining purpose of study and link to the survey. -Nine state pharmacist associations and 2 pharmacy-related Facebook pages agreed to share survey.	–	–	−1222 participants	–		37.2 years	AHRQ integrative model was utilised as the framework for coding qualitative responses
Kousar, 2019 [49]	-To extend understanding of the nature, outcome and predictors of dispensing errors, and explore community pharmacists experiences following the occurrence of a dispensing error	Community pharmacy	Phase 1: Detailed description of events or circumstances that took place during the build up to the dispensing error Phase 2: Community pharmacists who had made an error and had been subject to an investigation	England	Phase 1: retrospective study Phase 2: Qualitative study Phase 3: Cross-sectional study	Phase 1: -Purposive sample of IRFs -IRFs that contained a well-informed and comprehensive detail of events, circumstances and contributory factors were selected Phase 2 Participants were recruited from the PDA database using an inclusion criteria. .	Phase 1: Retrospective database analysis of incident report forms (IRFs) from database of an indemnity insurance provider with a qualitative analysis on textual description of dispensing errors provided by pharmacists in the IRFs Phase 2: Semi-structured face to face interviews Phase 3: Cross sectional survey	Phase 1: IRFs dated from June 2006 to December 2013 Phase 2: May-July 2016	Phase 1: 706 of around 4000 files detailing 706 dispensing errors (77 retrieved for qualitative analysis	Phase 1: −57% (n = 44/77) female pharmacists and 43% (n = 33/77) males −27% (n = 21/77) worked on an employed basis whereas 73% (n = 56/77) worked as a locum Phase 2: -Seven female and five male participants −50% aged 50–59 years −10 participants worked full time in community pharmacy; two worked on a part time basis			
Family, 2013 [48]	Aim-To: -Investigate the role of MWL in CPs’ and pharmacy students’ performance (in terms of correctly detected DEs) of a final accuracy check of dispensed medicines -Investigate CPs’ and pharmacy students’ MWL during routine pharmacy tasks study how CPs and pharmacy students manage their work if and when they feel mentally under or overloaded -investigate whether expertise in pharmacy tasks impacts on performance on an accuracy checking task and/or the levels of MWL experienced	Community pharmacy and simulated dispensary	Community pharmacists expert and novice and pharmacy students	England/ NA	Mixed Method Study 1: Two accuracy checking experiments were carried out to measure relationship between MWL and DEs. Study 2: Many who took part in the experimental study also agreed to complete a MWL diary. This simply involved rating their MWL at several points during the day. Study 3 Pharmacy students and CPs who took part in experimental study were invited to take part in a qualitative follow-up study. This involved a semi-structured qualitative interview	Study 1: -Fourth year pharmacy students were recruited because they had completed at least one placement in a hospital or community pharmacy prior to taking part and will have completed and passed their final dispensing examinations at the end of the third year - Recruitment of UK CPs was carried out on a ‘top down’ basis. Study 2 -Following participation in experiment 1 or 2 CPs were invited to take part in the MWL diary study. −35 CPs who had taken part in either experiment 1 or 2 expressed an interest in taking part in MWL diary study −26 diaries posted were returned (74% response rate). 14 diaries were also completed by CPs who were taking part in the semi-structured interview resulting in a total of 40 MWL diaries being collected. Study 3: Semi-structured interviews were carried out mid-way through study period for experiments 1 and 2 for both pharmacy student and CP participants	Study 1 used questionnaire for data collection Study 2&3: Qualitative phase using semi-structured interviews + diary fields	Study 1: Data collected over several months in 2011–2012. Study 2: Study 3: Qualitative study with pharmacy students on 16th September 2011 and with community pharmacists on 24th February 2012	Study 1: 104 CPs and 93 pharmacy students Study 2: 40 MWL diaries being collected. Study 3: −15 pharmacy students were selected based on amount of pharmacy experience and to reflect a range of ages and to ensure an even representation of both sexes. −15 CPs were selected for interview based on pharmacy experiencetype of pharmacy age. One CP withdrew their expression of interest before interview took place and decision was taken not to replace this participant as 14 CPs was a sufficiently large sample for this study -Only half of transcripts from interviews were jointly analysed (8 CPs + 8 students)	***Study 1:*** Low WM load group CP: 26 Student 26 Male Gender CP:10 Student:2 Study 1: High WM load group CP: 26 Student 26 Male Gender CP:14 Student:6 ***Study 1:*** Low WM load group-Age CP: 11.7 years Student 21.3 years ***Study 1:*** High WM load group-Age CP: 39.15 Student: 22.12 years ***Study 2:*** Male: 19 Female: 21 Mean Age in years (range): 39.4 (24–61) years ***Study 3:*** CPs: Male: 4 Female:4 Age in years range: 24–67 years Pharmacy Students: Male: 3 Female: 5 Age in years range: 21–23 years			
Wang *et al.,* 2024 [57]	-To analyse HDC opinions on complaints against pharmacists, as sourced from the HDC website, to provide compre- hensive insights into the characteristics and risk domains relating to these complaints.	Community/retail pharmacy [35]/ Tertiary hospital outpatient clinic and community pharmacy [1]/ Pharmacy and medical center [1]	NM	New Zealand/ 37	Retrospective, qualitative study	All narrative HDC full opinions involving pharmacists	Employing a content analysis method utilising nationwide regulatory database of the HDC as the primary data source.	All narrative HDC full opinions involving pharmacists published from 1 January 2004 to 31 December 2022 were collected Frequent checks were undertaken to ensure inclusion of delayed published decisions until 01 March 2023.	A total of 37 HDC opinions were retrieved through the database search.	n = 48 pharmacists Female: 22 (45.83%) Male: 25 (52.08%)			
Odukoya and Chui, 2012 [52]	-To explore pharmacy staff perceptions of positive and negative aspects of e-prescribing in retail pharmacies and to understand how e-prescribing design facilitates or hinders efﬁcient and safe processing of prescriptions.	Retail pharmacies	-Be a retail pharmacy and not a hospital pharmacy (only 1 pharmacy recruited per corporation) -process a minimum of 10 e-Rx daily -process e- Rx from electronic order (vs. immediately printing e-prescription to paper & handling prescription as a paper Rx).	United States/7	Qualitative	-Pharmacists initially recruited through PSW Fast Facts listserv, a weekly electronic newsletter distributed to all PSW members via email. -A follow-up invitation was sent to pharmacists on PSW listserv 1 month after original invitation in order to recruit more pharmacists. -A targeted snowball sampling was then used to recruit more pharmacists.	-Qualitative data collected using: -direct observations and think aloud protocols Observation: duration ranged from 2 to 5 hours depending on frequency of receiving e-Rxs. Think aloud: With every step in dispensing process, participants were asked to verbally state what they were thinking about, what information they needed to fulﬁl each step, what questions they had and how they would proceed to next step.	Between January and February 2011	7 pharmacies (3 chain+4 independent)	NM			Sociotechnical system (STS) theory
Jones *et al.,* 2018 [59]	-To understand reasons why staff choose to violate procedures from a Safety-I and Safety-II perspective	Community Pharmacies	-NM	England and Wales/20	Qualitative	-Purposive Sampling −20 participants identified through local pharmacy professional networks −2 participants recruited via social media −2 participants following recommendations from participants that had been interviewed	-Qualitative data using one to one semi-structured interviews	NM	24 participants	pharmacists (n = 13) pharmacy technicians (n = 2), non-registered accuracy checking assistants (n = 3) dispensing assistants (n = 6)			Critical Incident Technique + Template analysis
Whitaker *et al.,* 2024 [55]	-To establish the process of handing off e-prescription data to pharmacies and identify potential sources of errors.	Retail community pharmacies	-Working in community pharmacy +at least 1 year of experience dispensing medication e-prescription	USA/5 independent pharmacies 4 chain pharmacies 4 health system pharmacies, 3 long-term care pharmacies	Qualitative	-Recruitment fliers distributed to University of Wisconsin School of Pharmacy’s Pharmacy Practice Enhancement and Action Research Link Network, the University of Michigan College of Pharmacy Preceptor Network, and the University of Minnesota Pharmacy Practice Based Research Network.	-Qualitative using remote semi-structured interviews	Between January- April 2023	15 participants	14 Pharmacists 1 Pharmacy technician			Systems Engineering Initiative for Patient Safety

AHRQ: Agency for Healthcare Research and Quality CP: Community Pharmacist DE: Dispensing Error Electronic Prescription Service Release Two FG: Focus Group HDC: Health and Disability Commissioner HFF: Human Factors Framework Hr: hour KSA: Kingdom of Saudi Arabia Manchester Patient Safety Framework (MaPSaF) MWL: Mental Workload NA: Not applicable NB: Number NM: not mentioned PDA: Pharmacists’ Defence Association PQC: Pharmacy Quality Commitment PSO: Pharmacy Quality Commitment program PSW: Pharmacy Society of Wisconsin PEER: Pharmacy and Provider e-prescribing Experience Reporting Portal QRE: Quality-related Event Rx: Prescription USA: United States of America

### Findings: Reporting outcomes, synthesising translations and developing themes and sub-themes

**Fig 2:**
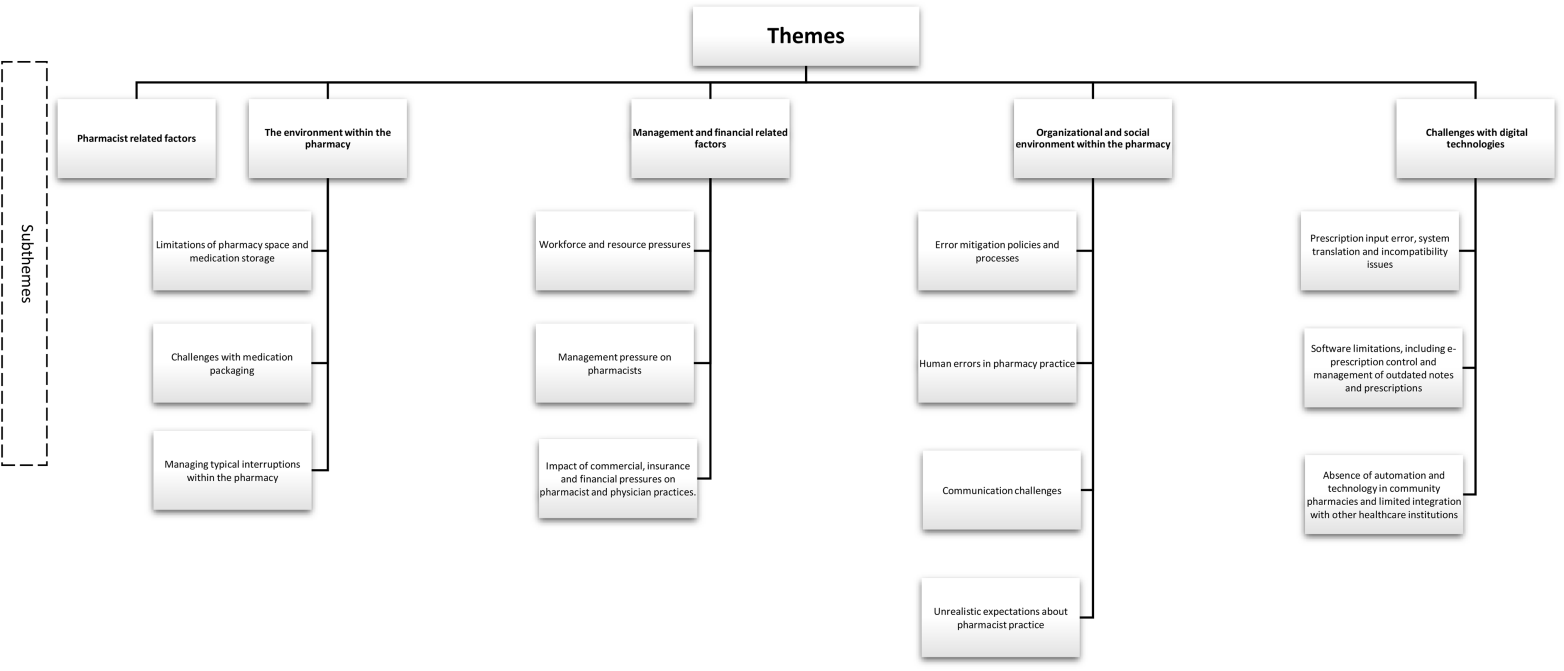
Developed themes and subthemes for factors and or causes of medication errors in community pharmacies.

Five third-order constructs (termed ‘themes’) were developed in relation to the factors and/or causes of medication errors in community pharmacies (Fig 2). Each theme is outlined in a separate table and includes:

1. Pharmacist-related factors (Table 3)
2. The environment within the pharmacy (Table 4)
3. Management and financial related factors (Table 5)
4. Organisational and social environment within the pharmacy (Table 6)
5. Challenges with digital technologies (Table 7)

**Table 3:** Qualitative Data Synthesis. Theme 1. Pharmacist-related factors.

Synthesised themes (third order constructs)	Sub-themes	Second order constructs: the authors interpretations of the original findings	First Order constructs: examples of direct quotations from the participants of the study
Pharmacist-related factors, i.e.,: individual characteristics	Pharmacist competence and experience	Insufficient knowledge, skill, or experience	*“…a major reason for both dispensing errors was that pharmacy staff were unaware at the time of dispensing that both Recormon and tacrolimus came in different strengths.” (HDC09-Provider/ Pharmacist)* [57]
		A city where the medication is uncommon used	*“My pharmacy is in [a city] and we have a large number of diabetic patients. I believe that it is reasonable to expect that the pharmacists at [the pharmacy] were unaware of the existence of Humulin 70/30.” (HDC01-Advisor/Pharmacist)* [57]
		Inexperienced practitioners	*“Investigation into why the order (Mrs A’s repeat* *prescription) was not processed when it was initially received. This was believed to be due to new staff being on duty, and was an oversight.” (HDC08-HDC)* [57]
		Advertising	*“Unfortunately [the pharmacy] was unaware of [Mr B’s] relative lack of experience, which, if known, could have prompted the need for [Mr B] to read [the pharmacy’s] SOPs. The employment agencies concerned have been spoken with regarding this lack of information, as it is they who are advertising these pharmacists as being fit for purpose, and the agencies have a professional responsibility to their clients to supply competent locum pharmacists.”(HDC13- Advisor/Pharmacist)* [57]
		New role	*“…Ms C had been working at the pharmacy for only 10 days when Ms A’s script was dispensed, which would have made her more reliant than usual on previous script history for unusual scripts.” (HDC26-Advisor/Pharmacist)* [57]
		Uncommonly used medication	*“I would offer the explanation that we had never had a prescription for the short-acting Morphine tablets in the 13 y since this pharmacy was established.”(HDC04-Employer/Pharmacist)* [57]
		Not reported by original authors	*“We have many cases … they do not know anything, sometimes they do not even know BID [to be taken two times a day].” (Professional group-2)* [33]

**Table 4:** Qualitative Data Synthesis. Theme 2. The environment within the pharmacy.

Synthesised themes (third order constructs)	Sub-themes	Second order constructs: the authors interpretations of the original findings	First Order constructs: examples of direct quotations from the participants of the study
The environment within the pharmacy	Limitations of pharmacy space and medication storage	Work design	*“If you’ve got [...] [a] layout [in] which you’re literally right behind the sales counter you can eavesdrop on pretty much any conversation, if you’ve got somebody at the far end of the shop ringing a bell every time [they’re selling] a bottle of Calpol, then you’re less able to intervene” [Pharmacy owner, Group 10]* [31]
			*“The “goldfish bowl” dispensary is not a good thing” [Locum pharmacist, Group 9]* [31]
			*“You’ve got to have a tidy dispensary or things do get muddled up and then when you’re working fast you’re just leaping around, grabbing things off the shelf, and unless things have been put in the correct place it’s so easy [to make a mistake]” [Locum pharmacist, Group 9]* [31]
		Poorly configured dispensary setup	*“…while there is a spacious waiting area, the dispensary is set up in such a way that customers can stand right in front of the work bench and cause disruptions.” (HDC24-Provider/Pharmacy technician)”* [57]
		Changing in shelving systems	*“In my view, even if the change in the position of the 2 medications was minor, the change in premises, and the adjustment to a new system, may have added to the pressures on the day.” (HDC32-HDC)* [57]
		Not reported by original authors	“It is a matter of saving electricity just like groceries. At night, they disconnect the refrigerator containing milk to save electricity, and when they come back in the morning, they turn on the electricity.” (Male pharmacy user group-4)” [33]
		The proximity of storage areas	“I also note that subsequently Ms B determined that it was unwise to have 2 medications with similar names stored next to each other…” (HDC34-HDC) [57]
	Challenges with medication packaging	Regular changes to drug funding	*“However, it should be pointed out that PHARMAC, the medicines funding authority, does make regular changes to the schedule of funded medicines. This often leads unexpected changes in the appearance and nature of various medicines as different manufacturers are subsidized for the same drug formulation. This situation adds an extra element of risk in today’s dispensing environment, and is an area where pharmacists must be eternally vigilant.”(HDC10- Advisor/Pharmacist)* [57]
		Regulator enforcing standards	*“We’ve been calling for [manufacturers to change] the packaging and colour [coding] and all the rest of it, we’ve been calling for this for years, and nothing’s happening because [...] no individual body has got sufficient clout, so it needs to go to one central body to actually be able to say “Enough is enough” [Locum pharmacist, group 9]* [31]
	Managing typical interruptions within the pharmacy	Not reported by original authors	*“the error reports haven’t been analysed in detail, one pattern that the pharmacist has noticed is that fewer errors are occurring during an early morning session (starting at 7 am) that was recently started in order to deal with the workload. Could this be due to there being fewer distractions (e.g., phones ringing) during this session?” [Pharmacy 4, Field note from site visit]* [56]
		Interruptions	*“Sometimes when you are doing e-prescriptions you have to stop if someone comes to the window or they ask for something else, you know… if I got half way through the prescription and checking stuff. I won’t process it. I will just exit out of it and have to start all over”* [53]
		Work Design	*“They use being “salaried exempt” as a way to get free work done from the pharmacists. We are expected to go to work meetings and be on conference calls on our days off unpaid. We are made to rush and do the job of a pharmacist and tech to keep the pharmacy afloat from understaffing. The open environment, being constantly distracted from patients interrupting us (open floor plan), and the phones ringing off the hook with not enough bodies to answer the phone leads to a truly stressful environment that makes it difficult to safely multi-task. More mistakes are being made.”* [51]
		Distractions or interruptions	*“While I was in the process of reading the label and when I would usually undertake checking the contents I became distracted when [Ms C] was asking me a question about how she had written the label.” (HDC18-Provider/ Pharmacist)* [57]
		Not reported by original authors	*“I was being interrupted by patients who would reach over and pick stuff [up]…it was awful and I remember the medication that I made the dispensing error with because I remembered the patient. I held up the box of Metformin to [them] and I said, have you had this before, and [they] said yes. It was 500mg and it should have been 850mg”. (P10, Pharmacist, Large Chain)* [59]
		Customer demands	*“I’ve got an open plan pharmacy, and I’ve got people right over the top and talk about interruptions. [...] It takes your whole attention away. [...] The patient can be the biggest distraction you have ‘[Pharmacy owner, Group 10]* [31]
		Attitudes toward safety	*“Viewing television seems to be part of work culture. From about 9.15 to noon, the staff was viewing television whilst dispensing medicines. During the television viewing period, attention of the staff was very distracted. JH recorded: 9.18. There is a television playing on the dispensing counter. At a later time (time not recorded), JH added: There is constant attention on the TV. At one point a customer was left waiting as Counter Assistant (CA) was occupied watching TV. She (CA) has been reminded that a customer was waiting. The television was firmly stationed on the front counter and appeared to be part of the pharmacy’s hardware. To confirm this, a researcher collecting data for a different work stream of this project reported watching television (during his lunch break) at this pharmacy”* [32]

**Table 5:** Qualitative Data Synthesis. Theme 3. Management and financial related factors.

Synthesised themes (third order constructs)	Sub-themes	Second order constructs: the authors interpretations of the original findings	*First Order constructs: examples of direct quotations from the participants of the study*
**Management and financial related factors**	Workforce and resource pressures	Work design	*“Not enough help! Quality help! Takes a long time to train someone and then they leave for better pay and less stress”* [51]
			*“We can’t staff adequately because we can’t get paid adequately.”* [51]
			*“Giving pharmacists a 30 minutes lunch break or 1 hour for pregnant moms to pump and eat can make a huge impact positively in work conditions. Pregnant moms not able to sit or drink water working for more than 12 hours can cause a serious negatively health impact to which I am concerned. When I was pregnant with my first child. I wasn’t able to eat or use the bathroom because I am constantly interrupted with immunizations. i.e., influenza shots, doctor’s calls, patient questions, telephones, technicians needing help, clerk not knowing what to do. The list goes on. Having a chair in every pharmacy for pregnant moms will surely help the mom with her health and making sure her health is taken care of. My biggest concern is making medications error from over working and not taking breaks due to fear of falling behind. Even worse, losing a baby from constantly standing and not able to use the bathroom which lead to UTI which in return does harm To the baby and the mom. The work conditions in community pharmacy is very harsh and dangerous to work in. We cannot afford to make medications error and pharmacists need to eat We are not robots and I need to eat for my growing baby.”* [51]
		Rural location	*“[Mr B] said the pharmacy was ‘extremely busy’ and he* *‘lamented the difficulties in attracting pharmacists to our rural pharmacy’. This is by no means an excuse, but is a very important context for the error.” (HDC09-Advisor/ Pharmacist)* [57]
		Inadequate staff support in the dispensary	*“While the pharmacy had in place appropriate SOPs, it had not ensured that there was a sufficient number of qualified staff supporting Ms B in the dispensary on that day.” (HDC22-HDC)* [57]
		Not reported by original authors	*“…it was awful. So we shut the shop up, I rang the area manager, she was really supportive…and when she found out I shut the store, after two hours we had a second pharmacist come along, taken from another store. But it took us shutting the shop to get that help.” (P10, Pharmacist, Large Chain)* [59]
		Expectations	*“our volume is such that staff want to cut corners to save time (like staging prescriptions).”* [51]
		Not reported by original authors	*“Obviously now that I’ve thought about this [I wouldn’t do it again]…at the time it would seem we [needed] to keep things moving…there’s loads of people around; there’s loads of noise; kid’s not well; mum’s upset as well because the kid’s not well; all you can see is just stressed people, and by doing this quickly you can make [things] better.”* [59]
		People and their approaches to work	*“ 10.58. Locum is left alone at pharmacy. Customer is at the counter, and so he goes to see to them. Phone is ringing but locum is busy. He has left the FP10 (prescription he is processing). The phone has stopped ringing just before he finished serving the customer. He goes back to dispensing the FP10. It is now 11.00.* *11.00. A customer is at the counter. Locum stops dispensing to see to customer. Customer hands over FP10 but keeps chatting to the locum despite him [locum] showing his desperation to go. Customer now leaves (for a call-back). Another customer immediately walks in and asks for something. This has taken locum to a different area (looking through records). The dispensing still waits. 11.04. A new customer walks in with two FP10s. There are now a few customers waiting and only the locum. He is trying not to get stressed.* *11.08. Phone rings again. [Locum runs by me (JH) and says I can’t split myself into two]. Locum now stressed.* *11.09. Phone rings again. There are about five customers waiting. Problem is exacerbated by each customer wanting a chat and asking questions. Locum very patient and tries to give each customer all the time they demand.* *11.15. There now three customers but other items on the FP10s are dispensed. Call-back lady at 11.00 now comes to collect. It is 11.15.* *Dispenser now arrives from breakfast break. Work is now shared but there is now a queue of FP10s. Problem with medication as they may not have some in stock. Customer from 11.15 comes back at 11.35 only to be told that item (medicine) is not in stock.”* [32]
		Group behaviour	*“My position in a grocery store pharmacy has me working all phases of production and then I still have to ring out the customer and all of their groceries. I’m not above it, I simply don’t have the time.”* [51]
		Understaffing	*“There was considerable work pressure on the staff because there were unusually high workloads and an unexpected staff shortage.” (HDC18 Provider/Pharmacist)* [57]
		Not reported by original authors	*“[The prescription] had already been counted, it had gone out, after it was checked, clinical checked, the patient had had it many times before…So, I squiggled on it and [filed it]. It was the end of the day, we were very short staffed…it was towards the end of the week as well, so [the prescription] wouldn’t have come back until the next week and then with the amount of prescriptions that we get in, these aren’t [followed up]…so it’s losing items.” (P12, Dispenser, Large Chain)* [59]
		Sole pharmacist on duty	*“Mr B also informed me that being the only pharmacist on duty at the time Ms A presented her prescription, it was likely that he could have been interrupted by phone calls and other customers walking into the pharmacy.” (HDC05-Provider/Pharmacist)* [57]
		Cooperation issues	*“However, on the day of the error one technician was* *covering a retail person who was sick which may have led to the problem.” (HDC16-Advisor/Pharmacist)* [57]
		Not reported by original authors	*“We often, especially when we’re in a bad place pop, check and deliver without clinicals on scripts and what the pharmacist will do at the end of the day is take a batch and then clinical them retrospectively… Because of the set up we’ve got with the two ACTs upstairs but only one pharmacist downstairs in a busy walk-in, it happens more than you’d like it to happen. We have got into a bit of a better routine [now]… but again it goes back to when we are in a really bad place your rules go out the window.“ (P2, Dispenser, Large Chain)* [59]
		Not reported by original authors	*“The support staff went into a side room with us for the workshop, but the pharmacist remained outside, working at the dispensary counter. During the workshop, the pharmacist called to the support staff to hurry up as they needed to get back to work.” [Pharmacy 7, Field note from first workshop]* [56]
		Involvement	*“People having their own training programme [for example], that’s [a lot of] time input, and [in] an ideal world that’s great, but pharmacy is really skinny with staff, we really work every minute. Like now, we’re itching to do [medicine] trays right now.” [Pharmacy 5, Workshop 2]* [56]
		After work time	*“He told HDC that he did not have the opportunity to query the increased prescription with the Dr D, as it was too late in the day.” (HDC28-Provider/Pharmacist)* [57]
		Pharmacist working hours	*“He has long working hours and that leads many pharmacists to not refresh their information. [by attending workshops for continuing their education], …there is no role for the Ministry to update your information. For example, the pharmacist (Community pharmacy group-4) graduated in 1986 and necessarily many improvements[updates] have taken place since that time.” (Community pharmacy group-2)* [33]
			*“Often, pharmacists find themselves compelled to work in place of their colleagues for extra hours. For example, the average working hours are 15 or 17 hours [a day]. Seventeen hours is a [long time]. The maximum hours we have are 15 hours for certain periods in a month.” (Community pharmacy group-1)* [33]
		Engagement with technologies	*“11.11. While staff uses computer, another staff drums her fingers on the counter waiting to use the terminal. She (first staff) jokes “No pressure” while a third staff waits.”* [32]
		Quality emphasis	*“Pharmacies used to be able to afford safety features and automation but even that cannot longer be afforded”* [51]
		Low salaries	*“I don’t expect anything from the pharmacist, because all pharmacists are frustrated and this is due to their low salaries and the nature of their work. In a pharmacy, I noticed a pharmacist working in the middle of a hot day on Friday and he used to walk three or four kilometres.” (Male pharmacy user group −5)* [33]
		Budgetary constraints	*“Staff is the single most expensive item in any business, whether it be pharmacy or anywhere else, and all the firms [...] are under pressure from their shareholders to make more profit, and the easiest and quickest way to do that is not increasing turnover, it’s to cut down the staff [Locum pharmacist, Group 9]”* [31]
	Management pressure on pharmacists	The role of Pharmacy owners and managers	*“Problems between you and the owner arise; he [the owner] asks what happened … [you] pay a penalty, close the pharmacy and your license is suspended. These problems face us and affect our work.” (Community pharmacy group-4)* [33]
		Organisational structure	*“Constant emails, texts, calls and visits from pharmacy DM regardless of how hard you work”* [51]
			*“My boss isn’t even a pharmacist! Tells me to fill opiates as cash to keep complaints down...this increases the opiate crisis and diminishes my personal values.”* [51]
		Leadership	*“Corporate puts too much emphasis on scripts filled by certain times and just getting refills in the system and discipline pharmacists who don’t meet the criteria.”* [51]
		Management structures	*“It is conflicting, because you care about your patients and you think it’s not about targets. Like the old lady that came in and she was talking about her painkillers when she has a headache. I noted that down, I could use that as an MUR in the sense that I did sort of tell her that she’s not supposed to be taking them all the time. It’s good that you can use it, because it’s exactly what it is, you are telling the patient how to use them and advising them about it. Those are the little things that I have to concentrate on and pick up on to meet my targets.”* [32]
		Quality emphasis	*“Everything is based on company profits (i.e., scripts to budget, tech hours to budget, pharmacist hours to budget) instead of patient safety.”* [51]
			*“Would love to do med recs/mans with my patients but I have absolutely no time for such things because metrics are watched so closely”* [51]
		Leadership	*“If I get a complaint I could be written up even if I had a valid reason that they had to wait I am always in the wrong and must call to apologize even if they were wrong the patient is never wrong and my professional judgment on not filling or refusing a patient is constantly being questioned and must be defended against a patient who doesn’t know anything about my job or the logistics of pharmacy billing dispensing and law.”* [32]
		Work design	*“Corporate not backing pharmacist professional judgement when we decide not to fill a prescription and encouraging us to fill it anyway just for patient satisfaction”* [51]
	Impact of commercial, insurance and financial pressures on pharmacist and physician practices	Healthcare system role in commercialism	*“In our country, the pharmacist gives you the medication that is suitable to him or the medication for which he receives a commission, you go to the pharmacist you say you have a headache he gives you Fevadol instead of Panadol [generic substitute] for example he gives you the medication that suits you, who he is an agent for it or gives him a commission for. Even in clinics, the representative of the company comes to the physician and gives him the new medications with tickets and gifts and the physician prescribes the medications.” (Male pharmacy user group-5)* [33]
		Physician prescribing behaviour	*“The medication is prescribed due to a commission, and this has resulted in a loss of confidence between us and physicians.” (Male pharmacy user group-7)* [33]
			*“The quality of the physician usually they come with very low salaries but depending on the commissioning they get from the companies and still they have the commission [they overprescribe medication] we see the kind of prescription which is very weak [with many mistakes] even our pharmacists discover these mistakes it happens with me a lot so due to this kind of this low educated physicians.” (Professional group-2)* [33]
		Generic substitution	*“I comment on availability issues. Some of it [is] truly shortages, and some of it is [not]. Unfortunately, this is a commercial business. I know some pharmacies will not introduce a product without getting fees or getting huge bonuses from the company. The company will not be able to sell it that is again with the law how far you can enforce the law on community pharmacy?” (Professional group −4* [33]
			*“He sometimes tells you about an alternative if one drug is expensive. I hear him saying, ‘its price is seventy, but there is an alternative that is only forty’.” (Female pharmacy user group −2)* [33]
			*“If you go to a pharmacist and you say you have a mild or minor ailment and ask for a prescription, you have two products [options] one product will fit you, but that does not have a bonus, the other product has a bonus.” (Professional group-4)* [33]
		Patient counselling	*“The pharmacists’ required trait is honesty. He should be honest when giving an opinion or at least not give advice if his advice is for commercial purposes. His positive role is absent here.” (Male pharmacy user group-3)* [33]
			*“My problem is always that when I go, they give me the best and the latest on the market, that is to say, they do not give me the one appropriate for me. The problem is that they do not try to learn whether it is appropriate or not.” (Female pharmacy user group −1)* [33]
		Healthcare system role in commercialism/Physician prescribing behaviour	*“Listen, the biggest problem of the medical insurance is that the doctors prescribe medicines they want to sell or will gain benefit from. The most important purpose of most pharmaceutical companies, not all, nowadays, is the sales nothing else, therefore they influence some doctors to prescribe their medicines regardless the patient needs it or not... Ok. This is the main problem of the insurance, because the patient does not pay high amount, therefore the prescription contains medicines that are over the patient’s need, prescribed just to be sold, no more” (Community pharmacy Interview-3)* [33]
		Financial dependencies	*“We’re gonna be [...] competing for money [from the Primary Care Trust] cos they’re gonna have seventy five percent of the budget and [...] we’re gonna want their services, so it might prevent us from being honest [with them] about our mistakes and errors” [Employed pharmacist, Group 4]* [31]
		Profitability vs safety	*“It’s very hard to make it work when you’ve got [...] whistle-blowers in two different sorts of organisation. Like non-profit organisations like nurses and GPs, and pharmacists in profit organisations, we’re supposed to whistle-blow on GPs if we see bad prescribing, but to the detriment of our business?” [Pharmacist, Group 8]* [31]
		Reimbursement	*“PBMs will be the downfall of pharmacy. Customers are having choice removed from them due to PBMs. Patients can no longer use the pharmacy of their choice and are having to go chains that are more understaffed than mine with staff that they don’t trust Some are choosing to spend more money to stay at a preferred location.”* [51]
			*“Everything is about money and not about health or wellness. This leads to the company trying to make a profit at any cost. With decreased reimbursement from insurance and PBM companies, the pharmacies solution is to cost control which leads to less hours allotted to the stores and cut payroll with increased pressure to perform more mentally demanding clinical tasks while juggling increased dispensing amounts per pharmacist This leads to mistakes and ultimately the patient’s suffer. Patients don’t understand this shift to clinical work in a community setting which leads to confusion and anger on both sides.”* [51]

**Table 6:** Qualitative Data Synthesis. Theme 4. Organisational and social environment within the pharmacy.

Synthesised themes (third order constructs)	Sub-themes	Second order constructs: the authors interpretations of the original findings	*First Order constructs: examples of direct quotations from the participants of the study*
**Organisational and social environment within the pharmacy**	Error mitigation policies and processes	Outdated SOPs	*“However, I note that the pharmacy had preferred practices that it encouraged pharmacists to employ relating to its checking procedures that were not included in the SOPs at the time of events. I am critical of the pharmacy that its SOPs were not up to date to reflect its current practices.” (HDC31-HDC)* [57]
		Inadequate dispensing and checking procedures	*“It appears from the reports and documentation provided that the systems and SOPs in place were not of an adequate level to provide a safe working environment for the pharmacists employed by [the Pharmacy], or to provide a safe service to the clients of this pharmacy, for methadone dispensing services.” (HDC11-Advisor/Pharmacist)* [57]
		Inadequate packaging policies	*“…a couple of contributing factors led to the error. One was … and another was the pharmacy’s decision to change its clear plastic cups to paper cups.” (HDC33-Provider/Pharmacist)* [57]
		Inadequate controlled drug policies	*“…without the clause on controlled drug storage, the SOP would be considered incomplete and therefore inadequate.” (HDC36-Advisor/ Pharmacist)* [57]
		Not reported by original authors	*“When it’s emergency situations, when everything’s open, great, but what do you do when they’re not open? When you don’t have access to the […] resources that […] are documented in your procedures, then you have to think of other routes to get things done …there will be cases when something isn’t covered by a procedure. [For example], it could be your driver’s called in sick and you have to go and do your deliveries, what would you do?” (P6, Pharmacist, Independent)* [59]
		Inadequate SOP development processes	*“It would be appropriate for staff involved in providing services such as methadone dispensing to be involved in the maintenance and development of SOPs to be used in that pharmacy. [The Pharmacy] did not seek* *input from the pharmacists involved in providing a significant part of the operations of [the Pharmacy] in developing their SOPs.” (HDC11-Advisor/Pharmacist)* [57]
		Inadequate incident management	*“…the pharmacy did not have a process for dealing with broken packs, nor did it require staff to report dispensing incidents to management.” (HDC22-Advisor/Pharmacist)* [57]
		Individuality and professional autonomy	*“I know other pharmacists who [...] definitely stick to the rules, no matter what, and are not gonna bend ‘em. Then some people who kind of just squeeze past them. So it does depend on the person” [Pharmacist, Group 2]* [31]
		Attitudes toward safety	*“There are color baskets red, white and blue which are supposed to be used to organized dispensing, but I have just been informed that this procedure is not usually followed.”* [32]
		Noncompliance with the pharmacy’s SOPs or relevant standards	*“By failing to ensure that she dispensed the correct medication to Ms A, Ms C failed to comply with the relevant professional standards outlined above, and did not adhere to the pharmacy’s SOPs.” (HDC14-HDC)* [57]
		A culture of nonadherence to SOPs at the pharmacy	*“In my view, the number of errors at the pharmacy, and the fact that these have been made by more than one staff member, indicate a systemic problem with regard to staff failing to follow the pharmacy’s SOPs.” (HDC14-HDC)* [57]
		Not reported by original authors	*“I think you see one person [going against a procedure] you think oh that’s alright.” (P12, Dispenser, Large Chain)* *“[I violate] if other colleagues do the same thing, and they’re just like, that’s fine; we always do it like this…”(P3, Dispenser, Large Chain)* [59] *“I tend to just ask the dispensers…how they work, their daily routine, what goes on. It could be something as stupid as how do you bag up [medication]…just things like that…I would assume that they’re following the SOPs…there’s no way I could read a whole folder [of SOPs] in the space of five minutes.” (P11, Locum Pharmacist)* [59]
		Group norms	*“[We] were taught, certainly in my era, at College, you did not make mistakes, you covered them up, that was the history. I had a boss who I could have killed because he did make mistakes but he refused to admit it. [...] [I] went in to the dispenser and said “Look, ignore him, we all make mistakes, we check each other” [Locum pharmacist, Group 9]* [31]
		Not reported by original authors	*“They said, ‘I don’t think it’s something we need to do because we’ve done it in the past…[it] creates a lot of paperwork and nothing went wrong’…So, I deviated but I was kind of coerced into doing it…I was very uncomfortable but I felt I didn’t have a choice…they were the area manager, who else was I supposed to tell?” (P10, Pharmacist, Large Chain)* [59]
		Credibility and practicality	*“If you’ve got a patient who’s at risk and if you’re doing something or not doing something, then I would ignore [the protocol] and do what I thought was the professional way, would be the best thing to do” [Pre-registration pharmacist, Group 5]* [31]
		Doing what’s best for the patient	*“I sometimes think they’re not very helpful for patients. If you’ve got a protocol and you’ve got to do it a certain way, but then you can’t, say a checking one [...] do you break it for the benefit of the patient, even though you know you shouldn’t? “ [Pharmacist, Group 2]* [31]
		Not reported by original authors	*“I dispense everything; I am a pharmacist regardless of the laws, when you have a patient in front of you needing to be treated it would be difficult especially if the patient is poor and needs assistance you do not help him; for humanity” (Community pharmacy group-4)* [33]
		Not reported by original authors	*“We are not only pharmacists, but also marketers. … I know why he wants the Liponex, whether he wants to sell or take four or five tablets … The same happens with the psychological medication … we may dispense it…Solving the problem of insomnia may not be that he cannot sleep, it may be depression, so we give him antidepressants like Liponex. A week prior to marriage anti-depressants may be needed, and we give Sirolex either for a man or a woman.” (Community pharmacy group-3)* [33]
		Not reported by original authors	*“I think as a pharmacist you’re supposed to use your professional judgment, and sometimes the SOPs prevent you from doing that, [because] if you followed them to the letter then you might not always have the patients’ best interest at heart. And so they can be a bit of a conflict sometimes.” (P9, Pharmacist, Large Chain)* [59]
		Not reported by original authors	*“[Loans happen] all the time…it almost feels like the surgeries won’t put the patients first, whereas we have to…[the surgery say the patient] should have [ordered their medication] sooner…[but] that’s not very helpful to anybody…I know that [the GP is] busy but that means I’m going to have to give [the patient medication].” (P9, Pharmacist, Large Chain)* [59]
		Not reported by original authors	*“Even if it’s a medicine that won’t affect them [if they miss a dose]…you still want to make sure that [the patient takes] it and they don’t miss a day out. So, the benefits are mainly to the patient more than anyone [when loaning medication].” (P11, Locum Pharmacist)* [59]
		Not reported by original authors	*“If somebody’s on their death bed is it right to withdraw treatment on a [legal] technicality? There’s consequences of [following procedures] because we’ve not put the patient first, which is our first and foremost concern…The judgements that we make are serious judgement calls…that could jeopardise our careers and everything we’ve worked for”* *(P6, Pharmacist, Independent Pharmacy)* [59]
		Following the law vs meeting demands	*“You get somebody coming back and say, [...] “I was owed five bendrofluazide tablets.” And there’s absolutely no record [...], somebody’s [must have] made a mistake at this end [...], so [...] you give it out. Now, technically that’s going against the law [...] [but] at the end of the day there’s a little bit of a grey area” [Locum pharmacist, Group 6].* [31]
		Not reported by original authors	*“[The prescription] had already been counted, it had gone out, after it was checked, clinical checked, the patient had had it many times before…So, I squiggled on it and [filed it]. It was the end of the day, we were very short staffed…it was towards the end of the week as well, so [the prescription] wouldn’t have come back until the next week and then with the amount of prescriptions that we get in, these aren’t [followed up]…so it’s losing items.” (P12, Dispenser, Large Chain)* [59]
		Involvement	*“[We have to] be clear in our working ethic, clear in our procedures but also actually think about general safety as part of your day to day practice. [For example, you see a box left out]. We’ll question it; why’s this box left out, why’s this cream stood there, why is it not in the cream drawer, why is this here, that kind of thing” [Pharmacy 2, Focus group 2]* [56]
		Approach to governance/Hierarchy and protection	*“The advantage of notifying the Head Office [of a chain] is that they then cascade the information to everybody so that every store can then separate the Xalacom and Xalitan in the fridge so that it doesn’t happen, so you’re actually avoiding the error ever happening” [Locum pharmacist, Group 9]* [31]
			*“I think the protocols that [chains] have as well tend to be stricter [than in independents], and they won’t let you bend from the protocols” [Employed pharmacist, Group 6]* [31]
			*“No, because they’ve got so many branches to cover, they’ve got to put it down as a must do, rather than a, well, we’ll get round it type of thing”* [31]
		Type of pharmacy and its effect on medication safety	*“We talk about the chain of pharmacy we spend a lot of time to train our pharmacists before going to be behind the counter to dispense medication…Chain of pharmacy or chain group it is easy to implement any regulations” (−2)* [33]
			*“[Chain pharmacies] have a policy that we will not violate the law and the patient will find what he wants [in another pharmacy]; this is the mistake of the patient [if he asks for a medication without a prescription as he will not get it from this chain pharmacy]” (Community pharmacy group-1* [33]
			*Street pharmacies [independent pharmacies] have more problems because the responsibility is like a burden on the pharmacist’s shoulders. He becomes a physician. On the other hand, when I am in a place [where] there is a clinic, half of my time is spent on prescriptions from the clinic, and the physician upstairs is doing his duty, writing the medicine that suits the patient and explaining to him the safety of the medicine. The clinic helps me, but when I work in a street pharmacy, I almost work alone, so I have to exert extra effort to explain to patients.” (Community pharmacy Interview-4)* [33]
		Blame culture vs learning culture	*“One of the issues [...] is getting rid of this blame culture. Although we’re trying to instil [that] it doesn’t really matter who did it, [...] let’s move on and learn from it, people are still very hung up on who actually committed the error”[Locum pharmacist, Group 9]* [31]
		Contemplating change	*“I think we fail on writing incidents up. […] I think we’re brilliant with [near misses], but […] incidents that leave the shop, I do think we’re quite poor at.” [Pharmacy 4, Workshop 2]* [56]
		Prioritisation	*“You might just be busy at the time. So if we’ve got a shop full of people you’ve not really got time to [stop], and [reporting a near-miss] might just get forgotten about because you might be occupied with something else.” [Pharmacy 8, Workshop 2]* [56]
		Trust and engagement in governance	*“If your reports are gonna go “Look, this pharmacy’s got this much near misses, ooh, it’s a black-listed pharmacy, this one.” But if they’re gonna [...] recognise that you’ve got these amount of near misses or whatever, this is what you could do, this what you could do to improve. [...] Is it gonna be against you or for you in that respect?” [Pharmacist, Group 8]* [31]
			*“There’s no point in being proactive to a system or management or a body which is itself being reactive and disciplinary, because that defeats the point of you being proactive in the first place [Pharmacy owner, Group 10]* [31]
		Supervision	*“Lots of don’t ask don’t tell attitude that promotes unethical and illegal behaviours and cannot be reported due to fear of job loss.”* [51]
		Not reported by original authors	*“The manager told us that staff were getting better at near-miss reporting following the first workshop. However, I didn’t really get a clear answer as to whether there was now agreement between all members of staff about using the near-miss form. The form is on the counter, so all staff can readily access it.” [Pharmacy 6, Field note from post-workshop visit]* [56]
		Monitoring and audit/Reporting and feedback	*“Newly qualified [...], couple of days in, made a mistake, and I told them and we talked about it, but I didn’t report it to head office, cos I knew that they were devastated [...] I gotta think about this person and they would probably not wanna be a pharmacist any more. [...] My thought [was that] they really learnt from this, and I would be surprised if they did it again [Pharmacist, Group 2]”* [31]
		Inadequate documentation and recording	*“Amending records in this way without identifying that the amendment has been made retrospectively is very poor practice. Furthermore, by making amendments to the records in this way, Mr B removed the record of what actually occurred, which is also unacceptable.” (HDC14-HDC)* [57]
	Human errors in pharmacy practice	Inadequate or inaccurate dispensing and checking	*“I followed the pharmacy’s dispensing procedure except that my final checking failed to disclose that the wrong brand of isosorbide had been included in the blister pack.” (HDC07-Provider/Pharmacist)* [57]
		Inadequate labelling or packaging	*“In this case, it appears that human error or haste on the part of Mr D (or another pharmacy staff member) caused Mrs A’s prescriptions to be bagged incorrectly.” (HDC08-HDC)* [57]
		Inadequate actions on verifying the appropriateness of the medication	*“In my opinion [Mr B] and [Mr C] do not appear to have accurately assessed – the patient details, in particular the patient’s age and patient category – the appropriateness of the medication for the patient.” (HDC03-Advisor/Pharmacist)* [57]
		Patient as final safety barrier	*“I [once] called the name, didn’t ask the address, spent ten minutes counselling [the patient] on how to use an inhaler and [he] came back and said “This is the wrong thing. I was expecting tablets”. [...] That was my mistake, [but] that goes to show you how much he was listening” [Locum pharmacist, Group 1]* [31]
		Forgetting to read carefully	*“Forgetting to read carefully”* [58]
		Attention-related	*“not paying attention when selecting LASA drug, strengths, & form”* [58]
		Unfamiliarity with the patient	*“Mr A was not familiar with the identity of the other consumer or Ms B, and thought that the correct patient went into the consultation room with him.” (HDC27-Provider/Pharmacist)* [57]
		Correct ingredient, wrong strength	*“Dextroamphetamine, which could be filled as Adderall (i.e., mixed amphetamine salts) or Dexedrine (i.e., pure dextroamphetamine).”* [55]
		Correct ingredient and strength, wrong release form	*“Adderall XR versus Adderall immediate release will happen.”* [55]
		Correct medication and strength, wrong form	*“Ophthalmic (eye) versus OTIC (ear) Creams versus ointments Tablets versus capsules”* [55]
		Correct ingredient and release form, wrong strength	*“Verapamil ER 240 versus Verapamil ER 360”* [55]
		Wrong ingredient	*“Hydroxyzine and hydralazine, ropinirole and risperdal, or fluvoxamine and fluoxetine”* [55]
	Communication challenges	Group norms	*“I [once] had an issue with methadone, [...] I did not agree with what the pharmacists had done the previous week and they’d done nothing about it all week when they had time to sort it out and then they didn’t even tell me in advance of me going, I walk in on the Saturday and get stuck with the real issue, do you give it, don’t you give it. And you’re dealing with something that’s quite, you know, can change that person quite a lot, and you’re thinking, “Well, where do you stand?” [Locum pharmacist, Group 3]* [31]
		Knowledge	*“We get no communication [about] what’s happening in [the] other branches […]. We have 20 shops in the company now, but I don’t hear about [the] common errors […] or any incidents, which I think we could learn from.” [Pharmacy 1, Workshop 1]* [56]
		Not reported by original authors	*“There is more potential to break HIPAA [U.S. Health Insurance Portability and Accountability Act, a legislative act that protects patients’ health information]. Because we have a small pharmacy, I’ll be over here [pointing to opposite side of pharmacy] and I’ll say, ‘Hey, can you pull a prescription for [John Doe],’ or whomever the patient may be. I’ll say it across the pharmacy, so the other patients know who the person is. Whereas with the paper prescription, it’s there and you don’t even have to say anything.”* [53]
		Organisational culture	*“I think you tend to get more demoralised staff in a company and more negative on feedback and communication to a well run independent sometimes, because I think they’re sort of all put into a block and they can be boxed if you’re not careful in a company”* [31]
		Relationships	*“It does make it awkward when […] they know that the situation you’re talking about involves them. […] You can […] feel them looking at you and you [think] ‘should I have said this?’” [Pharmacy 4, Workshop 2]* [56]
		Not reported by original authors	*“I can see part of it. If they’re giving you [paper] prescriptions, you know they’ve given you three prescriptions. If they say, ‘Oh, the doctor just sent down an e-prescription for me,’ Okay, well did they send down one prescription or did they send down four prescriptions? Sometimes patients know and sometimes they don’t. Even just for telling them a time of how long it’s going to take, if I can see they have three prescriptions here, in my mind I can do that. Whereas with e-prescriptions I’m not entirely sure how many have come down, so I say, ‘Oh, yeah, it will be ten minutes,’ because I thought it was one prescription, but ‘Oh, wait there are four prescriptions. Okay, it’s going to be 20 minutes now.’* [53]
			*“A lot of times when a patient comes down, they expect the prescription to be ready already because it was sent down electronically. Whereas when they bring it in with a paper prescription, they know that this is where it starts, right there. With an e-prescription it started 20 minutes ago, when the doctor sent it down. ‘I’m here to pick up a prescription.’ That’s how they come in. ‘Well, did you call it in or did the doctor send it?’ ‘The doctor sent it. It was 10 minutes ago.’ …That doesn’t take in to account the fact that three other doctors also sent prescriptions down all at the same time, so we’ve got four people coming down all for prescriptions at the same time, that all think they’re the only one. Then you’ve got four people waiting all of the sudden.”* [53]
		Pharmacist-Patient communication	*“The pharmacist should care about communication with the patient and not get bored questioning the patient. Despite the feeling that the patient does not want to be asked a lot of questions, the pharmacist should do what is best for him ethically. The goal is the patient’s benefit.” (Community pharmacy group-1)* [33]
			*“I did not ask, but he was proactive. Just a little information about the medication and I will be thankful, as he is the specialist. I am sure that some of the medications have red lines [cautions]. Even if they are licensed, I need to be informed about the cautions on them.” (Male pharmacy user group-2)* [33]
			*“I think education (being proactive) is not the role of the pharmacist. It is impossible to explain everything to everyone. If the patient asks, he should answer; if the patient does not ask, it is not the role of the pharmacist to explain.” (Male pharmacy user group-4)* [33]
			*“What I notice is that they take the prescription and put it on the counter, and that is all. They do not even say hello.” (Male pharmacy user group-1)* [33]
		Factors affecting communication exchange	*“I came across someone who didn’t know whether the medication was for constipation or diarrhoea. He said he wanted something for diarrhoea. The matter is that he didn’t want something for diarrhoea; he wanted something to cause diarrhoea. In brief, language has an effect.” (Community pharmacy group-4)* [33]
			*“He gave him many options, maybe his child’s age, I don’t know, but he gave him options and explained [things] to him and gave him more time. I kept waiting. When it was my turn, he said to me, ‘this is the best, so take it.” (Female pharmacy user group −1)* [33]
			*“There is no chair in the reception, in the middle in front of him, a large space so he can put Strepsils and gum. You go abroad; there are chairs for waiting because he knows he will take some of my time to discuss information with the patient before me … no chairs for waiting, and if you wait, don’t expect them to tell you anything.” (Male pharmacy user group-5)* [33]
		Inadequate communication with patients or customers	*“To not have discussed with [Ms A] the dispensing of a different product from the one prescribed would be a severe departure from the accepted practice of keeping the customer fully informed about their medication.” (HDC25-Advisor/Pharmacist)* [57]
		Communication between pharmacists and physicians	*“We also do not know how to communicate with physicians, secondly in order to communicate with him again we have to request his phone number. If I work at a pharmacy, which is far away from the clinic and try calling him my call is divert to an answer machine and they leave on hold the physician does not reply and you start from scratch to call again and stay on hold, you are keen to give the patient the right medication […] I think there is a safety problem with the prescription trying to communicate with him would be impossible.” (Community pharmacy group-1)* [33]
		Not reported by original authors	*“There is another concern in that it doesn’t always print the information of the doctor where they’re currently at. If you have a doctor working a walk-in or an E.R. [emergency room] and you get a prescription from them, it sometimes lists their office phone number. Then if you have a question on it and you try to call the phone number that’s on the e-prescription sometimes they’re like, ‘Oh, well they’re not here today.’ Then you have to call around or they have to bounce you around from person to person until you can find where they actually are practicing. So it makes it harder to track them down.”* [53]
			*“It’s less about ‘I can’t read what this is. What is it?’ but it’s more just, ‘Are you sure this is what you [prescriber] meant?’ So it’s really focusing more on clarification of what they [prescriber] intended, rather than trying to decipher what they actually wrote.”* [53]
	Unrealistic expectations about pharmacist practice	Psychological pressure	*“Mr B told HDC that the relationship between Mr A and himself broke down, and that he felt pressure from Mr A, which he considers may have affected his dispensing decisions.” (HDC28- Provider/Pharmacist)* [57]
		Poor relationship with the patient	*“I acknowledge that there was a breakdown in the relationship between Mr B and Mr A, and that Mr B believes that this affected his dispensing decisions.” (HDC28* *Advisor/Pharmacist)* [57]
		Counterfeit medication as consequence	*“Originally, it is prohibited by the Ministry of Health to dispense antibiotic as a strip and if this is done it would be a violation and in case of not dispensing them in this form, the patient will go to a second, third and fourth pharmacy until he finds what he wants” (Community pharmacy group-1)* [33]
		Customer demands	*“I think we’ve got to get away from the idea that a good pharmacist in the view of the public is one who gets the medication out quickly. [...] They just assume that it’s going to be correct but they don’t rank the actual quality of the dispensing in any of it, they put speed at the top” [Locum pharmacist, Group 9]* [31]
		Quality emphasis	*“Something has to give and when it does, the patients pay the price with prescription errors. It’s not fair to the patients. They deserve a pharmacist who can give their prescriptions the adequate attention needed”* [51]
		People do not understand what is involved in the filling	*“but most of the pressure I feel comes from the patient.* *People do not understand what is involved in the filling/verifying process and think all we do is slap a label on it They don’t understand why it takes so long. This then affects my corporate patient satisfaction score”* [51]
		Expectations	*“Corporate pharmacy has over simplified and belittled pharmacists jobs to the point that the patient (they prefer “customer”) has come to expect instant service. The general public needs to be educated on the complexity and seriousness of this job. READ CAREFULLY. TWICE. We are being swept away from a thought-provoking task of providing medicine to an individual to an overmechanized and automated rubber stamp/conveyor belt assembly line “I’ve been waiting two minutes...isn’t it done yet?” world Re-educating the public is the second greatest concern.* [51]
		Not reported by original authors	*“If you’ve got [customers] just wanting to pay for things, and they’re waiting ages because you’re talking to somebody… it would be a lot quicker if I just sell what people are asking for, and if I don’t ask the questions.” (P19, Locum Dispenser, Large Chain)* [59]

**Table 7:** Qualitative Data Synthesis. Theme 5. Challenges with digital technologies.

Synthesised themes (third order constructs)	Sub-themes	Second order constructs: the authors interpretations of the original findings	*First Order constructs: examples of direct quotations from the participants of the study*
Challenges with digital technologies	Prescription input error, system translation and incompatibility issues	Incorrect calculation or entry of information	*“Pharmacist: “The main impression that I get from the nurses or staff that we talk to is that they simply put, selected the wrong drug, or wrong strength, or something like that, when they were inputting the prescription. So it seems to be a lot of the issues on the input end. And then some of them, I think, may be related to translating their information to our system’s information.”* [54]
			*“Technician: “Typically, they [e-prescription errors] occur where either the instructions were ambiguous, depending on what the doctor put in there when they ordered it. Sometimes they occur just by omission by the technician, just either a misunderstanding or processing faster than one ought to.”* [54]
		Mismatch of e-prescription information between prescriber and pharmacy systems	*“Pharmacist: “If we’re searching and it’s not in our system exactly like it’s in the doctor’s system, even a patient name, for example, it won’t come up with that patient. If it’s, say, Cindy Smith but, at the doctor’s office, but we have her entered as Cynthia Smith, it won’t search for Cynthia because it’s searching for Cindy Smith. So then you have to delete out all that information and search for it. Same thing with the drug. If, say, in the doctor’s system it says magnesium citrate, and in our system it says mag citrate, it won’t find the mag citrate because it’s abbreviated differently than what the doctor’s office says. So you have to kind of do a modified search in order to find the right drug.”* [54]
		Mismatch in textbox size	*“One thing I don’t like about the e-scribe, when you get into the dosage on your sheet, sometimes stuff is cut-off. There is not enough room, so you’ve to go in to check to make sure that everything is in there the way it is written on your hardcopy. I am not sure why. There are only so many characters that can go in there and sometimes not all that comes on.* *“What I don’t like is when the drug names are too long, it tells us to ‘see long drug name.’ So you have to remember what came up on the first screen and I think there’s a lot of opportunity for error if you don’t remember what* *or how it came up.”* [52]
		Mismatch in patient/physician names	*“If for example the patient’s name is [Jonathan Doe], and the doctor puts the e-scribe in for Jon. It may say you don’t have that person in the system because they are writing his short name versus his full name or whatever we have. So then it would tell you we don’t have them in there. Or another thing too that is irritating is the doctor’s name that e-scribes is just written different than what’s in our McKesson system,* *it will create them again. So when we look into our list of doctors we have one Dr [Jeff] but there might be 7 in there. Each one might have a different fax, might not have a fax number or has a different fax number.* *That’s one down thing about it is if the doctor that it’s faxed from adds like even a middle initial or something like that, it would create them in there again.”* [52]
		Mismatch related to drug names	*“It doesn’t match the drug. It doesn’t give me choices like this right away. You have to re-enter the drug.* *The problem there is that there could be an error. Because I could choose the wrong drug. You know there’s no link between the drug that comes in over the e-scribe with the drug that we have in our stock. So I have to choose the drug every time. There’s potential for error.”* [52]
		Mismatch with drug quantities	*“Doctors will send over Proair® which is an albuterol inhaler. And in their system it may say that it is an 8 gm canister. When in fact it’s an 8.5 gm canister. So we kind of have to know the sizes of these odd ball things. Because if he sends over a quantity authorized 8 and I put 8 in here. Then it’s billed improperly. I haven’t billed for that 0.5 gm. You know it’s not right so we have to watch that.’* *‘Lantis® injection #2 in this instance is a little deceiving. Our computer has this drug in as 10 because it is a 10 ml per vial. So the doctor is trying to tell us 2 vials. In order to make that quantity correct, I have to make it 20. Because our computer calculates it by ml and not bottles.”* [52]
		Not reported by original authors	*“Prescription received […] 25 mg tablet. Quantity prescribed #90. Sig: “Take 1 tablet (25 mg total) by mouth 2 times a day.” Patient misunderstood this to mean he was to take ½ tablet twice a day to give him a total daily dose of 25mg per day. Dosage should have been 1 whole tablet (25 mg) twice a day for a total daily dose of 50 mg per day. Health system recently changed their programming to include the notation of total dose. I think this will be confusing for the patients - and this is just the first example of a medication error caused by this programming change.”* *“Doctor sent an e-Rx and put one set of directions in the sig and another set of directions in prescriber notes.”* [50]
		Not reported by original authors	*“E-prescription came, but it did not match directly with pharmacy system drug file and pharmacist had to manually choose the strength. The error was not discovered by the pharmacist who entered the drug, nor by the pharmacist who checked the prescription. The patient had a proxy pick up the prescription and the patient discovered the error when it came home. If the pharmacy system matched better to the e-Prescribe Rx product and if the system made it more difficult to choose a non-equivalent product, then this error would not occur so easily.”* [50]
	Software limitations, including e-prescription control and management of outdated notes and prescriptions	Auto-population of e-prescription information	*Technician: “We hear a lot when I call, it defaulted into that, or I don’t know how to get that out of there. So I’m thinking they don’t have a lot of control sometimes.”* *Technician: “They tend not to take their notes off new ones. If they fax over something, they’* *ll say, patient needs to be seen before more refills, but they’* *ll give them a year’* *s worth of refills. And then we find out that that note is old, and they just didn’* *t take it off the e-script.”* [54]
			*Pharmacist: “You’ll see something like that, where you have totally conflicting instructions and I’ve had doctors say, that’s something that’s automatically put in there. I’m just assuming that they have a switch that they can turn on if it says normal sig, or they have to put their sig in. Like if you do doxycycline, it automatically comes up, take one twice a day, and then they put in the day supply or something.”* [54]
			*“Processing refill for patient’s […] ER 10 mEq capsules. Physician e-prescribed […] CR 10 mEq tablets, with the electronic sig of: Take two capsules twice daily. We have seen this many times - with orders getting mixed up on […] capsules vs. tablets. […]- these always come up capsules since this is easier to find in their ordering systems!”* [50]
		Inability of the technology to discontinue old prescriptions	*“One thing with e-scribe, if this person is on this medication that you’ve got to e-scribe and you input it in* *like we just did, it does not DC [discontinue] the old prescription. So you’ve to go back and look at her profile and see if she’s on it. You have to go in and manually do that to keep the profile updated.The profiles I think are a lot more messy when you solely rely on your e-scribe. But we do try to go in and just check.”* *“Another thing that I don’t like and I know reasons people or other pharmacists don’t use it (e-prescribing) is because people won’t go in and discontinue the previous prescription. So a lot of times the profiles just get messy because you are not taking the time to go in and make sure that they get discontinued.”* [52]
		Not updating new prescription after renewing old	*“Copied forward old –redacted- without changing to new –redacted- on new rx”* [58]
		Function-related issues	*“I also acknowledge that the dispensing software used by Mr B does not have a built-in function that identifies retrospective amendments.” (HDC14-HDC)* [58]
		Historical records-related issues	*“His computer history would certainly have shown that Humulin N was what he had had in the past, and that may have been a contributing factor in the error.” (HDC01-Advisor/Pharmacist)* [57]
		QS/1 pharmacy system	*“It’s kind of awkward because we have to flip through these tabs to see the whole thing.’* *‘So now on this screen I don’t have the doctor’s name. So if I am possibly hurrying I have already typed in the doctor’s name but what if I may have forgotten it because I typed it so fast. So now I am wondering who is the doctor who prescribed this.”* *“One of the screens that you fill is the doctor information and then you flip to another screen where you’re filling the actual prescription, and by the time you get to the prescription screen, you have forgotten who the doctor is, and you just typed it in two seconds ago.”* [52]
		Not reported by original authors	*“Pharmacy received 2 E-Rx prescriptions for the same patient on same day by 2 different providers. […] was time stamped at 12:37 by provider A. […] was time stamped at 12:38 by provider B. Provider B was not onsite at this ambulatory outpatient clinic working on that day. Due to high risk medication, different providers, and timing the pharmacist looked in the EMR ([...]) for more information. There was NO record of […] prescribed that day in current or historical records. The apparent computer glitch was reported to IT.”* [50]
		Engagement with technologies	*“The system has been down for about 15 minutes and so a customer’s prescription was dispensed without assistance from the computer. The pharmacist has been on the phone twice to complain and it is currently being seen to.”* [32]
	Absence of automation and technology in community pharmacies and limited integration with other healthcare institutions	Lack of patient database in community pharmacies	*“He dispenses medication based on what information you provide him; nothing [is] documented” (Male pharmacy user group −7)* [33]
			*“There should be a special file for each patient in each pharmacy, not only in the hospital.” (Community pharmacy group-1)* [33]
			*“when Professional group-6 mentioned about the filing lets go even to institutions in the government you find some patients going to different hospitals with no common filing this is a problem starting from the beginning not from the community which is in the end of the road this is one of the problems I know some people going to different hospitals to get the same medication this is I think a problem. However, I am just wondering about it. This the time I think the MOH to upgrade the behaviour [to implement a filling system].” (Professional group-2)* [33]
		Implementation of technology in community pharmacy	*“In America, there is a program that contains the name of the medication to be dispensed drug-drug interaction. This system is good and increases the safety of medications and as to the problem of expiration” Professional group-4)* [33]
		The fragmented healthcare system	*“…And I remember one time one patient like he has two different insurance he went to two different doctors and get the same medication from the different insurance … so we also we need to connect all three [pharmacy, patient, health insurance system] together so we have a system for the insurance for this patient if he has two insurance so he will not abuse this insurance by getting the same kind of medication from different pharmacy or different hospitals.” (Professional group-5)* [33]
			*“For example, the patient went to a physician who prescribed him Amlor [Amlopidine] and then went to another physician who prescribed him Amlopine [Amlopidine]. He imagined that they are different medications and took both.” (Community pharmacy group-4)* [33]
		Lack of electronic/digital prescribing systems	*“At the time of these events, electronic prescribing was available at the DHB but not in the outpatient clinic setting. I consider that, if electronic prescribing had been available to Dr C when she prescribed the medication to Miss A in the Day Stay Unit, it could have minimized the risk of this error occurring.”(HDC19-HDC)* [57]
		Automation	*“If you start looking at the dispensing process as two stages, there’s the mechanical process and there’s the clinical process. [...] [If] you divest the two and use [automation] to take away some of the human error [...] while still allowing the clinical input [...] you stop worrying so much about “Am I gonna make errors that are gonna go out to the patient?” [...] That becomes less of an issue and you start thinking more about “Hang on a minute, is what’s being dispensed in the best interest of the patient anyway?” [Pharmacy owner, Group 10]* [31]

### Theme 1: Pharmacist-related factors

This theme emphasised the perceived lack of pharmacist knowledge, skills, and experience and attributed this as a factor contributing to medication errors in community pharmacies. In two studies, participants described community pharmacists as being inexperienced and lacking competence [33,57], with one participant sharing beliefs that standard operating procedures (SOPs) could or should be followed to mitigate potential errors [57].

*“Unfortunately [the pharmacy] was unaware of [Mr B’s] relative lack of experience, which, if known, could have prompted the need for [Mr B] to read [the pharmacy’s] SOPs. The employment agencies concerned have been spoken with regarding this lack of information, as it is they who are advertising these pharmacists as being fit for purpose, and the agencies have a professional responsibility to their clients to supply competent locum pharmacists.”(HDC13- Advisor/Pharmacist)* [57]

Another participant described an example where a pharmacist was unaware of the availability or existence of specific medications or their strengths, [57] which could have contributed to toxicity or under-dosed treatment as a result of the error.

*“…a major reason for both dispensing errors was that pharmacy staff were unaware at the time of dispensing that both Recormon and tacrolimus came in different strengths. (HDC09-Provider/ Pharmacist)”* [57]

Another participant described a scenario in which a pharmacist, who had been employed at the pharmacy for only 10 days, was required to dispense a prescription. Due to the lack of familiarity with the established patient population in the pharmacy and prescribing patterns, the pharmacist had to rely primarily on the patient’s prior prescription records for uncommon medications [57]. This reliance on historical medication records increases the risk of dispensing errors, particularly when the records are incomplete or contain inaccuracies that have not been rectified.

“[the pharmacist] *had been working at the pharmacy for only 10 days when Ms A’s script was dispensed, which would have made her more reliant than usual on previous script history for unusual scripts.” (HDC26-Advisor/Pharmacist)”* [57]

### Theme 2: The environment within the pharmacy

This theme considers how the following subthemes could contribute to the risk of medication errors: i) limitations of pharmacy space and medication storage, (ii) challenges with medication packaging, and iii) managing typical interruptions within the pharmacy.

#### Subtheme: Limitations of pharmacy space and medication storage.

Participants described ways in which the layout of the pharmacy, specifically the design and configuration of the dispensary area, could predispose to risk of medication errors [31,57]. For example, participants described how organisation and tidiness of shelving systems is important to avoid incorrect ‘picking’ errors [31,57].

*“You’ve got to have a tidy dispensary or things do get muddled up and then when you’re working fast you’re just leaping around, grabbing things off the shelf, and unless things have been put in the correct place it’s so easy [to make a mistake] [Locum pharmacist, Group 9]”* [31]

In two studies, inappropriate medication storage conditions were identified as a causative factor for medication errors [33,57]. Participants raised concerns about disconnecting electricity at night and reconnecting it in the morning, noting that such temperature variations could compromise the quality of medications and potentially lead to their degradation [33]. They also highlighted the issue of storing medications with similar names in close proximity on shelves increasing the risk of errors [57].

*“It is a matter of saving electricity just like groceries. At night, they disconnect the refrigerator containing milk to save electricity, and when they come back in the morning, they turn on the electricity. (Male pharmacy user group-4)”* [33]

#### Subtheme: Challenges with medication packaging.

Participants also expressed concerns about the lack of standardised packaging and labelling in addition to frequent changes of subsidised medications by funded agencies [31,57]. These changes result in variations in appearance and nature of medications increasing the risk for dispensing errors [57].

*“However, it should be pointed out that PHARMAC, the medicines funding authority, does make regular changes to the schedule of funded medicines. This often leads unexpected changes in the appearance and nature of various medicines as different manufacturers are subsidized for the same drug formulation. This situation adds an extra element of risk in today’s dispensing environment, and is an area where pharmacists must be eternally vigilant.”*(HDC10- Advisor/Pharmacist) [57]

#### Subtheme: Managing typical interruptions within the pharmacy.

Across several studies, interruptions in the pharmacy were identified as critical contributors to medication errors in community pharmacy settings [31,32,51,53,56,57,59]. Participants frequently reported experiencing interruptions in workflow due to simultaneous demands such as patient consultations, pharmacy staff communications, ringing telephones, as well as work meetings and conference calls [31,51,53,56,57,59].

“*They use being “salaried exempt” as a way to get free work done from the pharmacists. We are expected to go to work meetings and be on conference calls on our days off unpaid. We are made to rush and do the job of a pharmacist and tech to keep the pharmacy afloat from understaffing. The open environment, being constantly distracted from patients interrupting us (open floor plan), and the phones ringing off the hook with not enough bodies to answer the phone leads to a truly stressful environment that makes it difficult to safely multi-task. More mistakes are being made.”* [51]

One study reported that watching television in the pharmacy during medication dispensing is a source of distraction for pharmacy staff [32].

“*Viewing television seems to be part of work culture. From about 9.15 to noon, the staff was viewing television whilst dispensing medicines. During the television viewing period, attention of the staff was very distracted. JH recorded: 9.18. There is a television playing on the dispensing counter... There is constant attention on the TV. At one point a customer was left waiting as Counter Assistant (CA) was occupied watching TV. She (CA) has been reminded that a customer was waiting. The television was firmly stationed on the front counter and appeared to be part of the pharmacy’s hardware. To confirm this, a researcher collecting data for a different work stream of this project reported watching television (during his lunch break) at this pharmacy*” [32]

### Theme 3: Management and financial related factors

The following subthemes explored the pressures discussed by participants relating to staffing, work-loading and management of the community pharmacy, in addition to financial pressures. This theme included: i) workforce and resource pressures ii) management pressure on pharmacists, and iii) impact of commercial, insurance and financial pressures on pharmacist and physician practices.

#### Subtheme: Workforce and resource pressures.

Staff shortages, as well as difficulties in recruiting and retaining adequately trained and qualified pharmacy personnel, were recognised as significant contributing factors to medication errors in three studies [51,57,59].

*“[Mr B] said the pharmacy was ‘extremely busy’ and he lamented the difficulties in attracting pharmacists to our rural pharmacy’. This is by no means an excuse, but is a very important context for the error.” (HDC09-Advisor/ Pharmacist)* [57]

*“Not enough help! Quality help! Takes a long time to train someone and then they leave for better pay and less stress”* [51]

An additional strain that was discussed by several participants related to workload pressures, including volume of work and long working hours, as being contributors for medication errors [32,51,56,57,59]. Pharmacists often reported managing multiple responsibilities, such as dispensing prescriptions and consulting with multiple patients concurrently. Participants described how this high workload frequently led to rushing prescription processing without a proper clinical check, increasing pharmacist stress and potentially increasing the risk of medication errors [32,33,51,56,57,59].

One participant expressed concerns of making medication errors due to overworking and not taking breaks. The participant also offered recommendations on the enhancement of workplace conditions especially for pharmacists who are pregnant and/or post-partum. Suggestions related to allocation of time and space for resting and breastfeeding to potentially reduce medication errors as a result of pressure and over-working [51].

*“Giving pharmacists a 30 minutes lunch break or 1 hour for pregnant moms to pump and eat can make a huge impact positively in work conditions. Pregnant moms not able to sit or drink water working for more than 12 hours can cause a serious negatively health impact to which I am concerned. When I was pregnant with my first child. I wasn’t able to eat or use the bathroom because I am constantly interrupted with immunizations. i.e., influenza shots, doctor’s calls, patient questions, telephones, technicians needing help, clerk not knowing what to do. The list goes on. Having a chair in every pharmacy for pregnant moms will surely help the mom with her health and making sure her health is taken care of. My biggest concern is making medications error from over working and not taking breaks due to fear of falling behind. Even worse, losing a baby from constantly standing and not able to use the bathroom which lead to UTI which in return does harm to the baby and the mom. The work conditions in community pharmacy is very harsh and dangerous to work in. We cannot afford to make medications error and pharmacists need to eat. We are not robots and I need to eat for my growing baby.”* [51]

Moreover, pharmacists also articulated it was challenging to find time for professional development due to workload pressures [33].

*“He has long working hours and that leads many pharmacists to not refresh their information.[by attending workshops for continuing their education], …there is no role for the Ministry to update your information. For example, the pharmacist (Community pharmacy group-4) graduated in 1986 and necessarily many improvements[updates] have taken place since that time.” (Community pharmacy group-2)* [33]

In several studies, pharmacists indicated the financial difficulties faced [31–33,51], with funding [33] and wage and staff cuts [31] being examples that then impacted on the risk of errors occurring. One study, in particular, reflected specifically on balancing the profitability of a pharmacy as a business alongside needing to ensure patient safety [33]. In this study, participants described the pharmacists’ frustration with having to work on a Friday, which is typically considered a weekend in Saudi Arabia [33].

*“I don’t expect anything from the pharmacist, because all pharmacists are frustrated and this is due to their low salaries and the nature of their work. In a pharmacy, I noticed a pharmacist working in the middle of a hot day on Friday and he used to walk three or four kilometres.” (Male pharmacy user group −5)* [33]

In one study, there was description of how decreased investment in automation and safety-enhancing pharmacy operations may also contribute to medication errors [51].

*“Pharmacies used to be able to afford safety features and automation but even that cannot longer be afforded”* [51]

#### Subtheme: Management pressure on pharmacists.

Pharmacists underscored the tension between management-directed performance metrics and patient-centred care in pharmacies [32,33,51]. Pharmacists reported feeling compelled to meet prescription targets, rather than focusing on patient outcomes; many described how they may face disciplinary actions if they were non-compliant with reaching targets [33,51].

*“Problems between you and the owner arise; he [the owner] asks what happened … [you] pay a penalty, close the pharmacy and your license is suspended. These problems face us and affect our work.” (Community pharmacy group-4)* [33]

In two studies, participants reported it was a struggle to provide comprehensive consultations, such as systematic ‘Medication Use Review (MUR)’, due to time limitations and management surveillance [32,51].

*“It is conflicting, because you care about your patients and you think it’s not about targets. Like the old lady that came in and she was talking about her painkillers when she has a headache. I noted that down, I could use that as an MUR in the sense that I did sort of tell her that she’s not supposed to be taking them all the time. It’s good that you can use it, because it’s exactly what it is, you are telling the patient how to use them and advising them about it. Those are the little things that I have to concentrate on and pick up on to meet my targets.”* [32]

In these studies, pharmacists also indicated that their clinical expertise was undermined when their professional judgment was being questioned for the purpose of enhanced patient satisfaction [32,51]. They also expressed frustration over the management holding them responsible for patient complaints, even if they had valid reasons for refusing to fill prescriptions or dispense medications [32,51].

*“If I get a complaint I could be written up even if I had a valid reason that they had to wait I am always in the wrong and must call to apologize even if they were wrong the patient is never wrong and my professional judgment on not filling or refusing a patient is constantly being questioned and must be defended against a patient who doesn’t know anything about my job or the logistics of pharmacy billing dispensing and law.”* [32]

#### Subtheme: Impact of commercial, insurance and financial pressures on pharmacist and physician practices.

Participants conveyed that some pharmacists and physicians were affected by financial incentives from pharmaceutical companies or driven by their profit motives leading to prescribing and dispensing of specific medications rather than addressing patient needs [31,33]. If medications dispensed are inappropriate for the patient, they may place the patient at risk of harm.

*“In our country, the pharmacist gives you the medication that is suitable to him or the medication for which he receives a commission, you go to the pharmacist you say you have a headache he gives you Fevadol instead of Panadol [generic substitute] for example he gives you the medication that suits you, who he is an agent for it or gives him a commission for. Even in clinics, the representative of the company comes to the physician and gives him the new medications with tickets and gifts and the physician prescribes the medications.” (Male pharmacy user group-5)* [33]

Additionally, participants discussed the impact of insurance companies on physicians’ prescribing behaviours indicating that physicians overprescribed medications to increase the insurance claims [33].

Moreover, they shared the role of Pharmacy Benefit Managers (PBMs) in restricting patients’ choices forcing them to use certain chain pharmacies, that are understaffed, therefore potentially increasing the risk for errors [51].

### Theme 4: Organisational and social environment within the pharmacy

This theme includes the following subthemes i) error mitigating policies and processes ii) human errors in pharmacy practice iii) communication challenges and iv) unrealistic expectations about pharmacist practice

#### Subtheme: Error mitigating policies and processes.

In two studies, participants reported instances of inadequate and outdated SOPs and policies; in the examples they shared, these related to high-risk scenarios such as methadone dispensing, controlled drug storage, and incident management [57,59].

*“However, I note that the pharmacy had preferred practices that it encouraged pharmacists to employ relating to its checking procedures that were not included in the SOPs at the time of events. I am critical of the pharmacy that its SOPs were not up to date to reflect its current practices.” (HDC31-HDC)* [57]

In five studies, participants identified that medication errors were concealed and underreported, rather than being recognised and addressed, suggesting failure in good clinical governance [31,32,51,57,59].

*“I know other pharmacists who [...] definitely stick to the rules, no matter what, and are not gonna bend ‘em. Then some people who kind of just squeeze past them. So it does depend on the person [Pharmacist, Group 2]”* [31]

*“There are colour baskets red, white and blue, which are supposed to be used to organize dispensing, but I have just been informed that this procedure is not usually followed.”* [32]

*“In my view, the number of errors at the pharmacy, and the fact that these have been made by more than one staff member, indicate a systemic problem with regard to staff failing to follow the pharmacy’s SOPs.” (HDC14-HDC)* [57].

Differences in policy adherence were also noted between chain and independently-ran pharmacies. In two studies, participants shared their perspectives of chain pharmacies maintaining a firmer compliance to guidelines to ensure standardisation across all branches, compared to those that were independent [31,33]. Moreover, they stated that chain pharmacies provide training for their pharmacy staff to emphasise the implementation of regulations [31,33].

*“The advantage of notifying the Head Office [of a chain] is that they then cascade the information to everybody so that every store can then separate the Xalacom and Xalitan in the fridge so that it doesn’t happen, so you’re actually avoiding the error ever happening [Locum pharmacist, Group 9]”* [31]

Participants also noted across three studies the challenges of balancing adherence to protocols with the need for flexibility in clinical decision making and personalised patient care [31,33,59]. Some pharmacists indicated their readiness to bypass SOPs where compliance might not adequately address the patient’s needs, especially in humanitarian or critical cases or cases involving medication loaning [31,33,59].

*“I sometimes think they’re not very helpful for patients. If you’ve got a protocol and you’ve got to do it a certain way, but then you can’t, say a checking one [...] do you break it for the benefit of the patient, even though you know you shouldn’t? [Pharmacist, Group 2]* [31]

*“I dispense everything; I am a pharmacist regardless of the laws, when you have a patient in front of you needing to be treated it would be difficult especially if the patient is poor and needs assistance you do not help him; for humanity” (Community pharmacy group-4)* [33]

Furthermore, in one study pharmacists indicated that despite promoting a learning-centred work environment, a blame culture still persisted with emphasis on who committed the error, which could hinder accountability and transparency in reporting [31].

*“[We] were taught, certainly in my era, at College, you did not make mistakes, you covered them up, that was the history. I had a boss who I could have killed because he did make mistakes but he refused to admit it. [...] [I] went in to the dispenser and said “Look, ignore him, we all make mistakes, we check each other” [Locum pharmacist, Group 9]* [31]

Participants in three studies also reported poor error reporting practices reflecting inconsistent, informal and non-transparent approaches for reporting [31,56,57].

*“Amending records in this way without identifying that the amendment has been made retrospectively is very poor practice. Furthermore, by making amendments to the records in this way, Mr B removed the record of what actually occurred, which is also unacceptable.” (HDC14-HDC)* [57]

#### Subtheme: Human errors in pharmacy practice.

Three studies identified human errors as significant risks to patient safety [31,57,58]. These errors encompassed inattentiveness, forgetfulness, inadequacies in labelling, packaging, and bagging in addition to misidentification of patients and medications and insufficient assessment of patient-specific factors before medication dispensing [31,55,57,58].

*“In this case, it appears that human error or haste on the part of Mr D (or another pharmacy staff member) caused Mrs A’s prescriptions to be bagged incorrectly.” (HDC08-HDC)* [57]

#### Subtheme: Communication challenges.

In two studies, participants expressed a key challenge faced by pharmacists: the lack of communication among pharmacy staff and within and between branches of chain pharmacies [31,56]. Furthermore, pharmacists were often concerned about not being informed of recent errors and incidents [31,56].

*“I [once] had an issue with methadone, [...] I did not agree with what the pharmacists had done the previous week and they’d done nothing about it all week when they had time to sort it out and then they didn’t even tell me in advance of me going, I walk in on the Saturday and get stuck with the real issue, do you give it, don’t you give it. And you’re dealing with something that’s quite, you know, can change that person quite a lot, and you’re thinking, “Well, where do you stand?” [Locum pharmacist, Group 3]* [31]

An additional issue that participants indicated in one study is the breach of patient confidentiality through verbal communication particularly in small community pharmacies [53].

*“There is more potential to break HIPAA [U.S. Health Insurance Portability and Accountability Act, a legislative act that protects patients’ health information]. Because we have a small pharmacy, I’ll be over here [pointing to opposite side of pharmacy] and I’ll say, ‘Hey, can you pull a prescription for [John Doe],’ or whomever the patient may be. I’ll say it across the pharmacy, so the other patients know who the person is. Whereas with the paper prescription, it’s there and you don’t even have to say anything.”* [53]

Moreover, some pharmacists conveyed feeling demoralised as a result of strict company policies that hinder open communication and undervalue the importance of feedback. They also shared feeling nervous when addressing issues that include colleagues about potential negative consequences [31].

*“I think you tend to get more demoralised staff in a company and more negative on feedback and communication to a well-run independent sometimes, because I think they’re sort of all put into a block and they can be boxed if you’re not careful in a company”* [31]

Additionally, across three studies, participants identified communication gaps between pharmacists and patients [33,53,57] with language barriers being one of the contributing factors.

*“I came across someone who didn’t know whether the medication was for constipation or diarrhoea. He said he wanted something for diarrhoea. The matter is that he didn’t want something for diarrhoea; he wanted something to cause diarrhoea. In brief, language has an effect.” (Community pharmacy group-4)* [33]

In one study, some pharmacists highlighted that these communication issues are more common with e-prescriptions compared to paper prescriptions. Patients often perceive that e-prescriptions are instantly processed resulting in their dissatisfaction as a result of the delay in receiving their medications [53].

Participants also reported an argument about pharmacists’ role in patient education. Some believed that they should proactively offer patient education even if the patient does not ask while others argued that education should only be offered upon patient request. This discrepancy in expectations can potentially affect medication safety [33]. Participants also indicated having communication challenges between pharmacists and prescribers, particularly when contacting prescribers to clarify prescriptions [33,53].

*“There is another concern in that it doesn’t always print the information of the doctor where they’re currently at. If you have a doctor working a walk-in or an E.R. [emergency room] and you get a prescription from them, it sometimes lists their office phone number. Then if you have a question on it and you try to call the phone number that’s on the e-prescription sometimes they’re like, ‘Oh, well they’re not here today.’ Then you have to call around or they have to bounce you around from person to person until you can find where they actually are practicing. So it makes it harder to track them down.”* [53]

#### Subtheme: Unrealistic expectations about pharmacist practice.

Pharmacists reported having sometimes tense interactions with patients resulting in pressure affecting their dispensing practices and decisions [33,57]. In one study, participants described a case scenario in which patients pressured pharmacists to dispense antibiotics by the strip, a practice that is considered a violation in Saudi Arabia. [33].

*“Originally, it is prohibited by the Ministry of Health to dispense antibiotic as a strip and if this is done it would be a violation and in case of not dispensing them in this form, the patient will go to a second, third and fourth pharmacy until he finds what he wants” (Community pharmacy group-1)”* [33]

Moreover, in three studies, pharmacists highlighted patients’ lack of understanding of the dispensing process and their expectation that an effective pharmacist is one who fills prescriptions quickly without considering accuracy and quality. This created an extra pressure on pharmacists as they often felt obligated to prioritise patient satisfaction over appropriate medication dispensing and consultation [31,51,59].

*“I think we’ve got to get away from the idea that a good pharmacist in the view of the public is one who gets the medication out quickly. [...] They just assume that it’s going to be correct but they don’t rank the actual quality of the dispensing in any of it, they put speed at the top [Locum pharmacist, Group 9]”* [31]

### Theme 5: Challenges with digital technologies

This theme includes these subthemes i) prescription input error, system translation and incompatibility issues ii) software limitations, including e-prescription control and management of outdated notes and prescriptions and iii) absence of automation and technology in community pharmacies and limited integration with other healthcare institutions.

#### Subtheme: Prescription input error, system translation and incompatibility issues.

Pharmacists listed several challenges associated with e-prescription systems, including input errors by medical staff, discrepancies between the pharmacy and the prescriber systems, and confusion in prescription details [50,52,54]. As a result of these challenges, several medication errors were undetected by pharmacists and reached patients [50].

*“Pharmacist: If we’re searching and it’s not in our system exactly like it’s in the doctor’s system, even a patient name, for example, it won’t come up with that patient. If it’s, say, Cindy Smith but, at the doctor’s office, but we have her entered as Cynthia Smith, it won’t search for Cynthia because it’s searching for Cindy Smith. So then you have to delete out all that information and search for it. Same thing with the drug. If, say, in the doctor’s system it says magnesium citrate, and in our system, it says mag citrate, it won’t find the mag citrate because it’s abbreviated differently than what the doctor’s office says. So you have to kind of do a modified search in order to find the right drug.”* [54]

**Subtheme: Software limitations, including e-prescription control and management of outdated notes and prescriptions**

Five studies identified challenges with pharmacy-based software as potential causes of medication errors [32,50,52,57,58], including lack of change-tracking features, technical malfunctions and system downtimes.

*“The system has been down for about 15 minutes and so a customer’s prescription was dispensed without assistance from the computer. The pharmacist has been on the phone twice to complain and it is currently being seen to.”* [32]

Several issues with e-prescription systems were highlighted, including outdated information and automated default settings which limited user control and created discrepancies with prescribers’ intentions [50,53,54,58].

*“Technician: “We hear a lot when I call, it defaulted into that, or I don’t know how to get that out of there. So I’m thinking they don’t have a lot of control sometimes.”*

*Technician: “They tend not to take their notes off new ones. If they fax over something, they’ll say, patient needs to be seen before more refills, but they’ll give them a year’s worth of refills. And then we find out that that note is old, and they just didn’t take it off the e-script.”* [54]

#### Subtheme: Absence of automation and technology in community pharmacies and limited integration with other healthcare institutions.

Pharmacists noted that the lack of patient records, electronic prescribing, or automation in pharmacies increases the risk of medication errors [31,33,57]. They also highlighted that the absence of an integrated system connecting pharmacies, patients, and insurance providers could result in duplicate prescriptions and potential harm [33].

*“There should be a special file for each patient in each pharmacy, not only in the hospital.” (Community pharmacy group-1)* [33]

##### Study quality appraisal:

All 13 assessed studies satisfied the criteria related to the clarity of the research aims and findings, the appropriateness of the qualitative methodology and the value of the research [31–33,50–59]. Nevertheless, 12 studies lacked sufficient information regarding whether the relationship between the researcher and the participants was considered [31–33,50–59]. Moreover, six studies did not meet the criterion assessing on whether the data was collected in a way that addressed the research issue [31,32,50,51,54,57]. The details of the quality appraisal are outlined in S4 File.

### Confidence in the synthesised findings

The GRADE-CERQual confidence in the synthesised findings are depicted in S5 File.

## Discussion

While previous reviews have investigated the factors or causes of medication errors in community pharmacies [60–62], this study is the first meta-ethnographic systematic review to assess the causes of medication errors in the community pharmacy setting. The overarching themes developed in this work demonstrated the multifaceted causes of such errors, ranging from pharmacist-related factors to organisational- and management- related factors.

Shortage of staff, high workload and interruptions were frequently reported contributors to medication errors in community pharmacies. Pharmacies are often considered as busy and fast-paced environments where interruptions in workflow are common [63–66]; such disruptions have been deemed to impact on pharmacists’ ability to concentrate, especially when engaged in highly cognitive medication safety activities, such as the clinical review of prescriptions [63,65,67]. Well-structured workspaces have been recognised to enhance pharmacists’ focus and to reduce the potential of medication errors [68,69], echoing the findings from this work. Staff shortages and increases in patient numbers have been recognised as attributing to rises in workload [70–72], thereby reducing time allocated for individual patient care and potentially compromising patient safety. To address these challenges, several interventions may be considered. These include redesigning pharmacy facilities to separate order entry, dispensing, and prescription checking activities and introducing non-interruption zones for tasks that require sustained concentration [73]. System level changes may also be effective such as implementing dispensing tracking systems, automated dispensing technologies, and a pharmacy services call centre staffed by pharmacy technicians, alongside protected checking time to minimise interruptions during cognitively demanding tasks [73]. In addition, staff training and role standardisation could support efficient workload management [74]. For instance, assigning staff to specific work areas based on task urgency and ensuring they acquire the competencies needed for their roles [74]. Providing pharmacists with access to prescribers’ electronic health records may further improve workflow efficiency [73]. Moreover, structured task allocation across the pharmacy team and proactive workforce planning to maintain a tolerable workload are also likely to contribute to safer practice. [73–75].

Moreover, this review recognised several organisational-, cultural- and communication- related factors contributing to medication errors in community pharmacies. A culture of error concealment, underreporting, and reliance on informal mechanisms for mitigating incidents echoed previous studies [76,77], specifically fear of professional or disciplinary consequences, a lack of trust in the fairness of regulatory systems and fear of personal blame [78–80]. The presence of a blame culture has been well-documented as a barrier to medication safety across various healthcare settings [79,81,82]. Adding to these challenges is the tendency of pharmacists in this review to prioritise their professional judgement over adherence to policies and standardised protocols [31,33,59].Several published studies depicted circumstances in which pharmacists deviated from protocols, indicating a potential contradiction between pharmacists’ perceptions of the best course of action and regulatory requirements [31,83]. Pharmacists’ clinical decision making and compliance with protocols can be influenced by various factors including pharmacists’ experience, the patient’s clinical context and protocol obsolescence [31,83]. This review also noted protocol obsolescence as a reason for non-adherence. Wider studies have shown that while pharmacists’ actions often indicate their dedication to medication safety, dependence on outdated guidance or divergence from policies has been associated with an increased risk for adverse events [75,84]. Hence, it is essential to regularly review and update pharmacy policies to ensure alignment with the latest advancements in community pharmacy practice. This study also highlighted the centralised role of chain pharmacies in promoting patient safety by implementing standardised policies and procedures and sharing resources across multiple branches. However, similar to other studies [62,68,85], it pointed out that communication gaps between pharmacy branches and among staff contribute to medication errors. Effective team communication is key for establishing a patient safety culture [86,87]. Pharmacy management should assess the impact of communication gaps to identify areas requiring improvement [88]. Addressing deficiencies in team communication, specifically those affecting collaboration, patient information exchange, and responsibility for patient care, is vital. Hence, strategies such as promoting open discussions, implementing dedicated committees, and conducting regular briefings should form integral components of any patient safety intervention within the community pharmacy sector [88]. Collectively, these review findings emphasise the importance of adopting a patient safety culture defined by adherence to SOPs for error reporting and supported by a blame-free, non-punitive and learning environment [75].

The study findings also raised concerns regarding pharmacist–patient communication including the possible association between medication errors and language barriers. Previous evidence has demonstrated inequity around access to medications and healthcare services for people with cultural and linguistic diversity [89–92], exposing certain populations of people to a greater risk of medication errors and adverse effects [93]. Further, inequalities relating to involvement in decision-making processes about medicines have been demonstrated amongst people experiencing minoritisation, which further increases the potential of medication errors [45,94,95]. There is a growing need for pharmacists to embrace communication and involvement strategies aligned with patients’ needs and therapeutic goals [96,97], with the ultimate aim of achieving optimal health outcomes [96,97]. Echoing previous studies [62,98], difficulty in communicating with prescribers was also identified as a factor negatively impacting medication safety and patient outcomes. [99] Several factors may contribute to these challenges including limited physician time, attitudes regarding pharmacists’ inadequate competency and physicians’ self-perception of sole responsibility for medications [100].Facilitating pharmacist access to the patients’ electronic medical records could serve as a potential solution to overcome this barrier [101]. Furthermore, implementing a collaborative care model, starting with training the pharmacists, improving the physicians’ awareness of the pharmacists’ role, followed by cultivating their professional recognition and commitment, may further assist in collaborative care efforts [100–102].

This review identified human factors such as lapses in attention, memory failures, and deficiencies in medication preparation and dispensing processes as contributors to medication errors. These findings are consistent with current literature, which highlights cognitive errors as a prevalent cause of medication errors in community pharmacy settings [62]. Medication management is a cognitively demanding process involving clinical reasoning, reflective thinking, problem-solving, and decision-making [98]. These aspects fall under the domain of cognitive ergonomics, which is a discipline that studies how cognitive processes influence interactions among humans and performance in complex tasks [103]. According to this discipline, when individuals are required to perform multiple high-level cognitive tasks, particularly in high-pressure environments, memory lapses are more likely to occur [104]. Consequently, relying solely on memory and attention increases the risk of medication errors. To mitigate this, SOPs a well-organised distribution of duties among pharmacy staff and integrating technological solutions can decrease individual cognitive burden and minimise the risk of human errors [75]..

Additionally, this review highlighted how both patient- driven and commercial pressure can affect medication safety in community pharmacy practice. Pharmacists often face demands to dispense medication rapidly which may limit the time available for thorough review of prescriptions which is an essential step to prevent errors [105]. Furthermore, financial and commercial pressure, especially from the pharmaceutical industry, can significantly influence prescribing behaviours and clinical decision making potentially misaligning with patients’ best interests, compromising their safety and increasing healthcare costs [106,107]. Tackling these challenges necessitates multi-dimensional interventions including public awareness campaigns to support the role of pharmacists in prescriptions’ review, pharmacy workflow improvement, designated clinical review spaces, and pharmacists’ training on effective management of patient pressure [88]. Regulatory strategies are also needed to reduce undue influence of pharmaceutical industry [108,109] and to ensure that treatment decisions are guided by clinical evidence and cost-effectiveness studies [110].

Absence of pharmacy automation and the limited integration of community pharmacy systems with other healthcare institutions were also found to contributing factors to medication errors. In fragmented care settings, pharmacists often lack access to electronic health records increasing the risk for drug duplication and patient harm [111,112] Implementing technological solutions in community pharmacies is crucial and automation offers several advantages, including reduced prescription filling time [113,114], enhanced productivity and dispensing efficiency, and overall healthcare cost savings [115,116]. However, technology is not without risks [116,117].E-prescribing systems may compromise medication safety, particularly when outdated prescription information is auto-populated and is electronically transmitted to pharmacies as identified in this review and in prior studies [62]. Design and functionality limitations in e-prescribing and dispensing technologies were noted to disrupt workflow and impair community pharmacists’ ability to deliver safe and effective care. Overcoming these problems requires changes to existing technological systems in community pharmacies including the establishment of effective communication channels between prescribers and pharmacists [50]. Adherence to evidence-based best practices and guidelines for safe electronic communication of medication information such as those issued by the Institute for Safe Medication Practices (ISMP) is also essential [50,118]. Providing training for both prescribers and community pharmacists on the optimal use of electronic prescribing and dispensing systems may also reduce misunderstanding and contribute to the prevention of medication errors [119].

This review presents several findings with notable implications for community pharmacy practice and future research. The overarching third-order constructs developed from the synthesis indicate that an intricate interplay of factors contributes to medication errors in community pharmacy settings. Further research is warranted to explore the interrelationships among these factors given the inherent complexity of community pharmacy environments. Mapping these connections is a critical step in designing effective interventions. Future studies should similarly focus on identifying the best frameworks for implementing quality and safety strategies and evaluating their impact in real-life settings.

### Strengths and limitations

This review has numerous strengths. Firstly, it represents the first meta-ethnography of the causes of medication errors within community pharmacies. Secondly, the methodological rigor of the review process, as the findings were reported in accordance with the PRISMA guidelines [34] and the eMERGe framework [35]. Moreover, the review used an extensive search strategy that was not restricted by language or publication date and included supplementary searches thereby enhancing the robustness of the search. However, only a limited number of studies reporting qualitative data were identified within the wider literature, emphasising the need for additional qualitative research in this area. Furthermore, the majority of studies were conducted in the United Kingdom and the United States which may limit the generalisability of the results to other areas.

## Conclusions

Recognising opportunities to enhance medication safety in community pharmacies requires a thorough examination of the factors contributing to medication errors in this setting. This meta-ethnographic systematic review synthesised qualitative findings and developed five third-order constructs that capture the underlying causes of such errors. These constructs include pharmacist-related factors, the environment within the pharmacy, management and financial related factors, organisational and social environment within the pharmacy and challenges with digital technologies The findings have important implications for policymakers, healthcare leaders, pharmacy managers, and community pharmacists as they design and implement quality and safety interventions tailored to these domains. Further research is necessary to deepen our understanding of the interrelationships among these factors which is essential for the creation of effective context-sensitive interventions and for evaluating their impact on medication safety in real-world community pharmacy practice.

## Supporting information

S1 File PRISMA 2020 Checklist. (DOC)

S2 File The eMERGe checklist for meta-ethnography. (DOC)

S3 File Data Sources and Search Strategies. (DOCX)

S4 File Results of the risk of bias assessment of the included studies. (DOC)

S5 File CERQual assessments. (DOC)
