# Supplementary material for: Causes of medication errors in community pharmacies: A meta-ethnography and systematic review
Source: PLoS One. 2026 Jun 10;21(6):e0349120. doi: 10.1371/journal.pone.0349120 (PMC13252845; doi:10.1371/journal.pone.0349120)
Supplement: S3 File — (DOCX) [file pone.0349120.s003.docx]

**Supplementary File 3. Data Sources and Search Strategies**

Medline (Ovid)

1. community pharmacy.mp. or exp Pharmacies/

2. community pharmacist.mp. or exp Pharmacists/

3. exp Pharmacies/ or private pharmacy.mp.

4. exp Pharmacists/ or exp Community Pharmacy Services/ or private pharmacist.mp. or exp Pharmacies/

5. exp Ambulatory Care/ or outpatient pharmacy.mp.

6. outpatient pharmacist.mp.

7. ambulatory pharmacy.mp.

8. ambulatory pharmacist.mp.

9. retail pharmacy.mp.

10. retail pharmacist.mp. or exp Pharmacists/

11. pharmacy store.mp.

12. pharmacy shop.mp.

13. exp Medication Errors/ or drug error.mp.

14. drug safe*.mp.

15. exp Harm Reduction/ or drug harm.mp.

16. drug risk.mp.

17. drug event.mp.

18. drug problem.mp.

19. drug incident.mp.

20. drug concern.mp.

21. drug quality.mp.

22. drug interaction.mp. or exp Drug Interactions/

23. drug discrepancy.mp.

24. exp Inappropriate Prescribing/ or inappropriate drug.mp.

25. drug intervention.mp.

26. drug manag*.mp.

27. drug mistake.mp.

28. drug violation.mp.

29. drug omission.mp.

30. drug omit.mp.

31. drug comission.mp.

32. drug commit.mp.

33. drug mishap.mp.

34. improper drug.mp.

35. wrong drug.mp.

36. incorrect drug.mp.

37. nearmiss.mp.

38. drug misadventure.mp.

39. drug oversight.mp.

40. medication safe*.mp.

41. medication safety.mp.

42. medication harm.mp.

43. medication risk.mp.

44. medication event.mp.

45. medication problem.mp.

46. medication incident.mp.

47. medication concern.mp.

48. medication quality.mp.

49. medication interaction.mp.

50. medication discrepancy.mp.

51. inappropriate medication.mp.

52. medication intervention.mp.

53. medication management.mp.

54. medication mistake.mp.

55. medication violation.mp.

56. medication omission.mp.

57. medication commission.mp.

58. community pharmacy.mp. or exp Pharmacies/

59. community pharmacist.mp. or exp Pharmacists/

60. exp Pharmacies/ or private pharmacy.mp.

61. exp Pharmacists/ or exp Community Pharmacy Services/ or private pharmacist.mp. or exp Pharmacies/

62. exp Ambulatory Care/ or outpatient pharmacy.mp.

63. outpatient pharmacist.mp.

64. ambulatory pharmacy.mp.

65. ambulatory pharmacist.mp.

66. retail pharmacy.mp.

67. retail pharmacist.mp. or exp Pharmacists/

68. pharmacy store.mp.

69. pharmacy shop.mp.

70. exp Medication Errors/ or drug error.mp.

71. drug safe*.mp.

72. exp Harm Reduction/ or drug harm.mp.

73. drug risk.mp.

74. drug event.mp.

75. drug problem.mp.

76. drug incident.mp.

77. drug concern.mp.

78. drug quality.mp.

79. drug interaction.mp. or exp Drug Interactions/

80. drug discrepancy.mp.

81. exp Inappropriate Prescribing/ or inappropriate drug.mp.

82. drug intervention.mp.

83. drug manag*.mp.

84. drug mistake.mp.

85. drug violation.mp.

86. drug omission.mp.

87. drug omit.mp.

88. drug comission.mp.

89. drug commit.mp.

90. drug mishap.mp.

91. improper drug.mp.

92. wrong drug.mp.

93. incorrect drug.mp.

94. nearmiss.mp.

95. drug misadventure.mp.

96. drug oversight.mp.

97. medication safe*.mp.

98. medication safety.mp.

99. medication harm.mp.

100. medication risk.mp.

101. medication event.mp.

102. medication problem.mp.

103. medication incident.mp.

104. medication concern.mp.

105. medication quality.mp.

106. medication interaction.mp.

107. medication discrepancy.mp.

108. inappropriate medication.mp.

109. medication intervention.mp.

110. medication management.mp.

111. medication mistake.mp.

112. medication violation.mp.

113. medication omission.mp.

114. medication commission.mp.

115. medication mishap.mp.

116. improper medication.mp.

117. wrong medication.mp.

118. incorrect medication.mp.

119. nearmiss.mp.

120. medication misadventure.mp.

121. medication oversight.mp.

122. safe therapy.mp.

123. therapy error.mp.

124. therapy risk.mp.

125. therapy event.mp.

126. therapy harm.mp.

127. therapy problem.mp.

128. therapy incident.mp.

129. therapy concern.mp.

130. therapy quality.mp.

131. therapy interaction.mp.

132. therapy discrepancy.mp.

133. Inappropriate therapy.mp.

134. therapy management.mp.

135. therapy mistake.mp.

136. therapy violation.mp.

137. therapy omission.mp.

138. therapy omit.mp.

139. therapy commission.mp.

140. therapy commit.mp.

141. therapy mishap.mp.

142. medication duplication.mp.

143. therapy duplication.mp.

144. drug duplication.mp.

145. therapy mishap.mp.

146. improper therapy.mp.

147. wrong therapy.mp.

148. incorrect therapy.mp.

149. therapy misadventure.mp.

150. therapy oversight.mp.

151. prescribing error.mp.

152. prescription error.mp.

153. prescribing harm.mp.

154. prescription harm.mp.

155. prescription risk.mp.

156. prescribing risk.mp.

157. prescribing problem.mp.

158. prescription problem.mp.

159. prescribing incident.mp.

160. prescription incident.mp.

161. prescription concern.mp.

162. prescribing concern.mp.

163. prescription quality.mp.

164. prescribing quality.mp.

165. prescription discrepancy.mp.

166. prescribing discrepancy.mp.

167. inappropriate prescription.mp. or exp Inappropriate Prescribing/

168. improper prescribing.mp.

169. improper prescription.mp.

170. prescribing intervention.mp.

171. prescription intervention.mp.

172. prescribing mistake.mp.

173. prescription mistake.mp.

174. prescription violation.mp.

175. prescribing violation.mp.

176. prescription mishap.mp.

177. prescribing mishap.mp.

178. wrong prescription.mp.

179. wrong prescribing.mp.

180. incorrect prescription.mp.

181. incorrect prescribing.mp.

182. prescription misadventure.mp.

183. prescribing misadventure.mp.

184. dispensing error.mp.

185. dispensing harm.mp.

186. dispensing risk.mp.

187. dispensing event.mp.

188. dispensing problem.mp.

189. dispensing incident.mp.

190. dispensing concern.mp.

191. dispensing quality.mp.

192. dispensing discrepancy.mp.

193. inappropriate dispensing.mp.

194. dispensing mistake.mp.

195. dispensing violation.mp.

196. dispensing omission.mp.

197. dispensing commission.mp.

198. dispensing duplication.mp.

199. dispensing mishap.mp.

200. improper dispensing.mp.

201. wrong dispensing.mp.

202. incorrect dispensing.mp.

203. dispensing misadventure.mp.

204. dispensing oversight.mp.

205. transcribing error.mp.

206. transcribing harm.mp.

207. transcribing risk.mp.

208. transcribing event.mp.

209. transcribing problem.mp.

210. transcribing incident.mp.

211. transcribing concern.mp.

212. transcribing quality.mp.

213. transcribing discrepancy.mp.

214. inappropriate transcribing.mp.

215. improper transcribing.mp.

216. transcribing mistake.mp.

217. transcribing duplication.mp.

218. wrong transcribing.mp.

219. incorrect transcribing.mp.

220. transcribing misadventure.mp.

221. administration error.mp.

222. administration harm.mp.

223. administration event.mp.

224. administration risk.mp.

225. administration problem.mp.

226. administration incident.mp.

227. administration concern.mp.

228. administration discrepancy.mp.

229. inappropriate administration.mp.

230. administration mistake.mp.

231. administration violation.mp.

232. administration omission.mp.

233. administration commission.mp.

234. administration duplication.mp.

235. administration mishap.mp.

236. improper administration.mp.

237. wrong administration.mp.

238. incorrect administration.mp.

239. administration misadventure.mp.

240. administration oversight.mp.

241. inappropriate route of administration.mp.

242. wrong route of administration.mp.

243. incorrect route of administration.mp.

244. improper route of administration.mp.

245. wrong dose.mp.

246. incorrect dose.mp.

247. inappropriate dose.mp.

248. improper dose.mp.

249. dose duplication.mp.

250. dose commission.mp.

251. dose omission.mp.

252. overprescribing.mp.

253. error in calculation.mp.

254. inappropriate calculation.mp.

255. improper calculation.mp.

256. wrong calculation.mp.

257. calculation problem.mp.

258. exp "Root Cause Analysis"/ or Cause.mp.

259. factor.mp. or exp Time Factors/

260. human.mp. or exp Humans/

261. work/ or "personnel staffing and scheduling"/

262. work.mp.

263. exp Financial Stress/

264. stress.mp. or exp Occupational Stress/ or exp Stress, Psychological/

265. exp Burnout, Psychological/ or exp Burnout, Professional/ or burnout.mp.

266. system factor.mp.

267. system.mp.

268. element.mp. or exp Elements/

269. organization*.mp.

270. origin.mp.

271. root.mp.

272. provocation.mp.

273. source.mp.

274. incentive.mp. or exp Motivation/

275. occasion.mp.

276. explanation.mp.

277. catalyst.mp.

278. stimulus.mp. or exp Generalization, Stimulus/

279. trigger.mp.

280. risk factor.mp. or exp Risk Factors/

281. origin.mp.

282. Ergonomics.mp. or exp Ergonomics/

283. systems analysis/ or systems integration/ or workflow/

284. motive.mp.

285. goal.mp. or exp Goals/

286. impetus.mp.

287. rationale.mp.

288. basis.mp.

289. ground.mp.

290. etiology.mp.

291. reason.mp.

292. engineer*.mp.

293. interface.mp.

294. component.mp.

295. aspect.mp.

296. variable.mp.

297. influence.mp.

298. determinant.mp.

299. contributor.mp.

300. parameter.mp.

301. feature.mp.

302. challenge.mp.

303. barrier.mp.

304. obstacle.mp.

305. facilitator.mp.

306. qualitative design.mp. or exp Interviews as Topic/ or Qualitative Research/

307. mixed methods.mp.

308. focus group.mp. or exp Focus Groups/

309. ethnograph*.mp.

310. exp Anthropology, Cultural/ or exp Anthropology/ or anthropology*.mp.

311. exp Interview, Psychological/ or exp Interview/ or interview.mp.

312. theme.mp.

313. thematic.mp.

314. code.mp.

315. quote.mp.

316. field note.mp.

317. observation.mp. or exp Observation/

318. 1 or 2 or 3 or 4 or 5 or 6 or 7 or 8 or 9 or 10 or 11 or 12 or 58 or 59 or 60 or 61 or 62 or 63 or 64 or 65 or 66 or 67 or 68 or 69

319. 13 or 14 or 15 or 16 or 17 or 18 or 19 or 20 or 21 or 22 or 23 or 24 or 25 or 26 or 27 or 28 or 29 or 30 or 31 or 32 or 33 or 34 or 35 or 36 or 37 or 38 or 39 or 40 or 41 or 42 or 43 or 44 or 45 or 46 or 47 or 48 or 49 or 50 or 51 or 52 or 53 or 54 or 55 or 56 or 57 or 70 or 71 or 72 or 73 or 74 or 75 or 76 or 77 or 78 or 79 or 80 or 81 or 82 or 83 or 84 or 85 or 86 or 87 or 88 or 89 or 90 or 91 or 92 or 93 or 94 or 95 or 96 or 97 or 98 or 99 or 100 or 101 or 102 or 103 or 104 or 105 or 106 or 107 or 108 or 109 or 110 or 111 or 112 or 113 or 114 or 115 or 116 or 117 or 118 or 119 or 120 or 121 or 122 or 123 or 124 or 125 or 126 or 127 or 128 or 129 or 130 or 131 or 132 or 133 or 134 or 135 or 136 or 137 or 138 or 139 or 140 or 141 or 142 or 143 or 144 or 145 or 146 or 147 or 148 or 149 or 150 or 151 or 152 or 153 or 154 or 155 or 156 or 157 or 158 or 159 or 160 or 161 or 162 or 163 or 164 or 165 or 166 or 167 or 168 or 169 or 170 or 171 or 172 or 173 or 174 or 175 or 176 or 177 or 178 or 179 or 180 or 181 or 182 or 183 or 184 or 185 or 186 or 187 or 188 or 189 or 190 or 191 or 192 or 193 or 194 or 195 or 196 or 197 or 198 or 199 or 200 or 201 or 202 or 203 or 204 or 205 or 206 or 207 or 208 or 209 or 210 or 211 or 212 or 213 or 214 or 215 or 216 or 217 or 218 or 219 or 220 or 221 or 222 or 223 or 224 or 225 or 226 or 227 or 228 or 229 or 230 or 231 or 232 or 233 or 234 or 235 or 236 or 237 or 238 or 239 or 240 or 241 or 242 or 243 or 244 or 245 or 246 or 247 or 248 or 249 or 250 or 251 or 252 or 253 or 254 or 255 or 256 or 257

320. 306 or 307 or 308 or 309 or 310 or 311 or 312 or 313 or 314 or 315 or 316 or 317

321. 258 or 259 or 260 or 261 or 262 or 263 or 264 or 265 or 266 or 267 or 268 or 269 or 270 or 271 or 272 or 273 or 274 or 275 or 276 or 277 or 278 or 279 or 280 or 281 or 282 or 283 or 284 or 285 or 286 or 287 or 288 or 289 or 290 or 291 or 292 or 293 or 294 or 295 or 296 or 297 or 298 or 299 or 300 or 301 or 302 or 303 or 304 or 305

322. 318 and 319 and 320 and 321

323. prescription commit.mp.

324. prescription commission.mp.

325. label* error.mp.

326. label* problem.mp.

327. label* concern.mp.

328. label* quality.mp.

329. label discrepancy.mp.

330. inappropriate label*.mp.

331. improper label*.mp.

332. label* mistake.mp.

333. label violation.mp.

334. label* omission.mp.

335. label* omit.mp.

336. label* commit.mp.

337. label* commission.mp.

338. label* mishap.mp.

339. incorrect label*.mp.

340. wrong label*.mp.

341. label* misadventure.mp.

342. label* oversight.mp.

343. label* incident.mp.

344. monitor* error.mp.

345. Monitor* Safe*.mp.

346. monitor* error.mp.

347. monitor* harm.mp.

348. monitor* risk.mp.

349. monitor* event.mp.

350. monitor* problem.mp.

351. monitor* incident.mp.

352. monitor* concern.mp.

353. monitor* quality.mp.

354. inappropriate monitor*.mp.

355. monitor* manag*.mp.

356. monitor* mistake.mp.

357. monitor* violation.mp.

358. monitor* omission.mp.

359. monitor* omit.mp.

360. monitor* commission.mp.

361. monitor* commit.mp.

362. monitor* duplicat*.mp.

363. monitor* mishap.mp.

364. improper monitor*.mp.

365. wrong monitor*.mp.

366. incorrect monitor*.mp.

367. monitor* misadventure.mp.

368. monitor* oversight.mp.

369. 323 or 324 or 325 or 326 or 327 or 328 or 329 or 330 or 331 or 332 or 333 or 334 or 335 or 336 or 337 or 338 or 339 or 340 or 341 or 342 or 343 or 344 or 345 or 346 or 347 or 348 or 349 or 350 or 351 or 352 or 353 or 354 or 355 or 356 or 357 or 358 or 359 or 360 or 361 or 362 or 363 or 364 or 365 or 366 or 367 or 368

370. 319 or 369

371. field work.mp.

372. 320 or 371

373. opinion.mp.

374. exp "Attitude of Health Personnel"/

375. grounded theory.mp. or exp Grounded Theory/

376. view.mp.

377. exp Observation/

378. 372 or 373 or 374 or 375 or 376 or 377

379. 318 and 321 and 370 and 378

ISI Web of Science

| Topic | (community OR private OR outpatient OR ambulatory OR retail OR store OR shop) **AND** (pharmacy OR pharmacist) |
| --- | --- |
| Topic | (drug OR medication OR therapy OR prescribing OR dispensing OR transcribing OR administering OR administration OR prescription OR route OR dose OR dosing OR overprescribe OR calculation OR monitor) AND (safety OR error OR harm OR risk OR event OR problem OR incident OR concern OR quality OR interaction or discrepancy OR inappropriate OR intervention OR management OR mistake OR violation OR omission OR commission OR duplication OR mishap OR improper OR wrong OR incorrect OR misadventure OR oversight) |
| Topic | (Qualitative OR mixed OR focus group OR ethnograph* OR anthropology* OR interview OR theme OR thematic OR code OR quote OR field note OR observation OR field work OR opinion OR view OR grounded theory) |
| Topic | (Cause OR factor OR human OR work OR stress OR burnout OR organization* OR system OR element OR origin OR root OR provocation OR source OR incentive OR occasion OR explanation OR catalyst OR stimulus OR trigger OR risk factor OR origin OR ergonomics OR sociotechnical OR motive OR motivation OR goal OR impetus OR rationale OR basis OR ground OR aetiology OR reason OR engineer* OR interface OR component OR aspect  OR variable OR influence OR determinant OR contributor OR parameter OR feature OR challenge OR barrier OR obstacle OR facilitator) |

Database of Abstracts of Reviews of Effects (DARE) and Global Health Database

| Title | (community OR private OR outpatient OR ambulatory OR retail OR store OR shop) **AND** (pharmacy OR pharmacist) |
| --- | --- |
| Title | (drug OR medication OR therapy OR prescribing OR dispensing OR transcribing OR administering OR administration OR prescription OR route OR dose OR dosing OR overprescribe OR calculation OR monitor) AND (safety OR error OR harm OR risk OR event OR problem OR incident OR concern OR quality OR interaction or discrepancy OR inappropriate OR intervention OR management OR mistake OR violation OR omission OR commission OR duplication OR mishap OR improper OR wrong OR incorrect OR misadventure OR oversight) |
| Title | (Cause OR factor OR human OR work OR stress OR burnout OR organization* OR system OR element OR origin OR root OR provocation OR source OR incentive OR occasion OR explanation OR catalyst OR stimulus OR trigger OR risk factor OR origin OR ergonomics OR sociotechnical OR motive OR motivation OR goal OR impetus OR rationale OR basis OR ground OR aetiology OR reason OR engineer* OR interface OR component OR aspect  OR variable OR influence OR determinant OR contributor OR parameter OR feature OR challenge OR barrier OR obstacle OR facilitator) |

Scopus

| Abstract, title,keyword | (community OR private OR outpatient OR ambulatory OR retail OR store OR shop) **AND** (pharmacy OR pharmacist) |
| --- | --- |
| Abstract, title,keyword | (drug OR medication OR therapy OR prescribing OR dispensing OR transcribing OR administering OR administration OR prescription OR route OR dose OR dosing OR overprescribe OR calculation OR monitor) AND (safety OR error OR harm OR risk OR event OR problem OR incident OR concern OR quality OR interaction OR discrepancy OR inappropriate OR intervention OR management OR mistake OR violation OR omission OR commission OR duplication OR mishap OR improper OR wrong OR incorrect OR misadventure OR oversight) |
| Abstract, title,keyword | (Qualitative OR mixed OR focus group OR ethnograph* OR anthropology* OR interview OR theme OR thematic OR code OR quote OR field note OR observation OR field work OR opinion OR view OR grounded theory) |
| Abstract, title,keyword | (Cause OR factor OR human OR work OR stress OR burnout OR organization* OR system OR element OR origin OR root OR provocation OR source OR incentive OR occasion OR explanation OR catalyst OR stimulus OR trigger OR risk factor OR origin OR ergonomics OR sociotechnical OR motive OR motivation OR goal OR impetus OR rationale OR basis OR ground OR aetiology OR reason OR engineer* OR interface OR component OR aspect  OR variable OR influence OR determinant OR contributor OR parameter OR feature OR challenge OR barrier OR obstacle OR facilitator) |

ScienceDirect (Elsevier)

Title, abstract or author-specified keywords (pharmacy OR pharmacist) AND (medication error OR drug error OR medication safety OR drug safety)

Google Scholar

1. ((community OR private OR outpatient OR ambulatory OR retail OR store OR shop) AND (pharmacy OR pharmacist)) AND ((drug OR medication OR therapy OR prescribing OR dispensing OR transcribing OR administering OR administration OR prescription OR route OR dose OR dosing OR overprescribe OR calculation OR monitor) AND (safety OR error OR harm OR risk OR event OR problem OR incident OR concern OR quality OR interaction OR discrepancy OR inappropriate OR intervention OR management OR mistake OR violation OR omission OR commission OR duplication OR mishap OR improper OR wrong OR incorrect OR misadventure OR oversight)) AND ((Qualitative OR mixed OR focus group OR ethnograph* OR anthropology* OR interview OR theme OR thematic OR code OR quote OR field note OR observation OR field work OR opinion OR view OR grounded theory)) AND ((Cause OR factor OR human OR work OR stress OR burnout OR organization* OR system OR element OR origin OR root OR provocation OR source OR incentive OR occasion OR explanation OR catalyst OR stimulus OR trigger OR risk factor OR origin OR ergonomics OR sociotechnical OR motive OR motivation OR goal OR impetus OR rationale OR basis OR ground OR aetiology OR reason OR engineer* OR interface OR component OR aspect OR variable OR influence OR determinant OR contributor OR parameter OR feature OR challenge OR barrier OR obstacle OR facilitator)) (25 pages are extracted)
2. ((community OR private OR outpatient OR ambulatory OR retail OR store OR shop) AND (pharmacy OR pharmacist)) AND ((drug OR medication) AND (safety OR error OR harm OR risk OR event OR problem OR incident OR concern OR quality)) AND ((Qualitative OR mixed OR focus group OR ethnograph* OR anthropology* OR interview OR theme OR thematic OR code OR quote OR field note OR observation OR field work OR opinion OR view OR grounded theory)) (25 pages are extracted)

Cumulative Index to Nursing and Allied Health Literature (CINAHL) (EBSCO)

| Abstract | (community OR private OR outpatient OR ambulatory OR retail OR store OR shop) **AND** (pharmacy OR pharmacist) |
| --- | --- |
| Abstract | (drug OR medication OR therapy OR prescribing OR dispensing OR transcribing OR administering OR administration OR prescription OR route OR dose OR dosing OR overprescribe OR calculation OR monitor) AND (safe* OR error OR harm OR risk OR event OR problem OR incident OR concern OR quality OR interaction or discrepancy OR inappropriate OR intervention OR management OR mistake OR violation OR omission OR commission OR duplication OR mishap OR improper OR wrong OR incorrect OR misadventure OR oversight) |
| Abstract | (Qualitative OR mixed OR focus group OR ethnograph* OR anthropology* OR interview OR theme OR thematic OR code OR quote OR field note OR observation OR field work OR opinion OR view OR grounded theory) |
| Abstract | (Cause OR factor OR human OR work OR stress OR burnout OR organization* OR system OR element OR origin OR root OR provocation OR source OR incentive OR occasion OR explanation OR catalyst OR stimulus OR trigger OR risk factor OR origin OR ergonomics OR sociotechnical OR motive OR motivation OR goal OR impetus OR rationale OR basis OR ground OR aetiology OR reason OR engineer* OR interface OR component OR aspect  OR variable OR influence OR determinant OR contributor OR parameter OR feature OR challenge OR barrier OR obstacle OR facilitator) |

Cochrane Central Register of Controlled Trials

| Title abstract keyword | (community OR private OR outpatient OR ambulatory OR retail OR store OR shop) **AND** (pharmacy OR pharmacist) |
| --- | --- |
| Title abstract keyword | (drug OR medication OR therapy OR prescribing OR dispensing OR transcribing OR administering OR administration OR prescription OR route OR dose OR dosing OR overprescribe OR calculation OR monitor) AND (safe* OR error OR harm OR risk OR event OR problem OR incident OR concern OR quality OR interaction or discrepancy OR inappropriate OR intervention OR management OR mistake OR violation OR omission OR commission OR duplication OR mishap OR improper OR wrong OR incorrect OR misadventure OR oversight) |
| Title abstract keyword | (Qualitative OR mixed OR focus group OR ethnograph* OR anthropology* OR interview OR theme OR thematic OR code OR quote OR field note OR observation OR field work OR opinion OR view OR grounded theory) |
| Title abstract keyword | (Cause OR factor OR human OR work OR stress OR burnout OR organization* OR system OR element OR origin OR root OR provocation OR source OR incentive OR occasion OR explanation OR catalyst OR stimulus OR trigger OR risk factor OR origin OR ergonomics OR sociotechnical OR motive OR motivation OR goal OR impetus OR rationale OR basis OR ground OR aetiology OR reason OR engineer* OR interface OR component OR aspect  OR variable OR influence OR determinant OR contributor OR parameter OR feature OR challenge OR barrier OR obstacle OR facilitator) |

ProQuest Dissertations & Theses Global

| All abstract and summary text – summary | (community OR private OR outpatient OR ambulatory OR retail OR store OR shop) **AND** (pharmacy OR pharmacist) |
| --- | --- |
| All abstract and summary text – summary | (drug OR medication OR therapy OR prescribing OR dispensing OR transcribing OR administering OR administration OR prescription OR route OR dose OR dosing OR overprescribe OR calculation OR monitor) AND (safe* OR error OR harm OR risk OR event OR problem OR incident OR concern OR quality OR interaction or discrepancy OR inappropriate OR intervention OR management OR mistake OR violation OR omission OR commission OR duplication OR mishap OR improper OR wrong OR incorrect OR misadventure OR oversight) |
| All abstract and summary text – summary | (Qualitative OR mixed OR focus group OR ethnograph* OR anthropology* OR interview OR theme OR thematic OR code OR quote OR field note OR observation OR field work OR opinion OR view OR grounded theory) |
| All abstract and summary text – summary | (Cause OR factor OR human OR work OR stress OR burnout OR organization* OR system OR element OR origin OR root OR provocation OR source OR incentive OR occasion OR explanation OR catalyst OR stimulus OR trigger OR risk factor OR origin OR ergonomics OR sociotechnical OR motive OR motivation OR goal OR impetus OR rationale OR basis OR ground OR aetiology OR reason OR engineer* OR interface OR component OR aspect  OR variable OR influence OR determinant OR contributor OR parameter OR feature OR challenge OR barrier OR obstacle OR facilitator) |

Academic Search Ultimate

| Abstract or author supplied abstract | (community OR private OR outpatient OR ambulatory OR retail OR store OR shop) **AND** (pharmacy OR pharmacist) |
| --- | --- |
| Abstract or author supplied abstract | (drug OR medication OR therapy OR prescribing OR dispensing OR transcribing OR administering OR administration OR prescription OR route OR dose OR dosing OR overprescribe OR calculation OR monitor) AND (safe* OR error OR harm OR risk OR event OR problem OR incident OR concern OR quality OR interaction or discrepancy OR inappropriate OR intervention OR management OR mistake OR violation OR omission OR commission OR duplication OR mishap OR improper OR wrong OR incorrect OR misadventure OR oversight) |
| Abstract or author supplied abstract | (Qualitative OR mixed OR focus group OR ethnograph* OR anthropology* OR interview OR theme OR thematic OR code OR quote OR field note OR observation OR field work OR opinion OR view OR grounded theory) |
| Abstract or author supplied abstract | (Cause OR factor OR human OR work OR stress OR burnout OR organization* OR system OR element OR origin OR root OR provocation OR source OR incentive OR occasion OR explanation OR catalyst OR stimulus OR trigger OR risk factor OR origin OR ergonomics OR sociotechnical OR motive OR motivation OR goal OR impetus OR rationale OR basis OR ground OR aetiology OR reason OR engineer* OR interface OR component OR aspect  OR variable OR influence OR determinant OR contributor OR parameter OR feature OR challenge OR barrier OR obstacle OR facilitator) |

Health Systems Evidence

((community OR private OR outpatient OR ambulatory OR retail OR store OR shop) AND (pharmacy OR pharmacist)) AND ((drug OR medication OR therapy OR prescribing OR dispensing OR transcribing OR administering OR administration OR prescription OR route OR dose OR dosing OR overprescribe OR calculation OR monitor) AND (safe* OR error OR harm OR risk OR event OR problem OR incident OR concern OR quality OR interaction or discrepancy OR inappropriate OR intervention OR management OR mistake OR violation OR omission OR commission OR duplication OR mishap OR improper OR wrong OR incorrect OR misadventure OR oversight)) AND ((Qualitative OR mixed OR focus group OR ethnograph* OR anthropology* OR interview OR theme OR thematic OR code OR quote OR field note OR observation OR field work OR opinion OR view OR grounded theory)) AND ((Cause OR factor OR human OR work OR stress OR burnout OR organization* OR system OR element OR origin OR root OR provocation OR source OR incentive OR occasion OR explanation OR catalyst OR stimulus OR trigger OR risk factor OR origin OR ergonomics OR sociotechnical OR motive OR motivation OR goal OR impetus OR rationale OR basis OR ground OR aetiology OR reason OR engineer* OR interface OR component OR aspect OR variable OR influence OR determinant OR contributor OR parameter OR feature OR challenge OR barrier OR obstacle OR facilitator))

Embase (Ovid)

1. community pharmacy.mp. or exp Pharmacies/

2. community pharmacist.mp. or exp Pharmacists/

3. exp Pharmacies/ or private pharmacy.mp.

4. exp Pharmacists/ or exp Community Pharmacy Services/ or private pharmacist.mp. or exp Pharmacies/

5. exp Ambulatory Care/ or outpatient pharmacy.mp.

6. outpatient pharmacist.mp.

7. ambulatory pharmacy.mp.

8. ambulatory pharmacist.mp.

9. retail pharmacy.mp.

10. retail pharmacist.mp. or exp Pharmacists/

11. pharmacy store.mp.

12. pharmacy shop.mp.

13. exp Medication Errors/ or drug error.mp.

14. drug safe*.mp.

15. exp Harm Reduction/ or drug harm.mp.

16. drug risk.mp.

17. drug event.mp.

18. drug problem.mp.

19. drug incident.mp.

20. drug concern.mp.

21. drug quality.mp.

22. drug interaction.mp. or exp Drug Interactions/

23. drug discrepancy.mp.

24. exp Inappropriate Prescribing/ or inappropriate drug.mp.

25. drug intervention.mp.

26. drug manag*.mp.

27. drug mistake.mp.

28. drug violation.mp.

29. drug omission.mp.

30. drug omit.mp.

31. drug comission.mp.

32. drug commit.mp.

33. drug mishap.mp.

34. improper drug.mp.

35. wrong drug.mp.

36. incorrect drug.mp.

37. nearmiss.mp.

38. drug misadventure.mp.

39. drug oversight.mp.

40. medication safe*.mp.

41. medication safety.mp.

42. medication harm.mp.

43. medication risk.mp.

44. medication event.mp.

45. medication problem.mp.

46. medication incident.mp.

47. medication concern.mp.

48. medication quality.mp.

49. medication interaction.mp

50. medication discrepancy.mp.

51. inappropriate medication.mp.

52. medication intervention.mp.

53. medication management.mp.

54. medication mistake.mp.

55. medication violation.mp.

56. medication omission.mp.

57. medication commission.mp.

58. community pharmacy.mp. or exp Pharmacies/

59. community pharmacist.mp. or exp Pharmacists/

60. exp Pharmacies/ or private pharmacy.mp.

61. exp Pharmacists/ or exp Community Pharmacy Services/ or private pharmacist.mp. or exp Pharmacies/

62. exp Ambulatory Care/ or outpatient pharmacy.mp.

63. outpatient pharmacist.mp.

64. ambulatory pharmacy.mp.

65. ambulatory pharmacist.mp.

66. retail pharmacy.mp.

67. retail pharmacist.mp. or exp Pharmacists/

68. pharmacy store.mp.

69. pharmacy shop.mp.

70. exp Medication Errors/ or drug error.mp.

71. drug safe*.mp.

72. exp Harm Reduction/ or drug harm.mp.

73. drug risk.mp.

74. drug event.mp.

75. drug problem.mp.

76. drug incident.mp.

77. drug concern.mp.

78. drug interaction.mp. or exp Drug Interactions/

79. drug discrepancy.mp.

80. drug intervention.mp.

81. drug manag*.mp.

82. drug mistake.mp.

83. drug violation.mp.

84. drug omission.mp.

85. drug commit.mp.

86. drug mishap.mp.

87. improper drug.mp.

88. wrong drug.mp.

89. incorrect drug.mp.

90. nearmiss.mp.

91. drug misadventure.mp.

92. drug oversight.mp.

93. medication safe*.mp.

94. medication safety.mp.

95. medication harm.mp.

96. medication risk.mp.

97. medication event.mp.

98. medication problem.mp.

99. medication incident.mp.

100. medication concern.mp.

101. medication quality.mp.

102. medication interaction.mp.

103. medication discrepancy.mp.

104. inappropriate medication.mp.

105. medication intervention.mp.

106. medication management.mp.

107. medication mistake.mp.

108. medication violation.mp.

109. medication omission.mp.

110. medication commission.mp.

111. medication mishap.mp.

112. improper medication.mp.

113. wrong medication.mp.

114. incorrect medication.mp.

115. nearmiss.mp.

116. medication misadventure.mp.

117. medication oversight.mp.

118. safe therapy.mp.

119. therapy error.mp.

120. therapy risk.mp.

121. therapy event.mp.

122. therapy harm.mp.

123. therapy problem.mp.

124. therapy incident.mp.

125. therapy concern.mp.

126. therapy quality.mp.

127. therapy interaction.mp.

128. therapy discrepancy.mp.

129. Inappropriate therapy.mp.

130. therapy management.mp.

131. therapy mistake.mp.

132. therapy violation.mp.

133. therapy omission.mp.

134. therapy omit.mp.

135. therapy commission.mp.

136. therapy commit.mp.

137. therapy mishap.mp.

138. medication duplication.mp.

139. therapy duplication.mp.

140. drug duplication.mp.

141. therapy mishap.mp.

142. improper therapy.mp.

143. wrong therapy.mp.

144. incorrect therapy.mp.

145. therapy misadventure.mp.

146. therapy oversight.mp.

147. prescribing error.mp.

148. prescription error.mp.

149. prescribing harm.mp.

150. prescription harm.mp.

151. prescription risk.mp.

152. prescribing risk.mp.

153. prescribing problem.mp.

154. prescription problem.mp.

155. prescribing incident.mp.

156. prescription incident.mp.

157. prescription concern.mp.

158. prescribing concern.mp.

159. prescription quality.mp.

160. prescribing quality.mp.

161. prescription discrepancy.mp.

162. prescribing discrepancy.mp.

163. inappropriate prescription.mp. or exp Inappropriate Prescribing/

164. improper prescribing.mp.

165. improper prescription.mp.

166. prescribing intervention.mp.

167. prescription intervention.mp.

168. prescribing mistake.mp.

169. prescription mistake.mp.

170. prescription violation.mp.

171. prescribing violation.mp.

172. prescription mishap.mp.

173. prescribing mishap.mp.

174. wrong prescription.mp.

175. wrong prescribing.mp.

176. incorrect prescription.mp.

177. incorrect prescribing.mp.

178. prescription misadventure.mp.

179. prescribing misadventure.mp.

180. dispensing error.mp.

181. dispensing harm.mp.

182. dispensing risk.mp.

183. dispensing event.mp.

184. dispensing problem.mp.

185. dispensing incident.mp.

186. dispensing concern.mp.

187. dispensing quality.mp.

188. dispensing discrepancy.mp.

189. inappropriate dispensing.mp.

190. dispensing mistake.mp.

191. dispensing violation.mp.

192. dispensing omission.mp.

193. dispensing commission.mp.

194. dispensing duplication.mp.

195. dispensing mishap.mp.

196. improper dispensing.mp.

197. wrong dispensing.mp.

198. incorrect dispensing.mp.

199. dispensing misadventure.mp.

200. dispensing oversight.mp.

201. transcribing error.mp.

202. transcribing harm.mp.

203. transcribing risk.mp.

204. transcribing event.mp.

205. transcribing problem.mp.

206. transcribing incident.mp.

207. transcribing concern.mp.

208. transcribing quality.mp.

209. transcribing discrepancy.mp.

210. inappropriate transcribing.mp.

211. improper transcribing.mp.

212. transcribing mistake.mp.

213. transcribing duplication.mp.

214. wrong transcribing.mp.

215. incorrect transcribing.mp.

216. transcribing misadventure.mp.

217. administration error.mp.

218. administration harm.mp.

219. administration event.mp.

220. administration risk.mp.

221. administration problem.mp.

222. administration incident.mp.

223. administration concern.mp.

224. administration discrepancy.mp.

225. inappropriate administration.mp.

226. administration mistake.mp.

227. administration violation.mp.

228. administration omission.mp.

229. administration commission.mp.

230. administration duplication.mp.

231. administration mishap.mp.

232. improper administration.mp.

233. wrong administration.mp.

234. incorrect administration.mp.

235. administration misadventure.mp.

236. administration oversight.mp.

237. inappropriate route of administration.mp.

238. wrong route of administration.mp.

239. incorrect route of administration.mp.

240. improper route of administration.mp.

241. wrong dose.mp.

242. incorrect dose.mp.

243. inappropriate dose.mp.

244. improper dose.mp.

245. dose duplication.mp.

246. dose commission.mp.

247. dose omission.mp.

248. overprescribing.mp.

249. error in calculation.mp.

250. inappropriate calculation.mp.

251. improper calculation.mp.

252. wrong calculation.mp.

253. calculation problem.mp.

254. exp "Root Cause Analysis"/ or Cause.mp.

255. factor.mp. or exp Time Factors/

256. human.mp. or exp Humans/

257. work/ or "personnel staffing and scheduling"/

258. work.mp.

259. exp Financial Stress/

260. stress.mp. or exp Occupational Stress/ or exp Stress, Psychological/

261. exp Burnout, Psychological/ or exp Burnout, Professional/ or burnout.mp.

262. system factor.mp.

263. system.mp.

264. element.mp. or exp Elements/

265. organization*.mp.

266. origin.mp.

267. root.mp.

268. provocation.mp.

269. source.mp.

270. incentive.mp. or exp Motivation/

271. occasion.mp.

272. explanation.mp.

273. catalyst.mp.

274. stimulus.mp. or exp Generalization, Stimulus/

275. trigger.mp.

276. risk factor.mp. or exp Risk Factors/

277. origin.mp.

278. Ergonomics.mp. or exp Ergonomics/

279. systems analysis/ or systems integration/ or workflow/

280. motive.mp.

281. goal.mp. or exp Goals/

282. impetus.mp.

283. rationale.mp.

284. basis.mp.

285. ground.mp.

286. etiology.mp.

287. reason.mp.

288. engineer*.mp.

289. interface.mp.

290. component.mp.

291. aspect.mp.

292. variable.mp.

293. influence.mp.

294. determinant.mp.

295. contributor.mp.

296. parameter.mp.

297. feature.mp.

298. challenge.mp.

299. barrier.mp.

300. obstacle.mp.

301. facilitator.mp.

302. qualitative design.mp. or exp Interviews as Topic/ or Qualitative Research/

303. mixed methods.mp.

304. focus group.mp. or exp Focus Groups/

305. ethnograph*.mp.

306. exp Anthropology, Cultural/ or exp Anthropology/ or anthropology*.mp.

307. exp Interview, Psychological/ or exp Interview/ or interview.mp.

308. theme.mp.

309. thematic.mp.

310. code.mp.

311. quote.mp.

312. field note.mp.

313. observation.mp. or exp Observation/

314. exp drug safety/

315. drug quality.mp. or exp drug quality/

316. drug omit*.mp.

317. drug commission.mp.

318. drug commit*.mp.

319. drug duplicat*.mp.

320. exp inappropriate prescribing/ or inappropriate drug.mp. or exp potentially inappropriate medication/

321. medication manag*.mp.

322. medication omit*.mp.

323. medication commit*.mp.

324. medication duplicat*.mp.

325. exp prescribing error/

326. exp prescribing error/

327. exp dispensing error/

328. Administer* error.mp.

329. Administering error.mp.

330. transcrib* error.mp.

331. monitor* error.mp.

332. therapy error.mp. or exp therapeutic error/

333. therapy omit*.mp.

334. therapy commit*.mp.

335. therapy commission.mp.

336. therapy duplicat*.mp.

337. prescrib* harm.mp.

338. prescrib* risk.mp.

339. prescrib* event.mp.

340. prescrib* incident.mp.

341. prescrib* problem.mp.

342. prescrib* quality.mp.

343. prescrib* interaction.mp.

344. prescrib* discrepancy.mp.

345. prescrib* inappropriate.mp.

346. prescrib* intervention.mp.

347. prescrib* omission.mp.

348. prescrib* duplicat*.mp.

349. improper prescrib*.mp.

350. wrong prescribing.mp.

351. wrong prescription.mp.

352. incorrect prescribing.mp.

353. incorrect prescription.mp.

354. prescribing misadventure.mp.

355. prescription misadventure.mp.

356. prescrib* oversight.mp.

357. prescription oversight.mp.

358. dispensing management.mp.

359. dispensing violation.mp.

360. dispensing omit*.mp.

361. dispensing commit.mp.

362. dispensing commission.mp.

363. dispensing duplication.mp.

364. dispensing mishap.mp.

365. Administration omit.mp.

366. administration commit.mp.

367. administration commission.mp.

368. administer* error.mp.

369. administer* event.mp.

370. administer* risk.mp.

371. administer* harm.mp.

372. administer* incident.mp.

373. improper administer*.mp.

374. incorrect administer*.mp.

375. administer* discrepancy.mp.

376. administer* omission.mp.

377. administer* commission.mp.

378. administer* omit.mp.

379. administer* commit.mp.

380. administer* problem.mp.

381. administer* duplication.mp.

382. administer* violation.mp.

383. administer* mishap.mp.

384. administer* oversight.mp.

385. administer* incident.mp.

386. administer* concern.mp.

387. administer* quality.mp.

388. administer* management.mp.

389. administer* intervention.mp.

390. monitor* Safe*.mp.

391. monitor* error.mp.

392. monitor* harm.mp.

393. monitor* risk.mp.

394. Monitor* event.mp.

395. Monitor* problem.mp.

396. Monitor* incident.mp.

397. Monitor* concern.mp.

398. monitor* quality.mp.

399. monitor* interaction.mp.

400. Monitor* discrepancy.mp.

401. inappropriate Monitor*.mp.

402. Monitor* intervention.mp.

403. monitor* mistake.mp.

404. monitor* violation.mp.

405. Monitor* omission.mp.

406. monitor* omit.mp.

407. monitor* commission.mp.

408. monitor* commit.mp.

409. monitor* mishap.mp.

410. improper monitor*.mp.

411. wrong monitor*.mp.

412. wrong monitor*.mp.

413. incorrect monitor*.mp.

414. monitor* misadventure.mp.

415. monitor* oversight.mp.

416. community pharmacy.mp. or exp Pharmacies/

417. community pharmacist.mp. or exp Pharmacists/

418. exp Pharmacies/ or private pharmacy.mp.

419. exp Pharmacists/ or exp Community Pharmacy Services/ or private pharmacist.mp. or exp Pharmacies/

420. exp Ambulatory Care/ or outpatient pharmacy.mp.

421. outpatient pharmacist.mp.

422. ambulatory pharmacy.mp.

423. ambulatory pharmacist.mp.

424. retail pharmacy.mp.

425. retail pharmacist.mp. or exp Pharmacists/

426. pharmacy store.mp.

427. pharmacy shop.mp.

428. exp Medication Errors/ or drug error.mp.

429. drug safe*.mp.

430. exp Harm Reduction/ or drug harm.mp.

431. drug risk.mp.

432. drug event.mp.

433. drug problem.mp.

434. drug incident.mp.

435. drug concern.mp.

436. drug quality.mp.

437. drug interaction.mp. or exp Drug Interactions/

438. drug discrepancy.mp.

439. exp Inappropriate Prescribing/ or inappropriate drug.mp.

440. drug intervention.mp.

441. drug manag*.mp.

442. drug mistake.mp.

443. drug violation.mp.

444. drug omission.mp.

445. drug omit.mp.

446. drug comission.mp.

447. drug commit.mp.

448. drug mishap.mp.

449. improper drug.mp.

450. wrong drug.mp.

451. incorrect drug.mp.

452. nearmiss.mp.

453. drug misadventure.mp.

454. drug oversight.mp.

455. medication safe*.mp.

456. medication safety.mp.

457. medication harm.mp.

458. medication risk.mp.

459. medication event.mp.

460. medication problem.mp.

461. medication incident.mp.

462. medication concern.mp.

463. medication quality.mp.

464. medication interaction.mp.

465. medication discrepancy.mp.

466. inappropriate medication.mp.

467. medication intervention.mp.

468. medication management.mp.

469. medication mistake.mp.

470. medication violation.mp.

471. medication omission.mp.

472. medication commission.mp.

473. community pharmacy.mp. or exp Pharmacies/

474. community pharmacist.mp. or exp Pharmacists/

475. exp Pharmacies/ or private pharmacy.mp.

476. exp Pharmacists/ or exp Community Pharmacy Services/ or private pharmacist.mp. or exp Pharmacies/

477. exp Ambulatory Care/ or outpatient pharmacy.mp.

478. outpatient pharmacist.mp.

479. ambulatory pharmacy.mp.

480. ambulatory pharmacist.mp.

481. retail pharmacy.mp.

482. retail pharmacist.mp. or exp Pharmacists/

483. pharmacy store.mp.

484. pharmacy shop.mp.

485. exp Medication Errors/ or drug error.mp.

486. drug safe*.mp.

487. exp Harm Reduction/ or drug harm.mp.

488. drug risk.mp.

489. drug event.mp.

490. drug problem.mp.

491. drug incident.mp.

492. drug concern.mp.

493. drug interaction.mp. or exp Drug Interactions/

494. drug discrepancy.mp.

495. drug intervention.mp.

496. drug manag*.mp.

497. drug mistake.mp.

498. drug violation.mp.

499. drug omission.mp.

500. drug commit.mp.

501. drug mishap.mp.

502. improper drug.mp.

503. wrong drug.mp.

504. incorrect drug.mp.

505. nearmiss.mp.

506. drug misadventure.mp.

507. drug oversight.mp.

508. medication safe*.mp.

509. medication safety.mp.

510. medication harm.mp.

511. medication risk.mp.

512. medication event.mp.

513. medication problem.mp.

514. medication incident.mp.

515. medication concern.mp.

516. medication quality.mp.

517. medication interaction.mp.

518. medication discrepancy.mp.

519. inappropriate medication.mp.

520. medication intervention.mp.

521. medication management.mp.

522. medication mistake.mp.

523. medication violation.mp.

524. medication omission.mp.

525. medication commission.mp.

526. medication mishap.mp.

527. improper medication.mp.

528. wrong medication.mp.

529. incorrect medication.mp.

530. nearmiss.mp.

531. medication misadventure.mp.

532. medication oversight.mp.

533. safe therapy.mp.

534. therapy error.mp.

535. therapy risk.mp.

536. therapy event.mp.

537. therapy harm.mp.

538. therapy problem.mp.

539. therapy incident.mp.

540. therapy concern.mp.

541. therapy quality.mp.

542. therapy interaction.mp.

543. therapy discrepancy.mp.

544. Inappropriate therapy.mp.

545. therapy management.mp.

546. therapy mistake.mp.

547. therapy violation.mp.

548. therapy omission.mp.

549. therapy omit.mp.

550. therapy commission.mp.

551. therapy commit.mp.

552. therapy mishap.mp.

553. medication duplication.mp.

554. therapy duplication.mp.

555. drug duplication.mp.

556. therapy mishap.mp.

557. improper therapy.mp.

558. wrong therapy.mp.

559. incorrect therapy.mp.

560. therapy misadventure.mp.

561. therapy oversight.mp.

562. prescribing error.mp.

563. prescription error.mp.

564. prescribing harm.mp.

565. prescription harm.mp.

566. prescription risk.mp.

567. prescribing risk.mp.

568. prescribing problem.mp.

569. prescription problem.mp.

570. prescribing incident.mp.

571. prescription incident.mp.

572. prescription concern.mp.

573. prescribing concern.mp.

574. prescription quality.mp.

575. prescribing quality.mp.

576. prescription discrepancy.mp.

577. prescribing discrepancy.mp.

578. inappropriate prescription.mp. or exp Inappropriate Prescribing/

579. improper prescribing.mp.

580. improper prescription.mp.

581. prescribing intervention.mp.

582. prescription intervention.mp.

583. prescribing mistake.mp.

584. prescription mistake.mp.

585. prescription violation.mp.

586. prescribing violation.mp.

587. prescription mishap.mp.

588. prescribing mishap.mp.

589. wrong prescription.mp.

590. wrong prescribing.mp.

591. incorrect prescription.mp.

592. incorrect prescribing.mp.

593. prescription misadventure.mp.

594. prescribing misadventure.mp.

595. dispensing error.mp.

596. dispensing harm.mp.

597. dispensing risk.mp.

598. dispensing event.mp.

599. dispensing problem.mp.

600. dispensing incident.mp.

601. dispensing concern.mp.

602. dispensing quality.mp.

603. dispensing discrepancy.mp.

604. inappropriate dispensing.mp.

605. dispensing mistake.mp.

606. dispensing violation.mp.

607. dispensing omission.mp.

608. dispensing commission.mp.

609. dispensing duplication.mp.

610. dispensing mishap.mp.

611. improper dispensing.mp.

612. wrong dispensing.mp.

613. incorrect dispensing.mp.

614. dispensing misadventure.mp.

615. dispensing oversight.mp.

616. transcribing error.mp.

617. transcribing harm.mp.

618. transcribing risk.mp.

619. transcribing event.mp.

620. transcribing problem.mp.

621. transcribing incident.mp.

622. transcribing concern.mp.

623. transcribing quality.mp.

624. transcribing discrepancy.mp.

625. inappropriate transcribing.mp.

626. improper transcribing.mp.

627. transcribing mistake.mp.

628. transcribing duplication.mp.

629. wrong transcribing.mp.

630. incorrect transcribing.mp.

631. transcribing misadventure.mp.

632. administration error.mp.

633. administration harm.mp.

634. administration event.mp.

635. administration risk.mp.

636. administration problem.mp.

637. administration incident.mp.

638. administration concern.mp.

639. administration discrepancy.mp.

640. inappropriate administration.mp.

641. administration mistake.mp.

642. administration violation.mp.

643. administration omission.mp.

644. administration commission.mp.

645. administration duplication.mp.

646. administration mishap.mp.

647. improper administration.mp.

648. wrong administration.mp.

649. incorrect administration.mp.

650. administration misadventure.mp.

651. administration oversight.mp.

652. inappropriate route of administration.mp.

653. wrong route of administration.mp.

654. incorrect route of administration.mp.

655. improper route of administration.mp.

656. wrong dose.mp.

657. incorrect dose.mp.

658. inappropriate dose.mp.

659. improper dose.mp.

660. dose duplication.mp.

661. dose commission.mp.

662. dose omission.mp.

663. overprescribing.mp.

664. error in calculation.mp.

665. inappropriate calculation.mp.

666. improper calculation.mp.

667. wrong calculation.mp.

668. calculation problem.mp.

669. exp "Root Cause Analysis"/ or Cause.mp.

670. factor.mp. or exp Time Factors/

671. human.mp. or exp Humans/

672. work/ or "personnel staffing and scheduling"/

673. work.mp.

674. exp Financial Stress/

675. stress.mp. or exp Occupational Stress/ or exp Stress, Psychological/

676. exp Burnout, Psychological/ or exp Burnout, Professional/ or burnout.mp.

677. system factor.mp.

678. system.mp.

679. element.mp. or exp Elements/

680. organization*.mp.

681. origin.mp.

682. root.mp.

683. provocation.mp.

684. source.mp.

685. incentive.mp. or exp Motivation/

686. occasion.mp.

687. explanation.mp.

688. catalyst.mp.

689. stimulus.mp. or exp Generalization, Stimulus/

690. trigger.mp.

691. risk factor.mp. or exp Risk Factors/

692. origin.mp.

693. Ergonomics.mp. or exp Ergonomics/

694. systems analysis/ or systems integration/ or workflow/

695. motive.mp.

696. goal.mp. or exp Goals/

697. impetus.mp.

698. rationale.mp.

699. basis.mp.

700. ground.mp.

701. etiology.mp.

702. reason.mp.

703. engineer*.mp.

704. interface.mp.

705. component.mp.

706. aspect.mp.

707. variable.mp.

708. influence.mp.

709. determinant.mp.

710. contributor.mp.

711. parameter.mp.

712. feature.mp.

713. challenge.mp.

714. barrier.mp.

715. obstacle.mp.

716. facilitator.mp.

717. qualitative design.mp. or exp Interviews as Topic/ or Qualitative Research/

718. mixed methods.mp.

719. focus group.mp. or exp Focus Groups/

720. ethnograph*.mp.

721. exp Anthropology, Cultural/ or exp Anthropology/ or anthropology*.mp.

722. exp Interview, Psychological/ or exp Interview/ or interview.mp.

723. theme.mp.

724. thematic.mp.

725. code.mp.

726. quote.mp.

727. field note.mp.

728. observation.mp. or exp Observation/

729. exp drug safety/

730. drug quality.mp. or exp drug quality/

731. drug omit*.mp.

732. drug commission.mp.

733. drug commit*.mp.

734. drug duplicat*.mp.

735. exp inappropriate prescribing/ or inappropriate drug.mp. or exp potentially inappropriate medication/

736. medication manag*.mp.

737. medication omit*.mp.

738. medication commit*.mp.

739. medication duplicat*.mp.

740. exp prescribing error/

741. exp prescribing error/

742. exp dispensing error/

743. Administer* error.mp.

744. Administering error.mp.

745. transcrib* error.mp.

746. monitor* error.mp.

747. therapy error.mp. or exp therapeutic error/

748. therapy omit*.mp.

749. therapy commit*.mp.

750. therapy commission.mp.

751. therapy duplicat*.mp.

752. prescrib* harm.mp.

753. prescrib* risk.mp.

754. prescrib* event.mp.

755. prescrib* incident.mp.

756. prescrib* problem.mp.

757. prescrib* quality.mp.

758. prescrib* interaction.mp.

759. prescrib* discrepancy.mp.

760. prescrib* inappropriate.mp.

761. prescrib* intervention.mp.

762. prescrib* omission.mp.

763. prescrib* duplicat*.mp.

764. improper prescrib*.mp.

765. wrong prescribing.mp.

766. wrong prescription.mp.

767. incorrect prescribing.mp.

768. incorrect prescription.mp.

769. prescribing misadventure.mp.

770. prescription misadventure.mp.

771. prescrib* oversight.mp.

772. prescription oversight.mp.

773. dispensing management.mp.

774. dispensing violation.mp.

775. dispensing omit*.mp.

776. dispensing commit.mp.

777. dispensing commission.mp.

778. dispensing duplication.mp.

779. dispensing mishap.mp.

780. Administration omit.mp.

781. administration commit.mp.

782. administration commission.mp.

783. administer* error.mp.

784. administer* event.mp.

785. administer* risk.mp.

786. administer* harm.mp.

787. administer* incident.mp.

788. improper administer*.mp.

789. incorrect administer*.mp.

790. administer* discrepancy.mp.

791. administer* omission.mp.

792. administer* commission.mp.

793. administer* omit.mp.

794. administer* commit.mp.

795. administer* problem.mp.

796. administer* duplication.mp.

797. administer* violation.mp.

798. administer* mishap.mp.

799. administer* oversight.mp.

800. administer* incident.mp.

801. administer* concern.mp.

802. administer* quality.mp.

803. administer* management.mp.

804. administer* intervention.mp.

805. monitor* Safe*.mp.

806. monitor* error.mp.

807. monitor* harm.mp.

808. monitor* risk.mp.

809. Monitor* event.mp.

810. Monitor* problem.mp.

811. Monitor* incident.mp.

812. Monitor* concern.mp.

813. monitor* quality.mp.

814. monitor* interaction.mp.

815. Monitor* discrepancy.mp.

816. inappropriate Monitor*.mp.

817. Monitor* intervention.mp.

818. monitor* mistake.mp.

819. monitor* violation.mp.

820. Monitor* omission.mp.

821. monitor* omit.mp.

822. monitor* commission.mp.

823. monitor* commit.mp.

824. monitor* mishap.mp.

825. improper monitor*.mp.

826. wrong monitor*.mp.

827. wrong monitor*.mp.

828. incorrect monitor*.mp.

829. monitor* misadventure.mp.

830. monitor* oversight.mp.

831. prescribing commit.mp.

832. prescription commit.mp.

833. prescription commission.mp.

834. prescription commit.mp.

835. label* error.mp.

836. label* problem.mp.

837. label* concern.mp.

838. label* quality.mp.

839. label* discrepancy.mp.

840. inappropriate label*.mp.

841. label* mistake.mp.

842. label* violation.mp.

843. label* omission.mp.

844. label* omit.mp.

845. label* commission.mp.

846. label* commit.mp.

847. label* mishap.mp.

848. improper label*.mp.

849. wrong label*.mp.

850. incorrect label*.mp.

851. label* misadventure.mp.

852. label* oversight.mp.

853. exp "pharmacy (shop)"/

854. outpatient pharmacy.mp. or exp pharmacy/

855. exp ambulatory care/ or exp pharmacist/ or ambulatory pharmacy.mp. or exp pharmacy/

856. retail pharmacy.mp.

857. pharmacy store.mp.

858. community pharmacist.mp. or exp community pharmacist/

859. factor.mp. or exp time factor/ or exp cultural factor/ or exp risk factor/

860. human.mp. or exp human factors research/

861. exp work experience/

862. stress.mp. or exp physiological stress/

863. exp professional burnout/ or exp burnout/ or burnout.mp.

864. exp organizational citizenship/ or exp organizational climate/ or exp organizational culture/ or exp safety climate/ or exp safety culture/ or exp organizational decision making/ or exp organizational development/ or exp organizational efficiency/ or exp organizational policy/

865. exp health care system/ or exp "root system"/ or system.mp. or exp medication system/ or exp metric system/

866. element.mp.

867. origin.mp.

868. exp "root cause analysis"/ or root.mp.

869. provocation.mp. or exp provocation/

870. source.mp.

871. incentive.mp. or exp incentive/ or exp social incentive/

872. occasion.mp.

873. explanation.mp.

874. explain*.mp.

875. catalyst.mp.

876. stimulus.mp.

877. trigger.mp.

878. risk factor.mp. or exp risk factor/

879. origin.mp.

880. Ergonomics.mp. or exp ergonomics/

881. exp systems theory/

882. exp motivation/ or motiv*.mp.

883. goal.mp.

884. impetus.mp.

885. rationale.mp.

886. basis.mp.

887. ground.mp.

888. etiology.mp. or exp etiology/

889. reason.mp.

890. engineer*.mp.

891. interface.mp.

892. component.mp.

893. exp psychological aspect/ or aspect.mp. or exp economic aspect/ or exp social aspect/

894. exp explanatory variable/ or exp predictor variable/ or exp independent variable/ or variable.mp.

895. influenc*.mp.

896. determinant.mp.

897. contribut*.mp.

898. parameter.mp. or exp parameters/

899. feature.mp.

900. challenge.mp.

901. barrier.mp.

902. obstacle.mp.

903. facilitator.mp.

904. exp qualitative research/ or exp qualitative analysis/ or Qualitative.mp.

905. exp interview/ or mixed method.mp.

906. focus group.mp.

907. exp ethnographic research/ or exp ethnography/ or ethnograph*.mp.

908. exp cultural anthropology/ or exp anthropology/ or anthropology.mp.

909. exp structured interview/ or exp telephone interview/ or exp audio interview/ or exp unstructured interview/ or interview.mp. or exp psychological interview/ or exp video interview/ or exp semi structured interview/ or exp interview/

910. theme.mp.

911. exp thematic analysis/ or thematic.mp.

912. code.mp.

913. quote.mp.

914. field note.mp.

915. exp field work/

916. exp participant observation/ or exp non participant observation/ or exp observation/ or observation.mp.

917. 13 or 14 or 15 or 16 or 17 or 18 or 19 or 20 or 21 or 22 or 23 or 24 or 25 or 26 or 27 or 28 or 29 or 30 or 31 or 32 or 33 or 34 or 35 or 36 or 37 or 38 or 39 or 40 or 41 or 42 or 43 or 44 or 45 or 46 or 47 or 48 or 49 or 50 or 51 or 52 or 53 or 54 or 55 or 56 or 57 or 70 or 71 or 72 or 73 or 74 or 75 or 76 or 77 or 78 or 79 or 80 or 81 or 82 or 83 or 84 or 85 or 86 or 87 or 88 or 89 or 90 or 91 or 92 or 93 or 94 or 95 or 96 or 97 or 98 or 99 or 100 or 101 or 102 or 103 or 104 or 105 or 106 or 107 or 108 or 109 or 110 or 111 or 112 or 113 or 114 or 115 or 116 or 117 or 118 or 119 or 120 or 121 or 122 or 123 or 124 or 125 or 126 or 127 or 128 or 129 or 130 or 131 or 132 or 133 or 134 or 135 or 136 or 137 or 138 or 139 or 140 or 141 or 142 or 143 or 144 or 145 or 146 or 147 or 148 or 149 or 150 or 151 or 152 or 153 or 154 or 155 or 156 or 157 or 158 or 159 or 160 or 161 or 162 or 163 or 164 or 165 or 166 or 167 or 168 or 169 or 170 or 171 or 172 or 173 or 174 or 175 or 176 or 177 or 178 or 179 or 180 or 181 or 182 or 183 or 184 or 185 or 186 or 187 or 188 or 189 or 190 or 191 or 192 or 193 or 194 or 195 or 196 or 197 or 198 or 199 or 200 or 201 or 202 or 203 or 204 or 205 or 206 or 207 or 208 or 209 or 210 or 211 or 212 or 213 or 214 or 215 or 216 or 217 or 218 or 219 or 220 or 221 or 222 or 223 or 224 or 225 or 226 or 227 or 228 or 229 or 230 or 231 or 232 or 233 or 234 or 235 or 236 or 237 or 238 or 239 or 240 or 241 or 242 or 243 or 244 or 245 or 246 or 247 or 248 or 249 or 250 or 251 or 252 or 253 or 314 or 315 or 316 or 317 or 318 or 319 or 320 or 321 or 322 or 323 or 324 or 325 or 326 or 327 or 328 or 329 or 330 or 331 or 332 or 333 or 334 or 335 or 336 or 337 or 338 or 339 or 340 or 341 or 342 or 343 or 344 or 345 or 346 or 347 or 348 or 349 or 350 or 351 or 352 or 353 or 354 or 355 or 356 or 357 or 358 or 359 or 360 or 361 or 362 or 363 or 364 or 365 or 366 or 367 or 368 or 369 or 370 or 371 or 372 or 373 or 374 or 375 or 376 or 377 or 378 or 379 or 380 or 381 or 382 or 383 or 384 or 385 or 386 or 387 or 388 or 389 or 390 or 391 or 392 or 393 or 394 or 395 or 396 or 397 or 398 or 399 or 400 or 401 or 402 or 403 or 404 or 405 or 406 or 407 or 408 or 409 or 410 or 411 or 412 or 413 or 414 or 415 or 428 or 429 or 430 or 431 or 432 or 433 or 434 or 435 or 436 or 437 or 438 or 439 or 440 or 441 or 442 or 443 or 444 or 445 or 446 or 447 or 448 or 449 or 450 or 451 or 452 or 453 or 454 or 455 or 456 or 457 or 458 or 459 or 460 or 461 or 462 or 463 or 464 or 465 or 466 or 467 or 468 or 469 or 470 or 471 or 472 or 485 or 486 or 487 or 488 or 489 or 490 or 491 or 492 or 493 or 494 or 495 or 496 or 497 or 498 or 499 or 500 or 501 or 502 or 503 or 504 or 505 or 506 or 507 or 508 or 509 or 510 or 511 or 512 or 513 or 514 or 515 or 516 or 517 or 518 or 519 or 520 or 521 or 522 or 523 or 524 or 525 or 526 or 527 or 528 or 529 or 530 or 531 or 532 or 533 or 534 or 535 or 536 or 537 or 538 or 539 or 540 or 541 or 542 or 543 or 544 or 545 or 546 or 547 or 548 or 549 or 550 or 551 or 552 or 553 or 554 or 555 or 556 or 557 or 558 or 559 or 560 or 561 or 562 or 563 or 564 or 565 or 566 or 567 or 568 or 569 or 570 or 571 or 572 or 573 or 574 or 575 or 576 or 577 or 578 or 579 or 580 or 581 or 582 or 583 or 584 or 585 or 586 or 587 or 588 or 589 or 590 or 591 or 592 or 593 or 594 or 595 or 596 or 597 or 598 or 599 or 600 or 601 or 602 or 603 or 604 or 605 or 606 or 607 or 608 or 609 or 610 or 611 or 612 or 613 or 614 or 615 or 616 or 617 or 618 or 619 or 620 or 621 or 622 or 623 or 624 or 625 or 626 or 627 or 628 or 629 or 630 or 631 or 632 or 633 or 634 or 635 or 636 or 637 or 638 or 639 or 640 or 641 or 642 or 643 or 644 or 645 or 646 or 647 or 648 or 649 or 650 or 651 or 652 or 653 or 654 or 655 or 656 or 657 or 658 or 659 or 660 or 661 or 662 or 663 or 664 or 665 or 666 or 667 or 668 or 729 or 730 or 731 or 732 or 733 or 734 or 735 or 736 or 737 or 738 or 739 or 740 or 741 or 742 or 743 or 744 or 745 or 746 or 747 or 748 or 749 or 750 or 751 or 752 or 753 or 754 or 755 or 756 or 757 or 758 or 759 or 760 or 761 or 762 or 763 or 764 or 765 or 766 or 767 or 768 or 769 or 770 or 771 or 772 or 773 or 774 or 775 or 776 or 777 or 778 or 779 or 780 or 781 or 782 or 783 or 784 or 785 or 786 or 787 or 788 or 789 or 790 or 791 or 792 or 793 or 794 or 795 or 796 or 797 or 798 or 799 or 800 or 801 or 802 or 803 or 804 or 805 or 806 or 807 or 808 or 809 or 810 or 811 or 812 or 813 or 814 or 815 or 816 or 817 or 818 or 819 or 820 or 821 or 822 or 823 or 824 or 825 or 826 or 827 or 828 or 829 or 830 or 831 or 832 or 833 or 834 or 835 or 836 or 837 or 838 or 839 or 840 or 841 or 842 or 843 or 844 or 845 or 846 or 847 or 848 or 849 or 850 or 851 or 852

918. 1 or 2 or 3 or 4 or 5 or 6 or 7 or 8 or 9 or 10 or 11 or 12 or 58 or 59 or 60 or 61 or 62 or 63 or 64 or 65 or 66 or 67 or 68 or 69 or 416 or 417 or 418 or 419 or 420 or 421 or 422 or 423 or 424 or 425 or 426 or 427 or 473 or 474 or 475 or 476 or 477 or 478 or 479 or 480 or 481 or 482 or 483 or 484 or 853 or 854 or 855 or 856 or 857 or 858

919. 302 or 303 or 304 or 305 or 306 or 307 or 308 or 309 or 310 or 311 or 312 or 313 or 717 or 718 or 719 or 720 or 721 or 722 or 723 or 724 or 725 or 726 or 727 or 728 or 904 or 905 or 906 or 907 or 908 or 909 or 910 or 911 or 912 or 913 or 914 or 915 or 916

920. 254 or 255 or 256 or 257 or 258 or 259 or 260 or 261 or 262 or 263 or 264 or 265 or 266 or 267 or 268 or 269 or 270 or 271 or 272 or 273 or 274 or 275 or 276 or 277 or 278 or 279 or 280 or 281 or 282 or 283 or 284 or 285 or 286 or 287 or 288 or 289 or 290 or 291 or 292 or 293 or 294 or 295 or 296 or 297 or 298 or 299 or 300 or 301 or 669 or 670 or 671 or 672 or 673 or 674 or 675 or 676 or 677 or 678 or 679 or 680 or 681 or 682 or 683 or 684 or 685 or 686 or 687 or 688 or 689 or 690 or 691 or 692 or 693 or 694 or 695 or 696 or 697 or 698 or 699 or 700 or 701 or 702 or 703 or 704 or 705 or 706 or 707 or 708 or 709 or 710 or 711 or 712 or 713 or 714 or 715 or 716 or 859 or 860 or 861 or 862 or 863 or 864 or 865 or 866 or 867 or 868 or 869 or 870 or 871 or 872 or 873 or 874 or 875 or 876 or 877 or 878 or 879 or 880 or 881 or 882 or 883 or 884 or 885 or 886 or 887 or 888 or 889 or 890 or 891 or 892 or 893 or 894 or 895 or 896 or 897 or 898 or 899 or 900 or 901 or 902 or 903

921. 917 and 918 and 919 and 920

Health Management Information Consortium (HMIC)

1. community pharmacy.mp. or exp Community pharmacy/

2. community pharmacy.mp. or exp Pharmacies/

3. community pharmacist.mp. or exp Pharmacists/

4. exp Pharmacies/ or private pharmacy.mp.

5. exp Pharmacists/ or exp Community Pharmacy Services/ or private pharmacist.mp. or exp Pharmacies/

6. exp Ambulatory Care/ or outpatient pharmacy.mp.

7. outpatient pharmacist.mp.

8. ambulatory pharmacy.mp.

9. ambulatory pharmacist.mp.

10. retail pharmacy.mp.

11. retail pharmacist.mp. or exp Pharmacists/

12. pharmacy store.mp.

13. pharmacy shop.mp.

14. exp Medication Errors/ or drug error.mp.

15. drug safe*.mp.

16. exp Harm Reduction/ or drug harm.mp.

17. drug risk.mp.

18. drug event.mp.

19. drug problem.mp.

20. drug incident.mp.

21. drug concern.mp.

22. drug quality.mp.

23. drug interaction.mp. or exp Drug Interactions/

24. drug discrepancy.mp.

25. exp Inappropriate Prescribing/ or inappropriate drug.mp.

26. drug intervention.mp.

27. drug manag*.mp.

28. drug mistake.mp.

29. drug violation.mp.

30. drug omission.mp.

31. drug omit.mp.

32. drug comission.mp.

33. drug commit.mp.

34. drug mishap.mp.

35. improper drug.mp.

36. wrong drug.mp.

37. incorrect drug.mp.

38. nearmiss.mp.

39. drug misadventure.mp.

40. drug oversight.mp.

41. medication safe*.mp.

42. medication safety.mp.

43. medication harm.mp.

44. medication risk.mp.

45. medication event.mp.

46. medication problem.mp.

47. medication incident.mp.

48. medication concern.mp.

49. medication quality.mp.

50. medication interaction.mp.

51. medication discrepancy.mp.

52. inappropriate medication.mp.

53. medication intervention.mp.

54. medication management.mp.

55. medication mistake.mp.

56. medication violation.mp.

57. medication omission.mp.

58. medication commission.mp.

59. community pharmacy.mp. or exp Pharmacies/

60. community pharmacist.mp. or exp Pharmacists/

61. exp Pharmacies/ or private pharmacy.mp.

62. exp Pharmacists/ or exp Community Pharmacy Services/ or private pharmacist.mp. or exp Pharmacies/

63. exp Ambulatory Care/ or outpatient pharmacy.mp.

64. outpatient pharmacist.mp.

65. ambulatory pharmacy.mp.

66. ambulatory pharmacist.mp.

67. retail pharmacy.mp.

68. retail pharmacist.mp. or exp Pharmacists/

69. pharmacy store.mp.

70. pharmacy shop.mp.

71. exp Medication Errors/ or drug error.mp.

72. drug safe*.mp.

73. exp Harm Reduction/ or drug harm.mp.

74. drug risk.mp.

75. drug event.mp.

76. drug problem.mp.

77. drug incident.mp.

78. drug concern.mp.

79. drug interaction.mp. or exp Drug Interactions/

80. drug discrepancy.mp.

81. drug intervention.mp.

82. drug manag*.mp.

83. drug mistake.mp.

84. drug violation.mp.

85. drug omission.mp.

86. drug commit.mp.

87. drug mishap.mp.

88. improper drug.mp.

89. wrong drug.mp.

90. incorrect drug.mp.

91. nearmiss.mp.

92. drug misadventure.mp.

93. drug oversight.mp.

94. medication safe*.mp.

95. medication safety.mp.

96. medication harm.mp.

97. medication risk.mp.

98. medication event.mp.

99. medication problem.mp.

100. medication incident.mp.

101. medication concern.mp.

102. medication quality.mp.

103. medication interaction.mp.

104. medication discrepancy.mp.

105. inappropriate medication.mp.

106. medication intervention.mp.

107. medication management.mp.

108. medication mistake.mp.

109. medication violation.mp.

110. medication omission.mp.

111. medication commission.mp.

112. medication mishap.mp.

113. improper medication.mp.

114. wrong medication.mp.

115. incorrect medication.mp.

116. nearmiss.mp.

117. medication misadventure.mp.

118. medication oversight.mp.

119. safe therapy.mp.

120. therapy error.mp.

121. therapy risk.mp.

122. therapy event.mp.

123. therapy harm.mp.

124. therapy problem.mp.

125. therapy incident.mp.

126. therapy concern.mp.

127. therapy quality.mp.

128. therapy interaction.mp.

129. therapy discrepancy.mp.

130. Inappropriate therapy.mp.

131. therapy management.mp.

132. therapy mistake.mp.

133. therapy violation.mp.

134. therapy omission.mp.

135. therapy omit.mp.

136. therapy commission.mp.

137. therapy commit.mp.

138. therapy mishap.mp.

139. medication duplication.mp.

140. therapy duplication.mp.

141. drug duplication.mp.

142. therapy mishap.mp.

143. improper therapy.mp.

144. wrong therapy.mp.

145. incorrect therapy.mp.

146. therapy misadventure.mp.

147. therapy oversight.mp.

148. prescribing error.mp.

149. prescription error.mp.

150. prescribing harm.mp.

151. prescription harm.mp.

152. prescription risk.mp.

153. prescribing risk.mp.

154. prescribing problem.mp.

155. prescription problem.mp.

156. prescribing incident.mp.

157. prescription incident.mp.

158. prescription concern.mp.

159. prescribing concern.mp.

160. prescription quality.mp.

161. prescribing quality.mp.

162. prescription discrepancy.mp.

163. prescribing discrepancy.mp.

164. inappropriate prescription.mp. or exp Inappropriate Prescribing/

165. improper prescribing.mp.

166. improper prescription.mp.

167. prescribing intervention.mp.

168. prescription intervention.mp.

169. prescribing mistake.mp.

170. prescription mistake.mp.

171. prescription violation.mp.

172. prescribing violation.mp.

173. prescription mishap.mp.

174. prescribing mishap.mp.

175. wrong prescription.mp.

176. wrong prescribing.mp.

177. incorrect prescription.mp.

178. incorrect prescribing.mp.

179. prescription misadventure.mp.

180. prescribing misadventure.mp.

181. dispensing error.mp.

182. dispensing harm.mp.

183. dispensing risk.mp.

184. dispensing event.mp.

185. dispensing problem.mp.

186. dispensing incident.mp.

187. dispensing concern.mp.

188. dispensing quality.mp.

189. dispensing discrepancy.mp.

190. inappropriate dispensing.mp.

191. dispensing mistake.mp.

192. dispensing violation.mp.

193. dispensing omission.mp.

194. dispensing commission.mp.

195. dispensing duplication.mp.

196. dispensing mishap.mp.

197. improper dispensing.mp.

198. wrong dispensing.mp.

199. incorrect dispensing.mp.

200. dispensing misadventure.mp.

201. dispensing oversight.mp.

202. transcribing error.mp.

203. transcribing harm.mp.

204. transcribing risk.mp.

205. transcribing event.mp.

206. transcribing problem.mp.

207. transcribing incident.mp.

208. transcribing concern.mp.

209. transcribing quality.mp.

210. transcribing discrepancy.mp.

211. inappropriate transcribing.mp.

212. improper transcribing.mp.

213. transcribing mistake.mp.

214. transcribing duplication.mp.

215. wrong transcribing.mp.

216. incorrect transcribing.mp.

217. transcribing misadventure.mp.

218. administration error.mp.

219. administration harm.mp.

220. administration event.mp.

221. administration risk.mp.

222. administration problem.mp.

223. administration incident.mp.

224. administration concern.mp.

225. administration discrepancy.mp.

226. inappropriate administration.mp.

227. administration mistake.mp.

228. administration violation.mp.

229. administration omission.mp.

230. administration commission.mp.

231. administration duplication.mp.

232. administration mishap.mp.

233. improper administration.mp.

234. wrong administration.mp.

235. incorrect administration.mp.

236. administration misadventure.mp.

237. administration oversight.mp.

238. inappropriate route of administration.mp.

239. wrong route of administration.mp.

240. incorrect route of administration.mp.

241. improper route of administration.mp.

242. wrong dose.mp.

243. incorrect dose.mp.

244. inappropriate dose.mp.

245. improper dose.mp.

246. dose duplication.mp.

247. dose commission.mp.

248. dose omission.mp.

249. overprescribing.mp.

250. error in calculation.mp.

251. inappropriate calculation.mp.

252. improper calculation.mp.

253. wrong calculation.mp.

254. calculation problem.mp.

255. exp "Root Cause Analysis"/ or Cause.mp.

256. factor.mp. or exp Time Factors/

257. human.mp. or exp Humans/

258. work/ or "personnel staffing and scheduling"/

259. work.mp.

260. exp Financial Stress/

261. stress.mp. or exp Occupational Stress/ or exp Stress, Psychological/

262. exp Burnout, Psychological/ or exp Burnout, Professional/ or burnout.mp.

263. system factor.mp.

264. system.mp.

265. element.mp. or exp Elements/

266. organization*.mp.

267. origin.mp.

268. root.mp.

269. provocation.mp.

270. source.mp.

271. incentive.mp. or exp Motivation/

272. occasion.mp.

273. explanation.mp.

274. catalyst.mp.

275. stimulus.mp. or exp Generalization, Stimulus/

276. trigger.mp.

277. risk factor.mp. or exp Risk Factors/

278. origin.mp.

279. Ergonomics.mp. or exp Ergonomics/

280. systems analysis/ or systems integration/ or workflow/

281. motive.mp.

282. goal.mp. or exp Goals/

283. impetus.mp.

284. rationale.mp.

285. basis.mp.

286. ground.mp.

287. etiology.mp.

288. reason.mp.

289. engineer*.mp.

290. interface.mp.

291. component.mp.

292. aspect.mp.

293. variable.mp.

294. influence.mp.

295. determinant.mp.

296. contributor.mp.

297. parameter.mp.

298. feature.mp.

299. challenge.mp.

300. barrier.mp.

301. obstacle.mp.

302. facilitator.mp.

303. qualitative design.mp. or exp Interviews as Topic/ or Qualitative Research/

304. mixed methods.mp.

305. focus group.mp. or exp Focus Groups/

306. ethnograph*.mp.

307. exp Anthropology, Cultural/ or exp Anthropology/ or anthropology*.mp.

308. exp Interview, Psychological/ or exp Interview/ or interview.mp.

309. theme.mp.

310. thematic.mp.

311. code.mp.

312. quote.mp.

313. field note.mp.

314. observation.mp. or exp Observation/

315. exp drug safety/

316. drug quality.mp. or exp drug quality/

317. drug omit*.mp.

318. drug commission.mp.

319. drug commit*.mp.

320. drug duplicat*.mp.

321. exp inappropriate prescribing/ or inappropriate drug.mp. or exp potentially inappropriate medication/

322. medication manag*.mp.

323. medication omit*.mp.

324. medication commit*.mp.

325. medication duplicat*.mp.

326. exp prescribing error/

327. exp prescribing error/

328. exp dispensing error/

329. Administer* error.mp.

330. Administering error.mp.

331. transcrib* error.mp.

332. monitor* error.mp.

333. therapy error.mp. or exp therapeutic error/

334. therapy omit*.mp.

335. therapy commit*.mp.

336. therapy commission.mp.

337. therapy duplicat*.mp.

338. prescrib* harm.mp.

339. prescrib* risk.mp.

340. prescrib* event.mp.

341. prescrib* incident.mp.

342. prescrib* problem.mp.

343. prescrib* quality.mp.

344. prescrib* interaction.mp.

345. prescrib* discrepancy.mp.

346. prescrib* inappropriate.mp.

347. prescrib* intervention.mp.

348. prescrib* omission.mp.

349. prescrib* duplicat*.mp.

350. improper prescrib*.mp.

351. wrong prescribing.mp.

352. wrong prescription.mp.

353. incorrect prescribing.mp.

354. incorrect prescription.mp.

355. prescribing misadventure.mp.

356. prescription misadventure.mp.

357. prescrib* oversight.mp.

358. prescription oversight.mp.

359. dispensing management.mp.

360. dispensing violation.mp.

361. dispensing omit*.mp.

362. dispensing commit.mp.

363. dispensing commission.mp.

364. dispensing duplication.mp.

365. dispensing mishap.mp.

366. Administration omit.mp.

367. administration commit.mp.

368. administration commission.mp.

369. administer* error.mp.

370. administer* event.mp.

371. administer* risk.mp.

372. administer* harm.mp.

373. administer* incident.mp.

374. improper administer*.mp.

375. incorrect administer*.mp.

376. administer* discrepancy.mp.

377. administer* omission.mp.

378. administer* commission.mp.

379. administer* omit.mp.

380. administer* commit.mp.

381. administer* problem.mp.

382. administer* duplication.mp.

383. administer* violation.mp.

384. administer* mishap.mp.

385. administer* oversight.mp.

386. administer* incident.mp.

387. administer* concern.mp.

388. administer* quality.mp.

389. administer* management.mp.

390. administer* intervention.mp.

391. monitor* Safe*.mp.

392. monitor* error.mp.

393. monitor* harm.mp.

394. monitor* risk.mp.

395. Monitor* event.mp.

396. Monitor* problem.mp.

397. Monitor* incident.mp.

398. Monitor* concern.mp.

399. monitor* quality.mp.

400. monitor* interaction.mp.

401. Monitor* discrepancy.mp.

402. inappropriate Monitor*.mp.

403. Monitor* intervention.mp.

404. monitor* mistake.mp.

405. monitor* violation.mp.

406. Monitor* omission.mp.

407. monitor* omit.mp.

408. monitor* commission.mp.

409. monitor* commit.mp.

410. monitor* mishap.mp.

411. improper monitor*.mp.

412. wrong monitor*.mp.

413. wrong monitor*.mp.

414. incorrect monitor*.mp.

415. monitor* misadventure.mp.

416. monitor* oversight.mp.

417. community pharmacy.mp. or exp Pharmacies/

418. community pharmacist.mp. or exp Pharmacists/

419. exp Pharmacies/ or private pharmacy.mp.

420. exp Pharmacists/ or exp Community Pharmacy Services/ or private pharmacist.mp. or exp Pharmacies/

421. exp Ambulatory Care/ or outpatient pharmacy.mp.

422. outpatient pharmacist.mp.

423. ambulatory pharmacy.mp.

424. ambulatory pharmacist.mp.

425. retail pharmacy.mp.

426. retail pharmacist.mp. or exp Pharmacists/

427. pharmacy store.mp.

428. pharmacy shop.mp.

429. exp Medication Errors/ or drug error.mp.

430. drug safe*.mp.

431. exp Harm Reduction/ or drug harm.mp.

432. drug risk.mp.

433. drug event.mp.

434. drug problem.mp.

435. drug incident.mp.

436. drug concern.mp.

437. drug quality.mp.

438. drug interaction.mp. or exp Drug Interactions/

439. drug discrepancy.mp.

440. exp Inappropriate Prescribing/ or inappropriate drug.mp.

441. drug intervention.mp.

442. drug manag*.mp.

443. drug mistake.mp.

444. drug violation.mp.

445. drug omission.mp.

446. drug omit.mp.

447. drug comission.mp.

448. drug commit.mp.

449. drug mishap.mp.

450. improper drug.mp.

451. wrong drug.mp.

452. incorrect drug.mp.

453. nearmiss.mp.

454. drug misadventure.mp.

455. drug oversight.mp.

456. medication safe*.mp.

457. medication safety.mp.

458. medication harm.mp.

459. medication risk.mp.

460. medication event.mp.

461. medication problem.mp.

462. medication incident.mp.

463. medication concern.mp.

464. medication quality.mp.

465. medication interaction.mp.

466. medication discrepancy.mp.

467. inappropriate medication.mp.

468. medication intervention.mp.

469. medication management.mp.

470. medication mistake.mp.

471. medication violation.mp.

472. medication omission.mp.

473. medication commission.mp.

474. community pharmacy.mp. or exp Pharmacies/

475. community pharmacist.mp. or exp Pharmacists/

476. exp Pharmacies/ or private pharmacy.mp.

477. exp Pharmacists/ or exp Community Pharmacy Services/ or private pharmacist.mp. or exp Pharmacies/

478. exp Ambulatory Care/ or outpatient pharmacy.mp.

479. outpatient pharmacist.mp.

480. ambulatory pharmacy.mp.

481. ambulatory pharmacist.mp.

482. retail pharmacy.mp.

483. retail pharmacist.mp. or exp Pharmacists/

484. pharmacy store.mp.

485. pharmacy shop.mp.

486. exp Medication Errors/ or drug error.mp.

487. drug safe*.mp.

488. exp Harm Reduction/ or drug harm.mp.

489. drug risk.mp.

490. drug event.mp.

491. drug problem.mp.

492. drug incident.mp.

493. drug concern.mp.

494. drug interaction.mp. or exp Drug Interactions/

495. drug discrepancy.mp.

496. drug intervention.mp.

497. drug manag*.mp.

498. drug mistake.mp.

499. drug violation.mp.

500. drug omission.mp.

501. drug commit.mp.

502. drug mishap.mp.

503. improper drug.mp.

504. wrong drug.mp.

505. incorrect drug.mp.

506. nearmiss.mp.

507. drug misadventure.mp.

508. drug oversight.mp.

509. medication safe*.mp.

510. medication safety.mp.

511. medication harm.mp.

512. medication risk.mp.

513. medication event.mp.

514. medication problem.mp.

515. medication incident.mp.

516. medication concern.mp.

517. medication quality.mp.

518. medication interaction.mp.

519. medication discrepancy.mp.

520. inappropriate medication.mp.

521. medication intervention.mp.

522. medication management.mp.

523. medication mistake.mp.

524. medication violation.mp.

525. medication omission.mp.

526. medication commission.mp.

527. medication mishap.mp.

528. improper medication.mp.

529. wrong medication.mp.

530. incorrect medication.mp.

531. nearmiss.mp.

532. medication misadventure.mp.

533. medication oversight.mp.

534. safe therapy.mp.

535. therapy error.mp.

536. therapy risk.mp.

537. therapy event.mp.

538. therapy harm.mp.

539. therapy problem.mp.

540. therapy incident.mp.

541. therapy concern.mp.

542. therapy quality.mp.

543. therapy interaction.mp.

544. therapy discrepancy.mp.

545. Inappropriate therapy.mp.

546. therapy management.mp.

547. therapy mistake.mp.

548. therapy violation.mp.

549. therapy omission.mp.

550. therapy omit.mp.

551. therapy commission.mp.

552. therapy commit.mp.

553. therapy mishap.mp.

554. medication duplication.mp.

555. therapy duplication.mp.

556. drug duplication.mp.

557. therapy mishap.mp.

558. improper therapy.mp.

559. wrong therapy.mp.

560. incorrect therapy.mp.

561. therapy misadventure.mp.

562. therapy oversight.mp.

563. prescribing error.mp.

564. prescription error.mp.

565. prescribing harm.mp.

566. prescription harm.mp.

567. prescription risk.mp.

568. prescribing risk.mp.

569. prescribing problem.mp.

570. prescription problem.mp.

571. prescribing incident.mp.

572. prescription incident.mp.

573. prescription concern.mp.

574. prescribing concern.mp.

575. prescription quality.mp.

576. prescribing quality.mp.

577. prescription discrepancy.mp.

578. prescribing discrepancy.mp.

579. inappropriate prescription.mp. or exp Inappropriate Prescribing/

580. improper prescribing.mp.

581. improper prescription.mp.

582. prescribing intervention.mp.

583. prescription intervention.mp.

584. prescribing mistake.mp.

585. prescription mistake.mp.

586. prescription violation.mp.

587. prescribing violation.mp.

588. prescription mishap.mp.

589. prescribing mishap.mp.

590. wrong prescription.mp.

591. wrong prescribing.mp.

592. incorrect prescription.mp.

593. incorrect prescribing.mp.

594. prescription misadventure.mp.

595. prescribing misadventure.mp.

596. dispensing error.mp.

597. dispensing harm.mp.

598. dispensing risk.mp.

599. dispensing event.mp.

600. dispensing problem.mp.

601. dispensing incident.mp.

602. dispensing concern.mp.

603. dispensing quality.mp.

604. dispensing discrepancy.mp.

605. inappropriate dispensing.mp.

606. dispensing mistake.mp.

607. dispensing violation.mp.

608. dispensing omission.mp.

609. dispensing commission.mp.

610. dispensing duplication.mp.

611. dispensing mishap.mp.

612. improper dispensing.mp.

613. wrong dispensing.mp.

614. incorrect dispensing.mp.

615. dispensing misadventure.mp.

616. dispensing oversight.mp.

617. transcribing error.mp.

618. transcribing harm.mp.

619. transcribing risk.mp.

620. transcribing event.mp.

621. transcribing problem.mp.

622. transcribing incident.mp.

623. transcribing concern.mp.

624. transcribing quality.mp.

625. transcribing discrepancy.mp.

626. inappropriate transcribing.mp.

627. improper transcribing.mp.

628. transcribing mistake.mp.

629. transcribing duplication.mp.

630. wrong transcribing.mp.

631. incorrect transcribing.mp.

632. transcribing misadventure.mp.

633. administration error.mp.

634. administration harm.mp.

635. administration event.mp.

636. administration risk.mp.

637. administration problem.mp.

638. administration incident.mp.

639. administration concern.mp.

640. administration discrepancy.mp.

641. inappropriate administration.mp.

642. administration mistake.mp.

643. administration violation.mp.

644. administration omission.mp.

645. administration commission.mp.

646. administration duplication.mp.

647. administration mishap.mp.

648. improper administration.mp.

649. wrong administration.mp.

650. incorrect administration.mp.

651. administration misadventure.mp.

652. administration oversight.mp.

653. inappropriate route of administration.mp.

654. wrong route of administration.mp.

655. incorrect route of administration.mp.

656. improper route of administration.mp.

657. wrong dose.mp.

658. incorrect dose.mp.

659. inappropriate dose.mp.

660. improper dose.mp.

661. dose duplication.mp.

662. dose commission.mp.

663. dose omission.mp.

664. overprescribing.mp.

665. error in calculation.mp.

666. inappropriate calculation.mp.

667. improper calculation.mp.

668. wrong calculation.mp.

669. calculation problem.mp.

670. exp "Root Cause Analysis"/ or Cause.mp.

671. factor.mp. or exp Time Factors/

672. human.mp. or exp Humans/

673. work/ or "personnel staffing and scheduling"/

674. work.mp.

675. exp Financial Stress/

676. stress.mp. or exp Occupational Stress/ or exp Stress, Psychological/

677. exp Burnout, Psychological/ or exp Burnout, Professional/ or burnout.mp.

678. system factor.mp.

679. system.mp.

680. element.mp. or exp Elements/

681. organization*.mp.

682. origin.mp.

683. root.mp.

684. provocation.mp.

685. source.mp.

686. incentive.mp. or exp Motivation/

687. occasion.mp.

688. explanation.mp.

689. catalyst.mp.

690. stimulus.mp. or exp Generalization, Stimulus/

691. trigger.mp.

692. risk factor.mp. or exp Risk Factors/

693. origin.mp.

694. Ergonomics.mp. or exp Ergonomics/

695. systems analysis/ or systems integration/ or workflow/

696. motive.mp.

697. goal.mp. or exp Goals/

698. impetus.mp.

699. rationale.mp.

700. basis.mp.

701. ground.mp.

702. etiology.mp.

703. reason.mp.

704. engineer*.mp.

705. interface.mp.

706. component.mp.

707. aspect.mp.

708. variable.mp.

709. influence.mp.

710. determinant.mp.

711. contributor.mp.

712. parameter.mp.

713. feature.mp.

714. challenge.mp.

715. barrier.mp.

716. obstacle.mp.

717. facilitator.mp.

718. qualitative design.mp. or exp Interviews as Topic/ or Qualitative Research/

719. mixed methods.mp.

720. focus group.mp. or exp Focus Groups/

721. ethnograph*.mp.

722. exp Anthropology, Cultural/ or exp Anthropology/ or anthropology*.mp.

723. exp Interview, Psychological/ or exp Interview/ or interview.mp.

724. theme.mp.

725. thematic.mp.

726. code.mp.

727. quote.mp.

728. field note.mp.

729. observation.mp. or exp Observation/

730. exp drug safety/

731. drug quality.mp. or exp drug quality/

732. drug omit*.mp.

733. drug commission.mp.

734. drug commit*.mp.

735. drug duplicat*.mp.

736. exp inappropriate prescribing/ or inappropriate drug.mp. or exp potentially inappropriate medication/

737. medication manag*.mp.

738. medication omit*.mp.

739. medication commit*.mp.

740. medication duplicat*.mp.

741. exp prescribing error/

742. exp prescribing error/

743. exp dispensing error/

744. Administer* error.mp.

745. Administering error.mp.

746. transcrib* error.mp.

747. monitor* error.mp.

748. therapy error.mp. or exp therapeutic error/

749. therapy omit*.mp.

750. therapy commit*.mp.

751. therapy commission.mp.

752. therapy duplicat*.mp.

753. prescrib* harm.mp.

754. prescrib* risk.mp.

755. prescrib* event.mp.

756. prescrib* incident.mp.

757. prescrib* problem.mp.

758. prescrib* quality.mp.

759. prescrib* interaction.mp.

760. prescrib* discrepancy.mp.

761. prescrib* inappropriate.mp.

762. prescrib* intervention.mp.

763. prescrib* omission.mp.

764. prescrib* duplicat*.mp.

765. improper prescrib*.mp.

766. wrong prescribing.mp.

767. wrong prescription.mp.

768. incorrect prescribing.mp.

769. incorrect prescription.mp.

770. prescribing misadventure.mp.

771. prescription misadventure.mp.

772. prescrib* oversight.mp.

773. prescription oversight.mp.

774. dispensing management.mp.

775. dispensing violation.mp.

776. dispensing omit*.mp.

777. dispensing commit.mp.

778. dispensing commission.mp.

779. dispensing duplication.mp.

780. dispensing mishap.mp.

781. Administration omit.mp.

782. administration commit.mp.

783. administration commission.mp.

784. administer* error.mp.

785. administer* event.mp.

786. administer* risk.mp.

787. administer* harm.mp.

788. administer* incident.mp.

789. improper administer*.mp.

790. incorrect administer*.mp.

791. administer* discrepancy.mp.

792. administer* omission.mp.

793. administer* commission.mp.

794. administer* omit.mp.

795. administer* commit.mp.

796. administer* problem.mp.

797. administer* duplication.mp.

798. administer* violation.mp.

799. administer* mishap.mp.

800. administer* oversight.mp.

801. administer* incident.mp.

802. administer* concern.mp.

803. administer* quality.mp.

804. administer* management.mp.

805. administer* intervention.mp.

806. monitor* Safe*.mp.

807. monitor* error.mp.

808. monitor* harm.mp.

809. monitor* risk.mp.

810. Monitor* event.mp.

811. Monitor* problem.mp.

812. Monitor* incident.mp.

813. Monitor* concern.mp.

814. monitor* quality.mp.

815. monitor* interaction.mp.

816. Monitor* discrepancy.mp.

817. inappropriate Monitor*.mp.

818. Monitor* intervention.mp.

819. monitor* mistake.mp.

820. monitor* violation.mp.

821. Monitor* omission.mp.

822. monitor* omit.mp.

823. monitor* commission.mp.

824. monitor* commit.mp.

825. monitor* mishap.mp.

826. improper monitor*.mp.

827. wrong monitor*.mp.

828. wrong monitor*.mp.

829. incorrect monitor*.mp.

830. monitor* misadventure.mp.

831. monitor* oversight.mp.

832. prescribing commit.mp.

833. prescription commit.mp.

834. prescription commission.mp.

835. prescription commit.mp.

836. label* error.mp.

837. label* problem.mp.

838. label* concern.mp.

839. label* quality.mp.

840. label* discrepancy.mp.

841. inappropriate label*.mp.

842. label* mistake.mp.

843. label* violation.mp.

844. label* omission.mp.

845. label* omit.mp.

846. label* commission.mp.

847. label* commit.mp.

848. label* mishap.mp.

849. improper label*.mp.

850. wrong label*.mp.

851. incorrect label*.mp.

852. label* misadventure.mp.

853. label* oversight.mp.

854. exp "pharmacy (shop)"/

855. outpatient pharmacy.mp. or exp pharmacy/

856. exp ambulatory care/ or exp pharmacist/ or ambulatory pharmacy.mp. or exp pharmacy/

857. retail pharmacy.mp.

858. pharmacy store.mp.

859. community pharmacist.mp. or exp community pharmacist/

860. factor.mp. or exp time factor/ or exp cultural factor/ or exp risk factor/

861. human.mp. or exp human factors research/

862. exp work experience/

863. stress.mp. or exp physiological stress/

864. exp professional burnout/ or exp burnout/ or burnout.mp.

865. exp organizational citizenship/ or exp organizational climate/ or exp organizational culture/ or exp safety climate/ or exp safety culture/ or exp organizational decision making/ or exp organizational development/ or exp organizational efficiency/ or exp organizational policy/

866. exp health care system/ or exp "root system"/ or system.mp. or exp medication system/ or exp metric system/

867. element.mp.

868. origin.mp.

869. exp "root cause analysis"/ or root.mp.

870. provocation.mp. or exp provocation/

871. source.mp.

872. incentive.mp. or exp incentive/ or exp social incentive/

873. occasion.mp.

874. explanation.mp.

875. explain*.mp.

876. catalyst.mp.

877. stimulus.mp.

878. trigger.mp.

879. risk factor.mp. or exp risk factor/

880. origin.mp.

881. Ergonomics.mp. or exp ergonomics/

882. exp systems theory/

883. exp motivation/ or motiv*.mp.

884. goal.mp.

885. impetus.mp.

886. rationale.mp.

887. basis.mp.

888. ground.mp.

889. etiology.mp. or exp etiology/

890. reason.mp.

891. engineer*.mp.

892. interface.mp.

893. component.mp.

894. exp psychological aspect/ or aspect.mp. or exp economic aspect/ or exp social aspect/

895. exp explanatory variable/ or exp predictor variable/ or exp independent variable/ or variable.mp.

896. influenc*.mp.

897. determinant.mp.

898. contribut*.mp.

899. parameter.mp. or exp parameters/

900. feature.mp.

901. challenge.mp.

902. barrier.mp.

903. obstacle.mp.

904. facilitator.mp.

905. exp qualitative research/ or exp qualitative analysis/ or Qualitative.mp.

906. exp interview/ or mixed method.mp.

907. focus group.mp.

908. exp ethnographic research/ or exp ethnography/ or ethnograph*.mp.

909. exp cultural anthropology/ or exp anthropology/ or anthropology.mp.

910. exp structured interview/ or exp telephone interview/ or exp audio interview/ or exp unstructured interview/ or interview.mp. or exp psychological interview/ or exp video interview/ or exp semi structured interview/ or exp interview/

911. theme.mp.

912. exp thematic analysis/ or thematic.mp.

913. code.mp.

914. quote.mp.

915. field note.mp.

916. exp field work/

917. exp participant observation/ or exp non-participant observation/ or exp observation/ or observation.mp.

918. 14 or 15 or 16 or 17 or 18 or 19 or 20 or 21 or 22 or 23 or 24 or 25 or 26 or 27 or 28 or 29 or 30 or 31 or 32 or 33 or 34 or 35 or 36 or 37 or 38 or 39 or 40 or 41 or 42 or 43 or 44 or 45 or 46 or 47 or 48 or 49 or 50 or 51 or 52 or 53 or 54 or 55 or 56 or 57 or 58 or 71 or 72 or 73 or 74 or 75 or 76 or 77 or 78 or 79 or 80 or 81 or 82 or 83 or 84 or 85 or 86 or 87 or 88 or 89 or 90 or 91 or 92 or 93 or 94 or 95 or 96 or 97 or 98 or 99 or 100 or 101 or 102 or 103 or 104 or 105 or 106 or 107 or 108 or 109 or 110 or 111 or 112 or 113 or 114 or 115 or 116 or 117 or 118 or 119 or 120 or 121 or 122 or 123 or 124 or 125 or 126 or 127 or 128 or 129 or 130 or 131 or 132 or 133 or 134 or 135 or 136 or 137 or 138 or 139 or 140 or 141 or 142 or 143 or 144 or 145 or 146 or 147 or 148 or 149 or 150 or 151 or 152 or 153 or 154 or 155 or 156 or 157 or 158 or 159 or 160 or 161 or 162 or 163 or 164 or 165 or 166 or 167 or 168 or 169 or 170 or 171 or 172 or 173 or 174 or 175 or 176 or 177 or 178 or 179 or 180 or 181 or 182 or 183 or 184 or 185 or 186 or 187 or 188 or 189 or 190 or 191 or 192 or 193 or 194 or 195 or 196 or 197 or 198 or 199 or 200 or 201 or 202 or 203 or 204 or 205 or 206 or 207 or 208 or 209 or 210 or 211 or 212 or 213 or 214 or 215 or 216 or 217 or 218 or 219 or 220 or 221 or 222 or 223 or 224 or 225 or 226 or 227 or 228 or 229 or 230 or 231 or 232 or 233 or 234 or 235 or 236 or 237 or 238 or 239 or 240 or 241 or 242 or 243 or 244 or 245 or 246 or 247 or 248 or 249 or 250 or 251 or 252 or 253 or 254 or 315 or 316 or 317 or 318 or 319 or 320 or 321 or 322 or 323 or 324 or 325 or 326 or 327 or 328 or 329 or 330 or 331 or 332 or 333 or 334 or 335 or 336 or 337 or 338 or 339 or 340 or 341 or 342 or 343 or 344 or 345 or 346 or 347 or 348 or 349 or 350 or 351 or 352 or 353 or 354 or 355 or 356 or 357 or 358 or 359 or 360 or 361 or 362 or 363 or 364 or 365 or 366 or 367 or 368 or 369 or 370 or 371 or 372 or 373 or 374 or 375 or 376 or 377 or 378 or 379 or 380 or 381 or 382 or 383 or 384 or 385 or 386 or 387 or 388 or 389 or 390 or 391 or 392 or 393 or 394 or 395 or 396 or 397 or 398 or 399 or 400 or 401 or 402 or 403 or 404 or 405 or 406 or 407 or 408 or 409 or 410 or 411 or 412 or 413 or 414 or 415 or 416 or 429 or 430 or 431 or 432 or 433 or 434 or 435 or 436 or 437 or 438 or 439 or 440 or 441 or 442 or 443 or 444 or 445 or 446 or 447 or 448 or 449 or 450 or 451 or 452 or 453 or 454 or 455 or 456 or 457 or 458 or 459 or 460 or 461 or 462 or 463 or 464 or 465 or 466 or 467 or 468 or 469 or 470 or 471 or 472 or 473 or 486 or 487 or 488 or 489 or 490 or 491 or 492 or 493 or 494 or 495 or 496 or 497 or 498 or 499 or 500 or 501 or 502 or 503 or 504 or 505 or 506 or 507 or 508 or 509 or 510 or 511 or 512 or 513 or 514 or 515 or 516 or 517 or 518 or 519 or 520 or 521 or 522 or 523 or 524 or 525 or 526 or 527 or 528 or 529 or 530 or 531 or 532 or 533 or 534 or 535 or 536 or 537 or 538 or 539 or 540 or 541 or 542 or 543 or 544 or 545 or 546 or 547 or 548 or 549 or 550 or 551 or 552 or 553 or 554 or 555 or 556 or 557 or 558 or 559 or 560 or 561 or 562 or 563 or 564 or 565 or 566 or 567 or 568 or 569 or 570 or 571 or 572 or 573 or 574 or 575 or 576 or 577 or 578 or 579 or 580 or 581 or 582 or 583 or 584 or 585 or 586 or 587 or 588 or 589 or 590 or 591 or 592 or 593 or 594 or 595 or 596 or 597 or 598 or 599 or 600 or 601 or 602 or 603 or 604 or 605 or 606 or 607 or 608 or 609 or 610 or 611 or 612 or 613 or 614 or 615 or 616 or 617 or 618 or 619 or 620 or 621 or 622 or 623 or 624 or 625 or 626 or 627 or 628 or 629 or 630 or 631 or 632 or 633 or 634 or 635 or 636 or 637 or 638 or 639 or 640 or 641 or 642 or 643 or 644 or 645 or 646 or 647 or 648 or 649 or 650 or 651 or 652 or 653 or 654 or 655 or 656 or 657 or 658 or 659 or 660 or 661 or 662 or 663 or 664 or 665 or 666 or 667 or 668 or 669 or 730 or 731 or 732 or 733 or 734 or 735 or 736 or 737 or 738 or 739 or 740 or 741 or 742 or 743 or 744 or 745 or 746 or 747 or 748 or 749 or 750 or 751 or 752 or 753 or 754 or 755 or 756 or 757 or 758 or 759 or 760 or 761 or 762 or 763 or 764 or 765 or 766 or 767 or 768 or 769 or 770 or 771 or 772 or 773 or 774 or 775 or 776 or 777 or 778 or 779 or 780 or 781 or 782 or 783 or 784 or 785 or 786 or 787 or 788 or 789 or 790 or 791 or 792 or 793 or 794 or 795 or 796 or 797 or 798 or 799 or 800 or 801 or 802 or 803 or 804 or 805 or 806 or 807 or 808 or 809 or 810 or 811 or 812 or 813 or 814 or 815 or 816 or 817 or 818 or 819 or 820 or 821 or 822 or 823 or 824 or 825 or 826 or 827 or 828 or 829 or 830 or 831 or 832 or 833 or 834 or 835 or 836 or 837 or 838 or 839 or 840 or 841 or 842 or 843 or 844 or 845 or 846 or 847 or 848 or 849 or 850 or 851 or 852 or 853

919. 2 or 3 or 4 or 5 or 6 or 7 or 8 or 9 or 10 or 11 or 12 or 13 or 59 or 60 or 61 or 62 or 63 or 64 or 65 or 66 or 67 or 68 or 69 or 70 or 417 or 418 or 419 or 420 or 421 or 422 or 423 or 424 or 425 or 426 or 427 or 428 or 474 or 475 or 476 or 477 or 478 or 479 or 480 or 481 or 482 or 483 or 484 or 485 or 854 or 855 or 856 or 857 or 858 or 859

920. 303 or 304 or 305 or 306 or 307 or 308 or 309 or 310 or 311 or 312 or 313 or 314 or 718 or 719 or 720 or 721 or 722 or 723 or 724 or 725 or 726 or 727 or 728 or 729 or 905 or 906 or 907 or 908 or 909 or 910 or 911 or 912 or 913 or 914 or 915 or 916 or 917

921. 255 or 256 or 257 or 258 or 259 or 260 or 261 or 262 or 263 or 264 or 265 or 266 or 267 or 268 or 269 or 270 or 271 or 272 or 273 or 274 or 275 or 276 or 277 or 278 or 279 or 280 or 281 or 282 or 283 or 284 or 285 or 286 or 287 or 288 or 289 or 290 or 291 or 292 or 293 or 294 or 295 or 296 or 297 or 298 or 299 or 300 or 301 or 302 or 670 or 671 or 672 or 673 or 674 or 675 or 676 or 677 or 678 or 679 or 680 or 681 or 682 or 683 or 684 or 685 or 686 or 687 or 688 or 689 or 690 or 691 or 692 or 693 or 694 or 695 or 696 or 697 or 698 or 699 or 700 or 701 or 702 or 703 or 704 or 705 or 706 or 707 or 708 or 709 or 710 or 711 or 712 or 713 or 714 or 715 or 716 or 717 or 860 or 861 or 862 or 863 or 864 or 865 or 866 or 867 or 868 or 869 or 870 or 871 or 872 or 873 or 874 or 875 or 876 or 877 or 878 or 879 or 880 or 881 or 882 or 883 or 884 or 885 or 886 or 887 or 888 or 889 or 890 or 891 or 892 or 893 or 894 or 895 or 896 or 897 or 898 or 899 or 900 or 901 or 902 or 903 or 904

922. community pharmacy.mp. or exp Community pharmacy/

923. exp Community pharmacy/ or exp Community pharmacies/ or retail pharmacy.mp.

924. outpatient pharmacy.mp.

925. community pharmacist.mp. or exp Community pharmacists/

926. outpatient pharmacist.mp.

927. store pharmacist.mp.

928. shop pharmacist.mp.

929. 1 or 919 or 922 or 923 or 924 or 925 or 926 or 927 or 928

930. exp medicines management/

931. exp Ambulatory care/

932. exp ergonomics/ or exp engineering/ or exp activity measurement/ or exp bionics/ or exp cybernetics/ or exp environmental technology/ or exp human biology/ or exp human physiology/ or exp job safety training/ or exp man machine systems/ or exp management techniques/ or exp office design/ or exp physiology/ or exp work study/ or exp working conditions/ or exp working environment/

933. exp human error/ or exp errors/ or exp human accident factors/ or exp medication errors/ or exp accident proneness/ or exp negligence/ or exp human fatigue/

934. exp Risk factors/

935. safe* therapy.mp.

936. drug safety.mp. or exp Drug safety/

937. 929 or 931

938. exp Handwriting/

939. drug interaction.mp. or exp Drug interactions/

940. therapeutic safety.mp.

941. exp causal analysis/ or exp causal models/ or exp causes/ or exp human accident factors/ or exp risk factors/ or exp aetiology/ or exp effects/ or exp motivation/

942. exp flexible working/ or exp full employment/ or full time work/ or exp part time work/ or exp temporary employment/ or exp voluntary work/ or exp "conditions of employment"/ or exp employment law/ or exp employment levels/ or exp employment policy/ or exp employment problems/ or exp employment status/ or exp human resources management/ or exp under employment/ or exp working conditions/ or exp workplace/

943. family stress/ or exp stress/

944. exp Occupational stress/

945. exp "Root cause analysis"/ or exp Risk factors/

946. exp incentives/ or exp motivation/ or exp achievement motivation/ or exp ambition/ or exp financial incentives/ or exp incentive systems/

947. measurement techniques/ or exp indicators/

948. Ergonomic.mp. or exp Ergonomics/

949. exp Motivation/ or exp Achievement motivation/ or motivation.mp.

950. exp aims & objectives/

951. evaluation/ or exp information systems evaluation/ or exp job evaluation/ or exp performance evaluation/ or exp policy evaluation/ or exp product evaluation/ or exp self evaluation/ or exp service evaluation/ or exp analysis/ or exp assessment/ or exp evaluation methods/ or exp inspection/ or exp judgement/ or exp management audit/ or exp management operations/ or exp measurement/ or exp monitoring/ or exp performance/ or exp progress control/

952. exp interdepartmental relationships/

953. 921 or 932 or 933 or 934 or 938 or 941 or 942 or 943 or 944 or 945 or 946 or 947 or 948 or 949 or 950 or 951 or 952

954. 918 or 930 or 935 or 936 or 939 or 940

955. exp qualitative research/ or Qualitative.mp. or exp Qualitative analysis/ or exp Qualitative techniques/

956. focus group.mp. or exp Focus groups/

957. exp Medical anthropology/ or exp Social anthropology/ or exp Anthropology/ or anthropology.mp.

958. exp Medical anthropology/ or exp Social anthropology/ or exp Anthropology/ or anthropology.mp.

959. interview.mp. or exp Interviews/

960. codes/ or exp coding systems/ or exp coding/

961. exp Job satisfaction/

962. 953 or 961

963. exp views/ or exp opinions/ or exp consumer views/ or exp staff views/ or exp attitudes/

964. exp Observation/ or observation.mp. or exp Non participant observation/ or exp Participant observation/

965. field work.mp. or exp Field work/

966. exp opinions/

967. 920 or 955 or 956 or 957 or 958 or 959 or 960 or 963 or 964 or 965 or 966

968. 937 and 954 and 962 and 967
