# Supplementary material for: Causes of medication errors in community pharmacies: A meta-ethnography and systematic review
Source: PLoS One. 2026 Jun 10;21(6):e0349120. doi: 10.1371/journal.pone.0349120 (PMC13252845; doi:10.1371/journal.pone.0349120)
Supplement: S4 File — (DOC) [file pone.0349120.s004.DOC]

| **Supplementary File 4. Results of the risk of bias assessment of the included studies** | | | | | | | | | | | |
| --- | --- | --- | --- | --- | --- | --- | --- | --- | --- | --- | --- |
| **Author & Year** | **Critical Appraisal Skills Tool Screening Questions** | | | | | | | | | | **Comments** |
|  | **1. Was there a clear statement of the aims of the research?** | **2. Is qualitative methodology appropriate?** | **3. Was the research design appropriate to address the aims of the research?** | **4. Was the recruitment strategy appropriate to the aims of the research?** | **5.** **Was the** **data collected in a way that addressed the research issue?** | **6. Has the relationship between researcher and participants been adequately considered?** | **7. Have ethical issues been taken into consideration?** | **8. Was the data analysis sufficiently rigorous?** | **9. Is there a clear statement of findings?** | **10. How valuable is the research?** |  |
|  | *Yes/No/Can’t tell* | | | | | | | | | |  |
| Phipps *et al.,* 2009 (1) | Yes | Yes | Yes | Yes | Can’t tell | Can’t tell | Yes | Can’t tell | Yes | Yes | Q 5: Unclear if researcher has discussed saturation of data  Q6: No information is available 1) If the researcher critically examined their own role, potential bias and influence during formulation of the research questions and data collection2) How the researcher responded to events during the study and whether they considered the implications of any changes in the research design  Q8: Unclear whether the researcher critically examined their own role, potential bias and influence during analysis and selection of data for presentation |
| Hincapie *et al.,* 2019 (2) | Yes | Yes | Yes | Can’t tell | Can’t tell | Can’t tell | Yes | Can’t tell | Yes | Yes | Q4: No information was provided on why only 10% random sample of comments received in the open text ﬁelds in each data set was selected.  Q 5: Unclear if researcher has discussed saturation of data  Q6: No information is available 1) If the researcher critically examined their own role, potential bias and influence during formulation of the research questions and data collection2) How the researcher responded to events during the study and whether they considered the implications of any changes in the research design  Q8: Unclear whether the researcher critically examined their own role, potential bias and influence during analysis and selection of data for presentation |
| Phipps *et al.,* 2018 (3) | Yes | Yes | Yes | Yes | Yes | Yes | Yes | Yes | Yes | Yes |  |
| Odukoya *et al.,* 2014 (4) | Yes | Yes | Yes | Yes | Can’t tell | Can’t tell | Yes | Can’t tell | Yes | Yes | Q 5: Unclear if researcher has discussed saturation of data  Q6: No information is available 1) If the researcher critically examined their own role, potential bias and influence during formulation of the research questions and data collection2) How the researcher responded to events during the study and whether they considered the implications of any changes in the research design  Q8: Unclear whether the researcher critically examined their own role, potential bias and influence during analysis and selection of data for presentation |
| Al juffali *et al.,* 2019 (5) | Yes | Yes | Yes | Yes | Yes | Can’t tell | Yes | Yes | Yes | Yes | Q6: No information is available 1) If the researcher critically examined their own role, potential bias and influence during formulation of the research questions and data collection2) How the researcher responded to events during the study and whether they considered the implications of any changes in the research design |
| Odukoya and Chui, 2013 (6) | Yes | Yes | Yes | Yes | Yes | Can’t tell | Yes | Yes | Yes | Yes | Q6: No information is available 1) If the researcher critically examined their own role, potential bias and influence during formulation of the research questions and data collection2) How the researcher responded to events during the study and whether they considered the implications of any changes in the research design |
| Harvey *et al.,* 2015 (7) | Yes | Yes | Yes | Yes | Can’tell | Can’t tell | Yes | Yes | Yes | Yes | Q 5: Unclear if researcher has discussed saturation of data  Q6: No information is available 1) If the researcher critically examined their own role, potential bias and influence during formulation of the research questions and data collection2) How the researcher responded to events during the study and whether they considered the implications of any changes in the research design |
| Lester et al.,2019 (8) | Yes | Yes | Yes | Yes | Yes | Can’t tell | Can’t tell | Yes | Yes | Yes | Q6: No information is available 1) If the researcher critically examined their own role, potential bias and influence during formulation of the research questions and data collection2) How the researcher responded to events during the study and whether they considered the implications of any changes in the research design Q7: No information if approval has been sought from the ethics committee` |
| Clabaugh et al., 2021 (9) | Yes | Yes | No | I can’t tell | No | I can’t tell | Yes | Yes | Yes | Yes | Q3: Researchers did not discuss how they decided which method to use  Q4: No enough information is provided on why the participants they selected to participate in the study were the most appropriate  Q5: There was no justification on the methods chosen for data collection and data saturation was not discussed.  Q6: No information is available 1) If the researcher critically examined their own role, potential bias and influence during formulation of the research questions and data collection2) How the researcher responded to events during the study and whether they considered the implications of any changes in the research design |
| Wang *et al.,* 2024 (10) | Yes | Yes | No | Yes | No | I can’t tell | I can’t tell | Yes | Yes | Yes | Q3: Researchers did not discuss how they decided which method to use  Q5: There was no justification on the methods chosen for data collection and data saturation was not mentioned.  Q6: No information is available 1) If the researcher critically examined their own role, potential bias and influence during formulation of the research questions and data collection2) How the researcher responded to events during the study and whether they considered the implications of any changes in the research design  Q7: No information is provided about ethical approval |
| Odukoya and Chui, 2012 (11) | Yes | Yes | Yes | Yes | Yes | I can’t tell | Yes | Yes | Yes | Yes | Q6: No information is available 1) If the researcher critically examined their own role, potential bias and influence during formulation of the research questions and data collection2) How the researcher responded to events during the study and whether they considered the implications of any changes in the research design |
| Jones *et al.,* 2018 (12) | Yes | Yes | Yes | Yes | Yes | I can’t tell | Yes | Yes | Yes | Yes | Q6: No information is available 1) If the researcher critically examined their own role, potential bias and influence during formulation of the research questions and data collection2) How the researcher responded to events during the study and whether they considered the implications of any changes in the research design |
| Whitaker *et al.,* 2024(13) | Yes | Yes | Yes | Yes | Yes | I cant tell | Yes | Yes | Yes | Yes | Q6: No information is available 1) If the researcher critically examined their own role, potential bias and influence during formulation of the research questions and data collection2) How the researcher responded to events during the study and whether they considered the implications of any changes in the research design |

**References**

1. Phipps DL, Noyce PR, Parker D, Ashcroft DM. Medication safety in community pharmacy: a qualitative study of the sociotechnical context. BMC Health Serv Res. 2009;9:158.

2. Hincapie AL, Alamer A, Sears J, Warholak TL, Goins S, Weinstein SD. A Quantitative and Qualitative Analysis of Electronic Prescribing Incidents Reported by Community Pharmacists. Appl Clin Inform. 2019;10(3):387-94.

3. Phipps DL, Jones CEL, Parker D, Ashcroft DM. Organizational conditions for engagement in quality and safety improvement: a longitudinal qualitative study of community pharmacies. BMC Health Serv Res. 2018;18(1):783.

4. Odukoya OK, Stone JA, Chui MA. E-prescribing errors in community pharmacies: exploring consequences and contributing factors. Int J Med Inform. 2014;83(6):427-37.

5. Al Juffali L, Al-Aqeel S, Knapp P, Mearns K, Family H, Watson M. Using the Human Factors Framework to understand the origins of medication safety problems in community pharmacy: A qualitative study. Res Social Adm Pharm. 2019;15(5):558-67.

6. Odukoya OK, Chui MA. e-Prescribing: characterisation of patient safety hazards in community pharmacies using a sociotechnical systems approach. BMJ Qual Saf. 2013;22(10):816-25.

7. Harvey J, Avery AJ, Ashcroft D, Boyd M, Phipps DL, Barber N. Exploring safety systems for dispensing in community pharmacies: focusing on how staff relate to organizational components. Res Social Adm Pharm. 2015;11(2):216-27.

8. Lester CA, Kessler JM, Modisett T, Chui MA. A text mining analysis of medication quality related event reports from community pharmacies. Res Social Adm Pharm. 2019;15(7):845-51.

9. Clabaugh M, Newlon JL, Illingworth Plake KS. Perceptions of working conditions and safety concerns in community pharmacy. J Am Pharm Assoc (2003). 2021;61(6):761-71.

10. Wang Y, Ram SS, Scahill S. Understanding Risk Factors for Complaints Against Pharmacists: A Content Analysis. J Patient Saf. 2024;20(4):e18-e28.

11. Odukoya O, Chui MA. Retail pharmacy staff perceptions of design strengths and weaknesses of electronic prescribing. J Am Med Inform Assoc. 2012;19(6):1059-65.

12. Jones CEL, Phipps DL, Ashcroft DM. Understanding procedural violations using Safety-I and Safety-II: The case of community pharmacies. Saf Sci. 2018;105:114-20.

13. Whitaker M, Lester C, Rowell B. Handing Off Electronic Prescription Data From Prescribers to Community Pharmacies: A Qualitative Analysis of Pharmacy Staff Perspectives. Journal of Patient Safety. 2024;20(6):397-403.
