# Supplementary material for: Causes of medication errors in community pharmacies: A meta-ethnography and systematic review
Source: PLoS One. 2026 Jun 10;21(6):e0349120. doi: 10.1371/journal.pone.0349120 (PMC13252845; doi:10.1371/journal.pone.0349120)
Supplement: S5 File — (DOC) [file pone.0349120.s005.DOC]

| **Supplementary File 5. CERQual assessments** | | | | |
| --- | --- | --- | --- | --- |
| **Review finding** | | **CERQual**  **Assessment of**  **Confidence in the**  **Evidence** | **Explanation of CERQual**  **Assessment** | **Studies**  **Contributing to**  **the Review**  **Finding** |
| **Pharmacist related factors** | Pharmacist competence and experience | Moderate | **Assessment of methodological limitations:** Minor concerns  Two studies did not provide information on whether the researchers critically examined their own roles, potential biases, or influence on the research, nor how they responded to events during the study or considered the implications of any changes to the research design.  In one study, the researchers did not explain how they selected their methodological approach, failed to justify the data collection methods used, and did not address data saturation. And no information was provided about ethical approval in this study. | (1, 2) |
|  |  |  | **Assessment of coherence**  No/very minor concerns as the studies had clear underlying data supporting the finding. |  |
|  |  |  | **Assessment of adequacy**  Minor concerns as two studies were contributing to this finding. |  |
|  |  |  | **Assessment of relevance**  No/very minor concerns as the studies were conducted in community pharmacies. |  |
| **The environment within the pharmacy** | Limitations of pharmacy space and medication storage | High | **Assessment of methodological limitations**  Minor concerns  In one study researchers did not discuss how they decided which method to use. In one study, there was no justification on the methods chosen for data collection and data saturation was not mentioned. In one study, it was unclear if researcher has discussed saturation of data. In three studies no information was available 1) If the researcher critically examined their own role, potential bias and influence during formulation of the research questions and data collection2) How the researcher responded to events during the study and whether they considered the implications of any changes in the research design.  In one study, it was unclear whether the researcher critically examined their own role, potential bias and influence during analysis and selection of data for presentation. In one study, no information was provided about ethical approval. | (1-3) |
|  |  |  | **Assessment of coherence** No/very minor concerns as the studies had clear underlying data supporting the finding. |  |
|  |  |  | **Assessment of adequacy**  Minor concerns as three studies were contributing to this finding. |  |
|  |  |  | **Assessment of relevance** No/very minor concerns as the studies were conducted in community pharmacies. |  |
|  | Challenges with medication packaging | Moderate | **Assessment of methodological limitations**  Minor concerns  In one study, researchers did not discuss how they decided which method to use. In one study, there was no justification on the methods chosen for data collection and data saturation was not mentioned. In one study, it was unclear if researcher has discussed saturation of data. In two studies, no information was available 1) If the researcher critically examined their own role, potential bias and influence during formulation of the research questions and data collection2) How the researcher responded to events during the study and whether they considered the implications of any changes in the research design. In one study, it was unclear whether the researcher critically examined their own role, potential bias and influence during analysis and selection of data for presentation. In one study, no information was provided about ethical approval. | (2, 3) |
|  |  |  | **Assessment of coherence**  No/very minor concerns as the studies had clear underlying data supporting the finding. |  |
|  |  |  | **Assessment of adequacy**  Minor concerns as two studies were contributing to this finding. |  |
|  |  |  | **Assessment of relevance**  No/very minor concerns as the studies were conducted in community pharmacies. |  |
|  | Managing typical interruptions within the pharmacy | High | **Assessment of methodological limitations**  Minor concerns.  In two studies, researchers did not discuss how they decided which method to use. In one study, no enough information is provided on why the participants they selected to participate in the study were the most appropriate. In four studies, information about data saturation was either unclear or missing. In two studies, there was no justification on the methods chosen for data collection. In six studies, no information was available 1) If the researcher critically examined their own role, potential bias and influence during formulation of the research questions and data collection and 2) How the researcher responded to events during the study and whether they considered the implications of any changes in the research design. In one study, no information was provided about ethical approval. And in another study, it was unclear whether the researcher critically examined their own role, potential bias and influence during analysis and selection of data for presentation. | (2-8) |
|  |  |  | **Assessment of coherence**  No/very minor concerns as the studies had clear underlying data supporting the finding. |  |
|  |  |  | **Assessment of adequacy**  No/very minor concerns as 7 studies were contributing to this finding. |  |
|  |  |  | **Assessment of relevance**  No/very minor concerns as the studies were conducted in community pharmacies. |  |
| **Management and financial related factors** | Workforce and resource pressures | High | **Assessment of methodological limitations**  Minor concerns.  In two studies, researchers did not discuss how they decided which method to use. In one study, no enough information is provided on why the participants they selected to participate in the study were the most appropriate. In four studies, information about data saturation was either unclear or missing. In two studies, there was no justification on the methods chosen for data collection. In six studies, no information is available 1) If the researcher critically examined their own role, potential bias and influence during formulation of the research questions and data collection and 2) How the researcher responded to events during the study and whether they considered the implications of any changes in the research design. In one study, no information was provided about ethical approval. And in another study, it was unclear whether the researcher critically examined their own role, potential bias and influence during analysis and selection of data for presentation. | (1-6, 8) |
|  |  |  | **Assessment of coherence**  No/very minor concerns as the studies had clear underlying data supporting the finding. |  |
|  |  |  | **Assessment of adequacy**  No/very minor concerns as seven studies were contributing to this finding. |  |
|  |  |  | **Assessment of relevance**  No/very minor concerns as the studies were conducted in community pharmacies. |  |
|  | Management  pressure on pharmacists | High | **Assessment of methodological limitations**  Minor concerns  In three studies, no information was available 1) If the researcher critically examined their own role, potential bias and influence during formulation of the research questions and data collection 2) How the researcher responded to events during the study and whether they considered the implications of any changes in the research design. In two studies, data saturation was not discussed or unclear. In one study, there was no justification on the methods chosen for data collection and no enough information was provided on why the participants they selected to participate in the study were the most appropriate. | (1, 4, 5) |
|  |  |  | **Assessment of coherence**  No/very minor concerns as the studies had clear underlying data supporting the finding. |  |
|  |  |  | **Assessment of adequacy**  No/very minor concerns as three studies were contributing to this finding. |  |
|  |  |  | **Assessment of relevance** No/very minor concerns as the studies were conducted in community pharmacies. |  |
|  | Impact of Commercial, and Financial Pressures, Insurance and Incentives on Pharmacist and Physician Practices | High | **Assessment of methodological limitations**  Minor concerns  In three studies no information was available 1) If the researcher critically examined their own role, potential bias and influence during formulation of the research questions and data collection 2) How the researcher responded to events during the study and whether they considered the implications of any changes in the research design.  In two studies, data saturation was not discussed or was unclear. In one study, researchers did not discuss how they decided which method to use, not enough information is provided on why the participants they selected to participate in the study were the most appropriate and there was no justification on the methods chosen for data collection. In one study, it was unclear whether the researcher critically examined their own role, potential bias and influence during analysis and selection of data for presentation. | (1, 3, 4) |
|  |  |  | **Assessment of coherence**  No/very minor concerns as the studies had clear underlying data supporting the finding |  |
|  |  |  | **Assessment of adequacy**  No/very minor concerns as three studies were contributing to this finding |  |
|  |  |  | **Assessment of relevance**  No/very minor concerns as the studies were conducted in community pharmacies |  |
| **Organizational and social environment within the pharmacy** | Error mitigation policies and processes | High | **Assessment of methodological limitations**  Minor concerns.  In two studies, researchers did not discuss how they decided which method to use. In one study, no enough information is provided on why the participants they selected to participate in the study were the most appropriate. In four studies, information about data saturation was either unclear or missing. In two studies, there was no justification on the methods chosen for data collection. In six studies, no information is available 1) If the researcher critically examined their own role, potential bias and influence during formulation of the research questions and data collection and 2) How the researcher responded to events during the study and whether they considered the implications of any changes in the research design. In one study, no information was provided about ethical approval. And in another study, it was unclear whether the researcher critically examined their own role, potential bias and influence during analysis and selection of data for presentation. | (1-6, 8) |
|  |  |  | **Assessment of coherence**  No/very minor concerns as the studies had clear underlying data supporting the finding. |  |
|  |  |  | **Assessment of adequacy**  No/very minor concerns as seven studies were contributing to this finding. |  |
|  |  |  | **Assessment of relevance**  No/very minor concerns as the studies were conducted in community pharmacies. |  |
|  | Human errors  in pharmacy practice | High | **Assessment of methodological limitations**  Minor concerns  In four studies no information was available 1) If the researcher critically examined their own role, potential bias and influence during formulation of the research questions and data collection and 2) How the researcher responded to events during the study and whether they considered the implications of any changes in the research design.  In one study, it was unclear if researcher has discussed saturation of data and whether the researcher critically examined their own role, potential bias and influence during analysis and selection of data for presentation.  In one study, there was no information if approval has been sought from the ethics committee, researchers did not discuss how they decided which method to use and there was no justification on the methods chosen for data collection and data saturation was not mentioned. | (2, 3, 9, 10) |
|  |  |  | **Assessment of coherence**  No/very minor concerns as the studies had clear underlying data supporting the finding |  |
|  |  |  | **Assessment of adequacy**  No/very minor concerns as four studies were contributing to this finding. |  |
|  |  |  | **Assessment of relevance**  No/very minor concerns as the studies were conducted in community pharmacies |  |
|  | Communication challenges | High | **Assessment of methodological limitations**  No/very minor concerns  In four studies no information was available 1) If the researcher critically examined their own role, potential bias and influence during formulation of the research questions and data collection and 2) How the researcher responded to events during the study and whether they considered the implications of any changes in the research design.  In one study, there was no information if approval has been sought from the ethics committee, researchers did not discuss how they decided which method to use and there was no justification on the methods chosen for data collection and data saturation was not mentioned.  In one study, it was unclear if researcher has discussed saturation of data and whether the researcher critically examined their own role, potential bias and influence during analysis and selection of data for presentation | (1-3, 7, 8) |
|  |  |  | **Assessment of coherence**  No/very minor concerns as the studies had clear underlying data supporting the finding |  |
|  |  |  | **Assessment of adequacy**  No/very minor concerns as five studies were contributing to this finding. |  |
|  |  |  | **Assessment of relevance**  No/very minor concerns as the studies were conducted in community pharmacies |  |
|  | Unrealistic expectations about pharmacist practice | High | **Assessment of methodological limitations**  Minor concerns  In five studies no information was available 1) If the researcher critically examined their own role, potential bias and influence during formulation of the research questions and data collection 2) How the researcher responded to events during the study and whether they considered the implications of any changes in the research design.  In two studies, researchers did not discuss how they decided which method to use. In two studies, there was no justification on the methods chosen for data collection and data saturation was not discussed. In one study, it was unclear if researcher has discussed saturation of data  And it was unclear whether the researcher critically examined their own role, potential bias and influence during analysis and selection of data for presentation. In one study, no enough information was provided on why the participants they selected to participate in the study were the most appropriate. In one study, no information was provided about ethical approval. | (1-4, 6) |
|  |  |  | **Assessment of coherence**  No/very minor concerns as the studies had clear underlying data supporting the finding |  |
|  |  |  | **Assessment of adequacy**  No/very minor concerns as five studies were contributing to this finding. |  |
|  |  |  | **Assessment of relevance**  No/very minor concerns as the studies were conducted in community pharmacies |  |
| **Challenges with digital technologies** | Prescription input error, system translation and incompatibility issues | High | **Assessment of methodological limitations**  Minor concerns  In three studies, no information was available 1) If the researcher critically examined their own role, potential bias and influence during formulation of the research questions and data collection 2) How the researcher responded to events during the study and whether they considered the implications of any changes in the research design. In two studies, it was unclear if researcher has discussed saturation of data and it was unclear whether the researcher critically examined their own role, potential bias and influence during analysis and selection of data for presentation. In one study, no information was provided on why only 10% of comments received in the open text ﬁelds in each data set was selected. | (11-13) |
|  |  |  | **Assessment of coherence**  No/very minor concerns as the studies had clear underlying data supporting the finding |  |
|  |  |  | **Assessment of adequacy**  No/very minor concerns as three studies were contributing to this finding. |  |
|  |  |  | **Assessment of relevance**  No/very minor concerns as the studies were conducted in community pharmacies |  |
|  | Software limitations, including e-prescription control and management of outdated notes and prescriptions | High | **Assessment of methodological limitations**  In six studies, no information was available 1) If the researcher critically examined their own role, potential bias and influence during formulation of the research questions and data collection 2) How the researcher responded to events during the study and whether they considered the implications of any changes in the research design.  In three studies, it was unclear if data saturation was achieved. In two studies, it was unclear whether the researcher critically examined their own role, potential bias and influence during analysis and selection of data for presentation. In one study, no information was provided on why only 10% random sample of comments received in the open text ﬁelds in each data set was selected. In one study, researchers did not discuss how they decided which method to use, there was no justification on the methods chosen for data collection and data saturation was not mentioned. And no information was provided about ethical approval. | (2, 5, 9, 11-13) |
|  |  |  | **Assessment of coherence**  No/very minor concerns as the studies had clear underlying data supporting the finding |  |
|  |  |  | **Assessment of adequacy**  No/very minor concerns as six studies were contributing to this finding. |  |
|  |  |  | **Assessment of relevance**  No/very minor concerns as the studies were conducted in community pharmacies |  |
|  | Absence of automation and technology in community pharmacies and limited integration with other healthcare institutions | High | **Assessment of methodological limitations**  Minor concerns  In one study researchers did not discuss how they decided which method to use. In one study, there was no justification on the methods chosen for data collection and data saturation was not mentioned. In one study, it was unclear if researcher has discussed saturation of data. In three studies no information was available 1) If the researcher critically examined their own role, potential bias and influence during formulation of the research questions and data collection2) How the researcher responded to events during the study and whether they considered the implications of any changes in the research design.  In one study, it was unclear whether the researcher critically examined their own role, potential bias and influence during analysis and selection of data for presentation. In one study, no information was provided about ethical approval. | (1-3) |
|  |  |  | **Assessment of coherence** No/very minor concerns as the studies had clear underlying data supporting the finding. |  |
|  |  |  | **Assessment of adequacy**  Minor concerns as three studies were contributing to this finding. |  |
|  |  |  | **Assessment of relevance** No/very minor concerns as the studies were conducted in community pharmacies. |  |

**References**

1. Al Juffali L, Al-Aqeel S, Knapp P, Mearns K, Family H, Watson M. Using the Human Factors Framework to understand the origins of medication safety problems in community pharmacy: A qualitative study. Res Social Adm Pharm. 2019;15(5):558-67.

2. Wang Y, Ram SS, Scahill S. Understanding Risk Factors for Complaints Against Pharmacists: A Content Analysis. J Patient Saf. 2024;20(4):e18-e28.

3. Phipps DL, Noyce PR, Parker D, Ashcroft DM. Medication safety in community pharmacy: a qualitative study of the sociotechnical context. BMC Health Serv Res. 2009;9:158.

4. Clabaugh M, Newlon JL, Illingworth Plake KS. Perceptions of working conditions and safety concerns in community pharmacy. J Am Pharm Assoc (2003). 2021;61(6):761-71.

5. Harvey J, Avery AJ, Ashcroft D, Boyd M, Phipps DL, Barber N. Exploring safety systems for dispensing in community pharmacies: focusing on how staff relate to organizational components. Res Social Adm Pharm. 2015;11(2):216-27.

6. Jones CEL, Phipps DL, Ashcroft DM. Understanding procedural violations using Safety-I and Safety-II: The case of community pharmacies. Saf Sci. 2018;105:114-20.

7. Odukoya OK, Chui MA. e-Prescribing: characterisation of patient safety hazards in community pharmacies using a sociotechnical systems approach. BMJ Qual Saf. 2013;22(10):816-25.

8. Phipps DL, Jones CEL, Parker D, Ashcroft DM. Organizational conditions for engagement in quality and safety improvement: a longitudinal qualitative study of community pharmacies. BMC Health Serv Res. 2018;18(1):783.

9. Lester CA, Kessler JM, Modisett T, Chui MA. A text mining analysis of medication quality related event reports from community pharmacies. Res Social Adm Pharm. 2019;15(7):845-51.

10. Whitaker M, Lester C, Rowell B. Handing Off Electronic Prescription Data From Prescribers to Community Pharmacies: A Qualitative Analysis of Pharmacy Staff Perspectives. Journal of Patient Safety. 2024;20(6):397-403.

11. Hincapie AL, Alamer A, Sears J, Warholak TL, Goins S, Weinstein SD. A Quantitative and Qualitative Analysis of Electronic Prescribing Incidents Reported by Community Pharmacists. Appl Clin Inform. 2019;10(3):387-94.

12. Odukoya O, Chui MA. Retail pharmacy staff perceptions of design strengths and weaknesses of electronic prescribing. J Am Med Inform Assoc. 2012;19(6):1059-65.

13. Odukoya OK, Stone JA, Chui MA. E-prescribing errors in community pharmacies: exploring consequences and contributing factors. Int J Med Inform. 2014;83(6):427-37.
